# Novel genotype–phenotype associations demonstrated by high-throughput sequencing in patients with hypertrophic cardiomyopathy

**DOI:** 10.1136/heartjnl-2014-306387

**Published:** 2014-10-28

**Authors:** Luis R Lopes, Petros Syrris, Oliver P Guttmann, Constantinos O'Mahony, Hak Chiaw Tang, Chrysoula Dalageorgou, Sharon Jenkins, Mike Hubank, Lorenzo Monserrat, William J McKenna, Vincent Plagnol, Perry M Elliott

**Affiliations:** 1 UCL Institute of Cardiovascular Science, London, UK; 2 The London Chest Hospital, London, UK; 3 National Heart Centre, Singapore, Singapore; 4 UCL Genomics, Department of Molecular Haematology and Cancer Biology, UCL Institute of Child Health, London, UK; 5 Instituto de Investigación Biomédica de la Universidad de A Coruña (INIBIC), Complexo Hospitalario Universitario de A Coruña (CHUAC)-Universidad de A Coruña, A Coruña, Spain; 6 UCL Genetics Institute, London, UK

**Keywords:** GENETICS

## Abstract

**Objective:**

A predictable relation between genotype and disease expression is needed in order to use genetic testing for clinical decision-making in hypertrophic cardiomyopathy (HCM). The primary aims of this study were to examine the phenotypes associated with sarcomere protein (SP) gene mutations and test the hypothesis that variation in non-sarcomere genes modifies the phenotype.

**Methods:**

Unrelated and consecutive patients were clinically evaluated and prospectively followed in a specialist clinic. High-throughput sequencing was used to analyse 41 genes implicated in inherited cardiac conditions. Variants in SP and non-SP genes were tested for associations with phenotype and survival.

**Results:**

874 patients (49.6±15.4 years, 67.8% men) were studied; likely disease-causing SP gene variants were detected in 383 (43.8%). Patients with SP variants were characterised by younger age and higher prevalence of family history of HCM, family history of sudden cardiac death, asymmetric septal hypertrophy, greater maximum LV wall thickness (all p values<0.0005) and an increased incidence of cardiovascular death (p=0.012). Similar associations were observed for individual SP genes. Patients with *ANK2* variants had greater maximum wall thickness (p=0.0005). Associations at a lower level of significance were demonstrated with variation in other non-SP genes.

**Conclusions:**

Patients with HCM caused by rare SP variants differ with respect to age at presentation, family history of the disease, morphology and survival from patients without SP variants. Novel associations for SP genes are reported and, for the first time, we demonstrate possible influence of variation in non-SP genes associated with other forms of cardiomyopathy and arrhythmia syndromes on the clinical phenotype of HCM.

## Introduction

Hypertrophic cardiomyopathy (HCM) is a common autosomal dominant genetic trait associated with sudden cardiac death (SCD) and progressive heart failure.^1^ ^2^ Patients are routinely offered genetic testing in order to provide them with information about the likely impact of disease on their lives and facilitate lifestyle and medical interventions that improve prognosis.^2^ ^3^ However, for this strategy to succeed, there must be a predictable relation between specific genotypes and disease expression.

In around 50% of cases, HCM is caused by mutations in genes coding for sarcomere or sarcomere-related genes.^4^ So far, the most commonly reported genotype–phenotype associations are those that relate to the presence or absence of sarcomere protein (SP) gene mutations rather than mutations in specific genes.^5^ ^6^ A number of studies have suggested that some mutations are associated with reduced survival, but these findings are inconsistent and fail to account for the often dramatic variation in clinical phenotypes seen in individuals with the same genetic variant.^w1–w7^ 7 ^w8^ 8 ^w9^ 9 ^w10^ ^w11^ 10 ^w12^ 11 ^w13^ ^w14^ 12 13 ^w15–w18^ 14 ^w19^ 15 16

Several studies have examined the role of common genetic variation on the expression of sarcomere mutations using genome-wide association studies or a candidate gene approach, but most have failed to show any major effect on disease expression^.w20–w22^ 17 18 HCM cases (as well as controls) also carry rare variants in genes coding for desmosomal, ion-channel and other proteins implicated in inherited heart disease^19^ but their relevance to disease expression is unknown.

The hypothesis of this study is that rare variants in sarcomere genes and also in non-sarcomere genes implicated in other forms of inherited cardiac disorders (for which sequence data are available in our study) modify the clinical characteristics and severity of HCM.

## Methods

### Study population and design, clinical evaluation and sample collection

The study was approved by the University College London (UCL)/UCL Hospitals (UCLH) Joint Research Ethics Committee. Before enrolment, all patients provided written informed consent and received genetic counselling in accordance with international guidelines.^3^

An observational, retrospective, longitudinal cohort study design was used. The study population comprised unrelated and consecutively evaluated patients with HCM referred to the Inherited Cardiovascular Disease Unit at The Heart Hospital, UCLH, London, UK. Clinical evaluation included a personal and family history, physical examination, 12 lead ECG, echocardiography, symptom limited upright exercise testing with simultaneous respiratory gas analysis (cardiopulmonary exercise test) and ambulatory ECG monitoring as previously described.^20^ HCM was diagnosed in probands when the maximum left ventricular (LV) wall thickness (MLVWT) on 2D echocardiography measured 15 mm or more in at least one myocardial segment or when MLVWT exceeded 2 SDs corrected for age, size and gender in the absence of other diseases that could explain the hypertrophy.^21^ In individuals with unequivocal disease in a first degree relative, diagnosis was made using extended familial criteria for HCM.^22^ Ethnicity was self-reported and classified using a modified National Health Service ethnic categorisation. Patients were evaluated every 6–12 months or earlier if there was a clinical event. Initial evaluation and follow-up data were collected prospectively and registered in a relational database. The definitions of severe LV hypertrophy, family history of SCD, syncope, non-sustained ventricular tachycardia (NSVT) and abnormal blood pressure response were as previously described.^23^

### Targeted gene enrichment and high-throughput sequencing

Blood samples were collected at initial evaluation and DNA was isolated from peripheral blood lymphocytes using standard methods. The sequencing methodology has been reported in detail previously.^19^ In summary, the protocol was designed to screen 2.1 Mbp of genomic DNA sequence per patient, covering coding, intronic and selected regulatory regions of 20 genes known to be associated with HCM and dilated cardiomyopathy (*MYH7, MYBPC3, TNNT2, TNNI3, MYL2, MYL3, ACTC1, TPM1, TNNC1, MYH6, CSRP3, DES, TCAP, PDLIM3, PLN, LDB3, LMNA, VCL, RBM20* and *TTN*), 10 genes implicated in arrhythmia syndromes/ion-channel disease (*RYR2, KCNQ1, KCNH2, SCN5A, KCNE1, KCNE2, ANK2, CASQ2, CAV3* and *KCNJ2*), seven genes associated with arrhythmogenic right ventricular cardiomyopathy (*PKP2, DSC2, DSG2, JUP, DSP, TMEM43* and *TGFß3*) and a further four candidate genes (*GJA1, PLEC, PNN* and *PKP4*) which were not analysed in this work.^19^ Analysis of titin (*TTN*) variants and their effect on phenotype is ongoing and will be reported in a separate paper.

### Bioinformatic analysis

Paired-end reads were aligned using Novoalign software V.2.7.19 on the human reference genome build hg19. Duplicate reads were flagged using the Picard MarkDuplicate tool. Our calling strategy followed closely the Genome Analysis Toolkit (GATK) best practices as of January 2014. Briefly, following BAM file compression using the GATK ReduceReads module,^24^ multisample calling was performed on all probands jointly with a set of 1492 unrelated whole exomes (UCL-exome consortium) using the GATK Unified Genotyper.^24^ After GATK variant recalibration (separately for SNPs and indels), calls were annotated using the ANNOVAR software (with the Ensembl gene definitions).^w23^ For all association tests, we filtered variants for the GATK recalibration PASS filter.

Candidate variants for further analysis were defined using frequency and predicted functional effect. For the functional filter, exonic non-synonymous, loss-of-function and splice-site variants were included. Sequence data were filtered using a minor allele frequency threshold of ≤0.2% based on the NHLBI exome variant server data (computed through the ANNOVAR annotations). To provide a more accurate estimate of variant frequency in controls that is not affected by potential differences in calling strategy in the NHLBI dataset, we randomly selected 25% of the 1492 UCL-exome samples as an ‘external control set’ and removed variants that appeared more than twice in these 372 ‘external controls’. These samples were only used to define a variant frequency and not included in the subsequent association test, to avoid a previously noted statistical issue, where variant frequency is defined in the same set that is used for case control testing.^w24^ Variants present in the dbSNP build 137 database^w25^ and published in the literature were identified. In silico prediction of pathogenicity for novel missense variants was performed using Polyphen2, SIFT and Condel.^w26 w27^ 25 A variant was predicted to be pathogenic if classified as ‘damaging’ by SIFT and simultaneously ‘possibly’ or ‘probably damaging’ by Polyphen2, or if predicted to be damaging by Condel.

### Summary statistics for genotype–phenotype associations

R (V.3.0.0) and SPSS (V.22.0.0.0, IBM Corp.) were used for the analyses. Clinical phenotype data are presented as frequency (and percentage) for non-continuous variables and mean±SD or median and IQR for continuous variables where appropriate. Normally distributed continuous variables were compared using unpaired two-tailed Student's t test. Multiple groups were compared using analysis of variance. Categorical variables were compared using χ^2^ or Fisher exact tests. When appropriate, non-parametric tests were used.

Group comparisons were made for the prevalence and severity of each phenotypic trait (at baseline and final follow-up) in patients with and without a rare variant in one or more of the eight most common SP genes (*MYH7, MYBPC3, TNNI3, TNNT2, MYL2, MYL3, ACTC1* and *TPM1*). We also compared the prevalence and severity of each phenotypic trait in patients carrying only one versus more than one variant in SP genes. The same comparisons were made for the presence and absence of rare variation in non-SP genes in the whole cohort and in the subgroup of individuals with a disease-causing SP gene mutation.

### Multiple testing correction strategy

For each trait of interest we tested the effect of variants in eight SP genes and 28 non-SP genes. Therefore, a nominal p value of 0.05 was not appropriate. In addition, the Bonferroni correction for the number of phenotypes multiplied by the number of genes is too stringent because it tests the global null of no association between any pair of gene/trait. We therefore took an intermediate approach, correcting the analysis of each phenotype for the number of gene tests. For SP genes, we performed nine tests (one per SP gene, plus an additional test for all SP mutations combined). Therefore, we used p<0.0056, which is 0.05/9. For non-SP genes, we corrected for 28 tests, which translates into p<0.0018=0.05/28. Data on associations that did not fulfil these thresholds but met a nominal p value of <0.05 are presented in the Results section and online supplementary files.

### Survival analysis

Definition of endpoints in the survival analyses was as previously described.^23^ Survival from cardiovascular death (a composite of SCD and death from heart failure or stroke) and SCD or equivalent (appropriate implantable cardioverter-defibrillator (ICD) shock) was modelled using Kaplan–Meier analysis and log-rank test from the first clinical evaluation at The Heart Hospital and from birth.

## Results

### Study population

In all, 874 unrelated and consecutive patients with HCM were studied. Mean follow-up time was 4.8±3.5 years (0–16.8 years). Table 1 summarises the demographic and clinical characteristics of the patients at initial evaluation and their outcomes.

**Table 1:** Demographic and clinical characteristics of the study cohort

	Frequency (percentage) or mean±SD (range) or median (IQR)
Demographics	
Age at initial evaluation (years)	49.6±15.4 (6–87)
Male	590/874 (67.8%)
Ethnicity	
Caucasian	622 (71.2%)
Indian and other Asian	68 (7.8%)
African/Caribbean	39 (4.5%)
Chinese	6 (0.7%)
Other	20 (2.3%)
Not reported	119 (13.6%)
Presentation	
Family history of HCM	226/853 (26.5%)
Family history of SCD	182/872 (20.9%)
NYHA class III or IV	100/850 (11.8%)
Syncope	140/856 (16.4%)
Chest pain	205/854 (24.0%)
Initial ECG	
Atrial fibrillation	43/874 (4.9%)
PR interval (ms)	174.8±32.4 (108–320)
QRS duration (ms)	101.0±25.5 (64–238)
Initial CPEX	
SBP rest (mm Hg)	128.8±21.0 (80–210)
SBP response to exercise (mmHg)	48.5±24.2 (-5–150)
Abnormal SBP response to exercise	92/662 (13.9%)
Initial echocardiography	
Maximal LV wall thickness (mm)	18.5±4.4 (9–38)
Severe LVH (≥30 mm)	17/601 (2.8%)
Right ventricular hypertrophy (>5 mm)	184/864 (21.3%)
Asymmetric septal hypertrophy pattern	643/850 (75.6%)
Left atrial diameter (mm)	44.0±7.5 (18–90)
LV end-diastolic diameter (mm)	45.9±5.9 (29–65)
LV dilatation (>55 mm)	38/851 (4.5%)
LV end-systolic diameter (mm)	28.5±5.6 (9–50)
Fractional shortening (%)	38.3±8.2 (16–70)
Systolic dysfunction (≤25% FS)	29/829 (3.5%)
E wave deceleration time (ms)	221.0 (184–268)
Mitral regurgitation—moderate/severe	163/851 (19.2%)
Peak LVOT gradient (mm Hg)	12.0 (4.0–60.0)
LVOT gradient >30 mm Hg	328/812 (40.4%)
NSVT—Holter	127/566 (22.4%)
Follow-up	
New-onset atrial fibrillation	216/874 (24.7%)
Implantable cardioverter-defibrillator	177/874 (20.3%)
Myectomy	130/874 (14.9%)
Alcohol septal ablation	46/874 (5.3%)
Myectomy and/or alcohol septal ablation and/or pacemaker implantation for LVOT gradient reduction	182/874 (20.8%)
Cardiovascular death	25/874 (2.9%)
SCD	16/874 (1.8%)

Total N=874.

CPEX, cardiopulmonary exercise test; FS, fractional shortening; HCM, hypertrophic cardiomyopathy; LVH, LV hypertrophy; LVOT, LV outflow tract; NSVT, non-sustained ventricular tachycardia; NYHA, New York Heart Association; SBP, systolic blood pressure; SCD, sudden cardiac death.

### SP genes variants

Overall, 383 patients (43.8%) had 265 distinct rare (minor allele frequency ≤0.2%) variants in one or more the eight SP genes most commonly associated with HCM *(MYH7, MYBPC3, TNNT2, TNNI3, MYL2, MYL3, ACTC1* and *TPM1*) (table 2 and see online supplementary table S1)*.* A total of 142 (53.5%) of these rare variants were published previously as disease-causing mutations; 44 (16.6%) were novel missense variants predicted in silico to be pathogenic and 40 (15%) were novel potential loss-of-function variants. In all, 37 patients (4.2%) carried multiple candidate variants in these eight SP genes.

**Table 2:** Prevalence of rare variants (minor allele frequency ≤0.2%) in the eight main sarcomere genes

Gene	Number of cases	Percentage of sarcomere-positive individuals (N=383)	Percentage of the total cohort (N=874)
*ACTC1*	3	0.8	0.3
*MYBPC3*	191	49.9	21.9
*MYH7*	99	25.9	11.3
*MYL2*	6	1.6	0.7
*MYL3*	4	1.0	0.5
*TNNI3*	15	3.9	1.7
*TNNT2*	20	5.2	2.3
*TPM1*	8	2.1	0.9
Multiple	37	9.7	4.2
*Total*	383	100	43.8

The number and proportion of individuals for each individual gene excludes patients carrying more than one variant who are grouped under ‘multiple’.

### Non-SP gene variants

In all, 114 distinct rare desmosomal protein gene variants were present in 122 (14.0%) patients; 192 rare ion-channel disease gene variants were present in 196 patients (22.4%). A total of 29 (25.4%) of the desmosomal variants and 38 (19.8%) of the ion-channel variants were published previously. A further 74 (24.2%) of these non-sarcomere variants were novel missense variants predicted in silico to be pathogenic and 20 (6.5%) were potential loss-of-function variants. In all, 122 patients (43.0% of 284) with these non-SP variants also carried a SP variant.

### Genotype–phenotype associations

**Figure 1:**
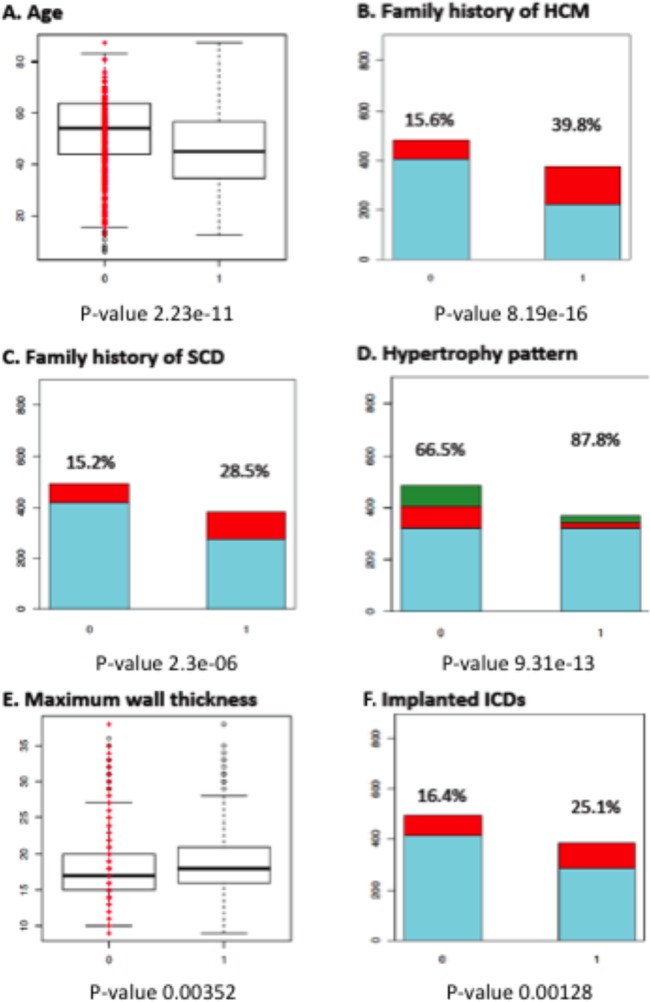
Comparison between sarcomere gene mutation-positive and -negative patients. (A) Age at initial evaluation (45.78±14.65 vs 53.05±14.94 years). (B) Family history of hypertrophic cardiomyopathy (HCM). (C) Family history of sudden cardiac death (SCD). (D) Hypertrophy pattern. (E) Maximum wall thickness (18.83±4.42 vs 18.12±4.08 mm). (F) Implanted implantable cardioverter-defibrillators (ICDs). Key: 0: sarcomere-negative; 1: sarcomere-positive. For B, C, E: red colour and percentages indicate the individuals with the trait within each genotype; for D light blue—asymmetric septal hypertrophy; red—apical hypertrophy; green—concentric hypertrophy.

**Figure 2:**
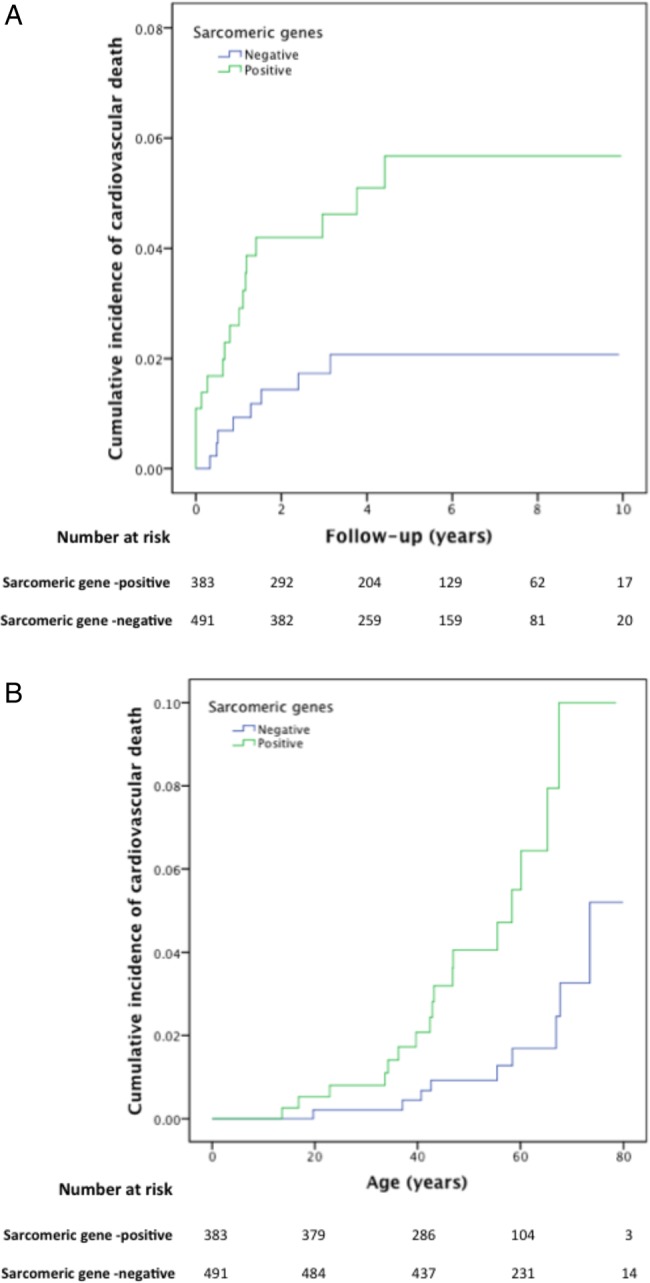
Kaplan–Meier cumulative incidence curves for cardiovascular death (see Methods section), comparing sarcomere-positive and sarcomere-negative individuals, modelled for (A): follow-up from first evaluation (years), log-rank test p value=0.012 (HR 2.81; 95% CI 1.21 to 6.51) and (B): time from birth (years), log-rank test p value=0.001 (HR 3.99; 95% CI 1.71 to 9.36). The Y axis values indicate proportions.

**Figure 3:**
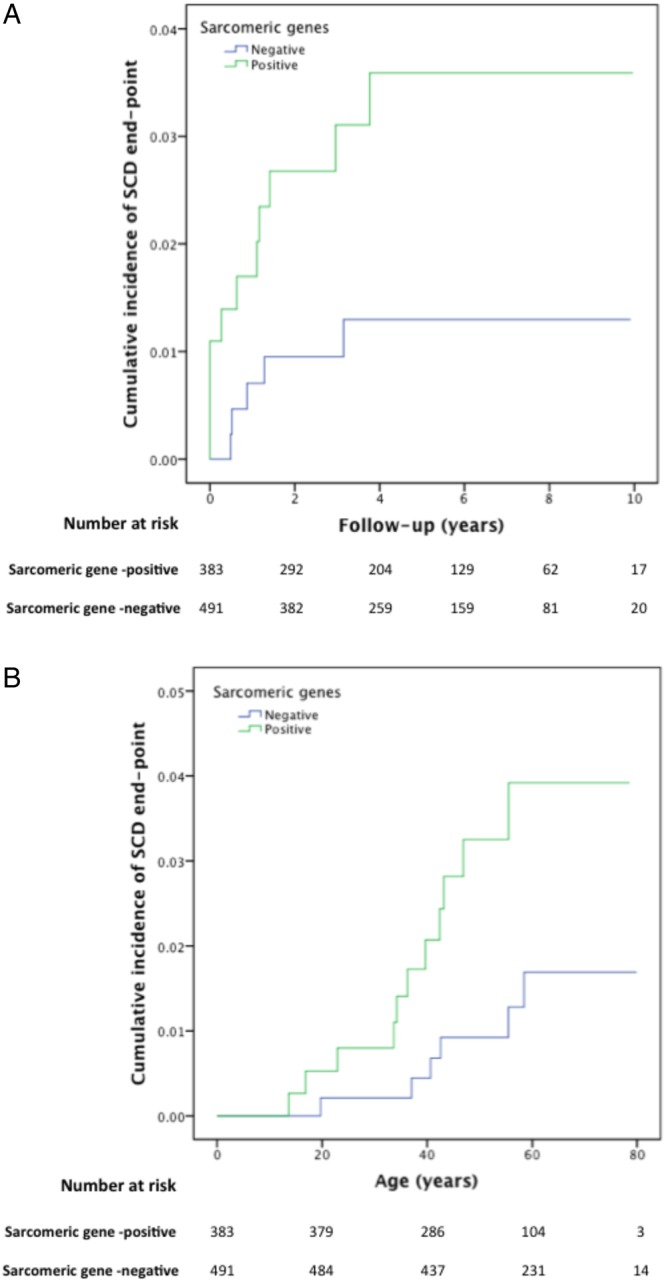
Kaplan–Meier cumulative incidence curves for sudden cardiac death/aborted sudden cardiac death, comparing sarcomere-positive and sarcomere-negative individuals, modelled for (A): follow-up from first evaluation (years), log-rank test p value=0.039 (HR 2.89; 95% CI 1.01 to 8.33) and (B): time from birth (years), log-rank test p value=0.028 (HR 3.44; 95% CI 1.19 to 9.92) . The Y axis values indicate proportions.

Genotype–phenotype associations significant at the defined stringent thresholds are summarised in figures 1–3 and table 3. A complete list of p values significant at p<0.05 for all pairs of traits/genes is provided in table 4 for non-SP genes and online supplementary table S2 for SP and related genes. Online supplementary table S3 summarises the associations for non-SP genes presented in tables 3 and 4, analysed within the subcohort of sarcomere-positive individuals only.

**Table 3:** Genotype–phenotype associations for individual sarcomeric and related protein genes and non-sarcomere protein (SP) genes meeting the predefined statistical thresholds for multiple testing.

Phenotype	Gene	Frequency or mean±SD Rare variant present	Frequency or mean±SD Rare variant absent	p Value
Age at initial evaluation (years)	*MYBPC3*	45.5±14.4	51.0±15.5	8.97×10^−6^
	*MYH7*	43.9±15.4	50.5±15.2	1.91×10^−5^
Family history of HCM	*MYBPC3*	40.4% (86/213)	21.9% (140/640)	2.7×10^−7^
	*MYH7*	47.4% (54/114)	23.4% (173/740)	3.06×10^−7^
Family history of SCD	*MYBPC3*	28.7% (62/216)	18.3% (120/656)	0.001
	*MYH7*	31.6% (37/117)	19.2% (145/775)	0.003
ASH pattern	*MYBPC3*	88.0% (184/209)	71.6% (459/641)	3.75×10^−6^
	*MYH7*	89.2% (99/111)	73.6% (544/739)	0.001
MLVWT (mm)	*MYBPC3*	19.4±4.7	18.2±4.2	0.0005
MLVWT ≥30 mm	*ANK2*	12.5% (6/48)	2% (11/553)	0.0005
LV end-diastolic diameter (mm)	*MYBPC3*	44.8±5.5	46.3±6.0	0.00089
LV end-systolic diameter (mm)	*MYBPC3*	27.4±6.0	28.8±5.4	0.005
Right ventricular hypertrophy	*TNNI3*	50% (10/20)	21.6% (174/806)	0.004
SBP response to exercise (mmHg)	*MYH7*	36.6±19.9	50.2±24.4	5.49×10^−6^
Abnormal SBP response to exercise	*TNNT2*	40.9% (9/22)	13.0% (83/640)	0.002
Myectomy and/or alcohol septal ablation and/or pacemaker implantation for gradient reduction	*MYBPC3* (splicing variants)	43.1% (22/51)	20.0% (160/799)	3.0×10^−4^
LVOTO (>30 mm Hg)	*TNNI3*	10.0% (2/20)	41.2% (326/792)	0.005

p Values reflect the comparison for proportions or means between the group of patients with versus the group of patients without a rare variant in a given gene (p value thresholds of <0.0056 for SP genes and <0.0018 for non-SP genes).

ASH, asymmetric septal hypertrophy; SBP, systolic blood pressure; HCM, hypertrophic cardiomyopathy; LVOTO, LV outflow tract obstruction; MLVWT, maximum LV wall thickness; SCD, sudden cardiac death.

**Table 4:** Genotype–phenotype associations for non-sarcomeric protein genes not meeting the predefined statistical thresholds for multiple testing

Phenotype	Gene	Frequency or mean±SD—Variant present	Frequency or mean±SD—Variant absent	p Value
LA diameter at last follow-up (mm)	*SCN5A*	46.9±5.4	44.3±7.5	0.04
LVOTO (>30 mm Hg)	*SCN5A*	57.5% (23/40)	39.5% (305/772)	0.03
	Ion-channel	49.2% (89/181)	37.9% (239/631)	0.006
MLVWT (mm)	*ANK2*	19.7±5.6	18.4±4.2	0.02
E/e′ ratio	*CASQ2*	16.4±7.1	11.4±5.9	0.02
NSVT	*PLN*	100% (3/3)	22.1% (124/562)	0.01

p Values reflect the comparison for proportions or means between patients with and without a rare variant in a given gene.

E to é ratio, ratio between the maximal velocity of the E wave from the pulsed wave Doppler of the transmitral flow and the maximal velocity of the e′ wave of tissue Doppler at the mitral annulus; LA, left atria; LVOTO, LV outflow tract obstruction; MLVWT, maximal LV wall thickness; NSVT, non-sustained ventricular tachycardia.

### Effect of mutations in sarcomere genes

Patients with at least one variant in one of the eight main sarcomere genes were younger at diagnosis and had a higher frequency of a family history of HCM or SCD compared with those without sarcomere variants. Patients with SP mutations were more likely to have asymmetric septal hypertrophy than apical or concentric patterns and had greater MLVWT. The prevalence of male sex was lower in sarcomere-positive individuals (62.4% vs 72.0%, p=0.00213); these individuals were also more likely to have an ICD implanted. Patients with sarcomere mutations had a lower resting systolic blood pressure (SBP) (123.1±19.2 vs 133.7±21.3 mm Hg, p=1.54×10^−9^) and a lower SBP response to exercise (44.1±21.5 vs 52.2±26.9 mm Hg, p=7.61×10^−5^).

Similar and additional associations were observed when individual SP genes were considered (table 3 and see online supplementary table S2).

The proportion of cardiovascular deaths during follow-up was higher in patients with at least one variant in one of the eight main SP genes. The same was true for sudden death/ICD discharge (figure 2 and 3).

### Patients with multiple SP gene variants

Patients who carried more than one sarcomere variant had an increased prevalence of syncope when compared with individuals with only one sarcomere variant (35.1% vs 16.6%; 13/37 vs 56/337, p=0.012). SBP response to exercise was lower in individuals with multiple sarcomere variants compared with a single variant (36.5±21.9 vs 45.1±21.2 mm Hg, p=0.012) and there was a higher proportion of patients with an abnormal blood pressure response to exercise (10/29 vs 39/276; 34.5% vs 14.1%, p=0.010).

### Associations with rare variants in desmosomal and ion-channel genes

A total of 71 patients carried rare *ANK2* variants (of these, 36 also carried SP variants). At a significance threshold of p<0.0018, the proportion of patients with an MLVWT ≥30 mm was greater in carriers of an *ANK2* rare variant (table 3). This association was still present when restraining the analyses to the subcohort of sarcomere-positive individuals only (see online supplementary table S3).

Additional genotype–phenotype correlations were identified at a less stringent p<0.05. These are listed in table 4, and include an increased mean MLVWT in *ANK2* variant carriers.

## Discussion

In this study of a large consecutive cohort of HCM probands screened with high-throughput sequencing, we have detected a class effect of SP gene variants on the HCM phenotype and identified novel associations with mutations in individual SP genes. We also demonstrated evidence of an association between non-SP genes and disease expression that could explain some of the characteristic clinical heterogeneity of HCM.

### Influence of sarcomeric variation on phenotype

The presence of any sarcomere variant was associated with an asymmetric septal hypertrophy pattern, younger age at presentation, family history of HCM and SCD and female gender. This study also shows that patients with SP variants had higher cardiovascular and sudden death-related mortality during follow-up. Patients with more than one SP variant had more SCD risk markers, consistent with the suggestion in previously published series of a gene dose effect.^13^ ^26–28^ However, the low number of outcome events during follow-up may have biased the survival analysis and precluded an analysis of other associations, including the effect of carrying multiple compared with single variants. The survival from birth is provided for comparison with the published literature but also introduces an inherent survivor bias. With regard to individual SP genes, we demonstrate a number of novel associations that provide evidence for mutation specific effects on clinical phenotype and prognosis.

### Modifier effect of non-sarcomere variants

The data in this study suggest that rare *ANK2* variants are associated with severe LV hypertrophy. *ANK2*, or *ankyrin B*, stabilises membrane ion-channels in cardiomyocytes and mutations in the gene cause long QT syndrome 4, ventricular arrhythmias and sinus node disease.^29^ ^30^ We are unaware of any link between *ANK2* expression and changes in LV morphology, but as ankyrins interact with proteins that influence calcium homeostasis and ß-adrenergic signalling, it is conceivable that they eventually affect the cellular phenotype that results from a primary SP gene variant. The strength of the statistical association (p=0.0005) exceeds the requirement of a Bonferroni correction for the number of tested genes (36 independent tests), but further replication in independent cohorts will be necessary to confirm these results.

In addition to the association with *ANK2* variation, we detected a number of associations at lower statistical significance with variation in other non-SP genes. Patients with *SCN5A* rare variants were more likely to have left atria enlargement at their last evaluation. A link between *SCN5A* disruption and TGF-β_1_-mediated fibrosis has recently been demonstrated in a murine model of sinus node disease^w28^ and it is possible that *SCN5A* variants influence the pro-fibrotic milieu associated with SP mutations. SCN5A rare variation was also associated with a higher proportion of LV outflow tract obstruction. Individuals with *PLN* rare variants were more likely to have NSVT, which is interesting considering the recently described arrhythmogenic risk of a founder *PLN* mutation.^w29^ As for the association with *ANK2*, replication of these findings is required.

### Clinical implications

If genetic variation is to become a clinically relevant biomarker, it is essential that there is a clear understanding of genotype–phenotype relationships. The associations between sarcomere gene variants and the broad phenotype examined in this study contribute to this understanding and, if confirmed in other populations, could inform the counselling of patients and relatives who are contemplating predictive genetic testing. The demonstration that non-sarcomeric variants may influence disease expression is an illustration of the complexity that underlies the biology of this disease. New models that incorporate a broad genetic profile and deep clinical phenotyping are necessary to test the role of mutation analysis in prognostic models.

**Key messages**

- **What is known on this subject?**
- In up to 50% of cases, hypertrophic cardiomyopathy is caused by mutations in genes coding for sarcomere or sarcomere-related genes, but the often dramatic variation in clinical phenotypes caused by the same or similar mutations remains largely unexplained.

**What might this study add?**

- This study presents novel genotype–phenotype associations in a large cohort of 874 patients using high-throughput genetic sequencing. We describe a strong class effect for sarcomeric protein variants on clinical presentation, LV morphology and survival. For the first time, a modifier effect of rare variants in non-sarcomeric genes associated with other forms of cardiomyopathy and arrhythmia syndromes is demonstrated.

**How might this impact on clinical practice?**

- These are novel findings which suggest new and testable insights on the biology and pathophysiology of the disease that might eventually have important clinical implications for counselling of patients and risk prediction models.

## Supplementary Material

Web supplement

Web supplement

Web supplement

Web supplement
