# Supplementary material for: Novel genotype–phenotype associations demonstrated by high-throughput sequencing in patients with hypertrophic cardiomyopathy
Source: Heart. 2014 Oct 28;101(4):294–301. doi: 10.1136/heartjnl-2014-306387 (PMC4345808; doi:10.1136/heartjnl-2014-306387)
Supplement: Web supplement [file heartjnl-2014-306387-s1.pdf]

Online references

w1. Epstein ND, Cohn GM, Cyran F, et al. Differences in clinical expression of hypertrophic cardiomyopathy associated with two distinct mutations in the beta-myosin heavy chain gene. A 908Leu----Val mutation and a 403Arg----Gln mutation. *Circulation* 1992;**86**:345-52.
w2. Fananapazir L, Epstein ND. Genotype-phenotype correlations in hypertrophic cardiomyopathy. Insights provided by comparisons of kindreds with distinct and identical beta-myosin heavy chain gene mutations. *Circulation* 1994;**89**:22-32.
w3. Watkins H, Rosenzweig A, Hwang DS, et al. Characteristics and prognostic implications of myosin missense mutations in familial hypertrophic cardiomyopathy. *N Engl J Med* 1992;**326**:1108-14.
w4. Anan R, Greve G, Thierfelder L, et al. Prognostic implications of novel beta cardiac myosin heavy chain gene mutations that cause familial hypertrophic cardiomyopathy. *J Clin Invest* 1994;**93**:280-5.
w5. Watkins H, McKenna WJ, Thierfelder L, et al. Mutations in the genes for cardiac troponin T and alpha-tropomyosin in hypertrophic cardiomyopathy. *N Engl J Med* 1995;**332**:1058-64.
w6. Tesson F, Richard P, Charron P, et al. Genotype-phenotype analysis in four families with mutations in beta-myosin heavy chain gene responsible for familial hypertrophic cardiomyopathy. *Hum Mutat* 1998;**12**:385-92.
w7. Coviello DA, Maron BJ, Spirito P, et al. Clinical features of hypertrophic cardiomyopathy caused by mutation of a "hot spot" in the alpha-tropomyosin gene. *J Am Coll Cardiol* 1997;**29**:635-40.
w8. Anan R, Shono H, Kisanuki A, et al. Patients with familial hypertrophic cardiomyopathy caused by a Phe110Ile missense mutation in the cardiac troponin T gene have variable cardiac morphologies and a favorable prognosis. *Circulation* 1998;**98**:391-7.
w9. Marian AJ, Mares A, Jr., Kelly DP, et al. Sudden cardiac death in hypertrophic cardiomyopathy. Variability in phenotypic expression of beta-myosin heavy chain mutations. *Eur Heart J* 1995;**16**:368-76.
w10. Kokado H, Shimizu M, Yoshio H, et al. Clinical features of hypertrophic cardiomyopathy caused by a Lys183 deletion mutation in the cardiac troponin I gene. *Circulation* 2000;**102**:663-9.
w11. Moolman JA, Reith S, Uhl K, et al. A newly created splice donor site in exon 25 of the MyBP-C gene is responsible for inherited hypertrophic cardiomyopathy with incomplete disease penetrance. *Circulation* 2000;**101**:1396-402.
w12. Niimura H, Bachinski LL, Sangwatanaroj S, et al. Mutations in the gene for cardiac myosin-binding protein C and late-onset familial hypertrophic cardiomyopathy. *N Engl J Med* 1998;**338**:1248-57.
w13. Maron BJ, Niimura H, Casey SA, et al. Development of left ventricular hypertrophy in adults in hypertrophic cardiomyopathy caused by cardiac myosin-binding protein C gene mutations. *J Am Coll Cardiol* 2001;**38**:315-21.
w14. Niimura H, Patton KK, McKenna WJ, et al. Sarcomere protein gene mutations in hypertrophic cardiomyopathy of the elderly. *Circulation* 2002;**105**:446-51.
w15. Doi YL, Kitaoka H, Hitomi N, et al. Clinical expression in patients with hypertrophic cardiomyopathy caused by cardiac myosin-binding protein C gene mutation. *Circulation* 1999;**100**:448-9.
w16. Konno T, Shimizu M, Ino H, et al. A novel missense mutation in the myosin binding protein-C gene is responsible for hypertrophic cardiomyopathy with left ventricular dysfunction and dilation in elderly patients. *J Am Coll Cardiol* 2003;**41**:781-6.
w17. Erdmann J, Raible J, Maki-Abadi J, et al. Spectrum of clinical phenotypes and gene variants in cardiac myosin-binding protein C mutation carriers with hypertrophic cardiomyopathy. *J Am Coll Cardiol* 2001;**38**:322-30.

w18.Brito D, Richard P, Isnard R, et al. Familial hypertrophic cardiomyopathy: the same mutation, different prognosis. Comparison of two families with a long follow-up. *Rev Port Cardiol* 2003;**22**:1445-61.
w19.Brito D, Richard P, Komajda M, et al. Familial and sporadic hypertrophic myopathy: differences and similarities in a genotyped population. A long follow-up study. *Rev Port Cardiol* 2008;**27**:147-73.
w20.Osterop AP, Kofflard MJ, Sandkuijl LA, et al. AT1 receptor A/C1166 polymorphism contributes to cardiac hypertrophy in subjects with hypertrophic cardiomyopathy. *Hypertension* 1998;**32**:825-30.
w21.Kaufman BD, Auerbach S, Reddy S, et al. RAAS gene polymorphisms influence progression of pediatric hypertrophic cardiomyopathy. *Hum Genet* 2007;**122**:515-23.
w22.Funada A, Konno T, Fujino N, et al. Impact of renin-angiotensin system polymorphisms on development of systolic dysfunction in hypertrophic cardiomyopathy. Evidence from a study of genotyped patients. *Circ J* 2010;**74**:2674-80.
w23.Wang K, Li M, Hakonarson H. ANNOVAR: functional annotation of genetic variants from high-throughput sequencing data. *Nucleic Acids Res* 2010;**38**:e164.
w24.Pearson RD. Bias due to selection of rare variants using frequency in controls. *Nat Genet* 2011;**43**:392-3.
w25.Smigielski EM, Sirotkin K, Ward M, et al. dbSNP: a database of single nucleotide polymorphisms. *Nucleic Acids Res* 2000;**28**:352-5.
w26.Ng PC, Henikoff S. SIFT: Predicting amino acid changes that affect protein function. *Nucleic Acids Res* 2003;**31**:3812-4.
w27.Adzhubei IA, Schmidt S, Peshkin L, et al. A method and server for predicting damaging missense mutations. *Nat Methods* 2010;**7**:248-9.
w28.Hao X, Zhang Y, Zhang X, et al. TGF-beta1-mediated fibrosis and ion channel remodeling are key mechanisms in producing the sinus node dysfunction associated with SCN5A deficiency and aging. *Circ Arrhythm Electrophysiol* 2011;**4**:397-406.
w29.van der Zwaag PA, van Rijsingen IA, de Ruiter R, et al. Recurrent and founder mutations in the Netherlands-Phospholamban p.Arg14del mutation causes arrhythmogenic cardiomyopathy. *Neth Heart J* 2013;**21**:286-93.
