# Supplementary material for: Novel genotype–phenotype associations demonstrated by high-throughput sequencing in patients with hypertrophic cardiomyopathy
Source: Heart. 2014 Oct 28;101(4):294–301. doi: 10.1136/heartjnl-2014-306387 (PMC4345808; doi:10.1136/heartjnl-2014-306387)
Supplement: Web supplement [file heartjnl-2014-306387-s2.pdf]

Supplementary table 1. List of distinct rare variants (MAF<0.2%; see methods). All variants were detected in heterozygozity. Type of variant: D: variant reported on dbSNP137; L: novel predicted loss-of-function (nonsense, frameshift or splice site) variant; N: novel non-frameshift or missense variant not predicted *in silico* to be pathogenic; P: variant reported in the literature as disease-associated; S: novel missense variant predicted *in silico* to be pathogenic. MAF: minor allele frequency.

**Gene**	**Genomic signature**	**Transcript / Amino acid change**	**dbSNP137 ID**	**Type of variant**	**MAF- cases**
ACTC1	15_35084398_G_A	ENSG00000159251:ENST00000290378:exon5:c.C701T:p.S234F		N	0,00057
ACTC1	15_35085539_T_C	ENSG00000159251:ENST00000290378:exon3:c.A361G:p.M121V		S	0,00058
ACTC1	15_35085599_C_T	ENSG00000159251:ENST00000290378:exon3:c.G301A:p.E101K	rs193922680	D;P	0,00057
ANK2	4_113970940_G_A	ENSG00000145362:ENST00000264366:exon1:c.G56A:p.S19N,ENSG00000145362:ENST00000357077:exon1:c.G56A:p.S19N,ENSG00000145362:ENST00000394537:exon1:c.G56A:p.S19N,ENSG00000145362:ENST00000504454:exon1:c.G56A:p.S19N		N	0,00057
ANK2	4_114067089_T_A	ENSG00000145362:ENST00000511380:exon1:c.T65A:p.M22K	rs116338686	D	0,00115
ANK2	4_114072285_T_C	ENST00000504454:exon2:c.129+5T>C		L	0,00057
ANK2	4_114158192_C_T	ENSG00000145362:ENST00000264366:exon6:c.C533T:p.A178V,ENSG00000145362:ENST00000357077:exon6:c.C533T:p.A178V,ENSG00000145362:ENST00000394537:exon6:c.C533T:p.A178V,ENSG00000145362:ENST00000515034:exon6:c.C128T:p.A43V,ENSG00000145362:ENST00000503271:exon7:c.C470T:p.A157V,ENSG00000145362:ENST00000503423:exon7:c.C470T:p.A157V,ENSG00000145362:ENST00000504454:exon7:c.C578T:p.A193V,ENSG00000145362:ENST00000506722:exon7:c.C470T:p.A157V		N	0,00058
ANK2	4_114195614_C_G	ENSG00000145362:ENST00000264366:exon15:c.C1492G:p.Q498E,ENSG00000145362:ENST00000357077:exon15:c.C1492G:p.Q498E,ENSG00000145362:ENST00000394537:exon15:c.C1492G:p.Q498E,ENSG00000145362:ENST00000503271:exon16:c.C1429G:p.Q477E,ENSG00000145362:ENST00000503423:exon16:c.C1429G:p.Q477E,ENSG00000145362:ENST00000504454:exon16:c.C1537G:p.Q513E,ENSG00000145362:ENST00000506722:exon16:c.C1429G:p.Q477E		S	0,00057
ANK2	4_114199026_T_C	ENSG00000145362:ENST00000264366:exon16:c.T1717C:p.Y573H,ENSG00000145362:ENST00000357077:exon16:c.T1717C:p.Y573H,ENSG00000145362:ENST00000394537:exon16:c.T1717C:p.Y573H,ENSG00000145362:ENST00000503271:exon17:c.T1654C:p.Y552H,ENSG00000145362:ENST00000503423:exon17:c.T1654C:p.Y552H,ENSG00000145362:ENST00000504454:exon17:c.T1762C:p.Y588H,ENSG00000145362:ENST00000506722:exon17:c.T1654C:p.Y552H		S	0,00057

ANK2	4_114208841_T_A	ENSG00000145362:ENST00000264366:exon19:c.T2160A:p.D720E,ENSG00000145362:ENST00000357077:exon19:c.T2160A:p.D720E,ENSG00000145362:ENST00000394537:exon19:c.T2160A:p.D720E,ENSG00000145362:ENST00000503423:exon19:c.T1998A:p.D666E,ENSG00000145362:ENST00000503271:exon20:c.T2097A:p.D699E,ENSG00000145362:ENST00000504454:exon20:c.T2205A:p.D735E,ENSG00000145362:ENST00000506722:exon20:c.T2097A:p.D699E		N	0,00057
ANK2	4_114239695_C_G	ENSG00000145362:ENST00000509550:exon6:c.C446G:p.A149G,ENSG00000145362:ENST00000264366:exon26:c.C2819G:p.A940G,ENSG00000145362:ENST00000357077:exon26:c.C2819G:p.A940G,ENSG00000145362:ENST00000394537:exon26:c.C2819G:p.A940G,ENSG00000145362:ENST00000503423:exon26:c.C2657G:p.A886G,ENSG00000145362:ENST00000503271:exon27:c.C2756G:p.A919G,ENSG00000145362:ENST00000504454:exon27:c.C2864G:p.A955G,ENSG00000145362:ENST00000506722:exon27:c.C2756G:p.A919G		S	0,00115
ANK2	4_114244911_G_A	ENST00000506722:exon28:-4G>A	rs139641776	D; L	0,00057
ANK2	4_114244926_C_A	ENSG00000145362:ENST00000506722:exon28:c.C2849A:p.P950Q		N	0,00114
ANK2	4_114251533_G_A	ENSG00000145362:ENST00000509550:exon7:c.G659A:p.R220H,ENSG00000145362:ENST00000264366:exon27:c.G3032A:p.R1011H,ENSG00000145362:ENST00000357077:exon27:c.G3032A:p.R1011H,ENSG00000145362:ENST00000394537:exon27:c.G3032A:p.R1011H,ENSG00000145362:ENST00000503423:exon27:c.G2870A:p.R957H,ENSG00000145362:ENST00000503271:exon28:c.G2969A:p.R990H,ENSG00000145362:ENST00000504454:exon28:c.G3077A:p.R1026H,ENSG00000145362:ENST00000506722:exon29:c.G3005A:p.R1002H		S	0,00058
ANK2	4_114251575_G_C	ENSG00000145362:ENST00000509550:exon7:c.G701C:p.G234A,ENSG00000145362:ENST00000264366:exon27:c.G3074C:p.G1025A,ENSG00000145362:ENST00000357077:exon27:c.G3074C:p.G1025A,ENSG00000145362:ENST00000394537:exon27:c.G3074C:p.G1025A,ENSG00000145362:ENST00000503423:exon27:c.G2912C:p.G971A,ENSG00000145362:ENST00000503271:exon28:c.G3011C:p.G1004A,ENSG00000145362:ENST00000504454:exon28:c.G3119C:p.G1040A,ENSG00000145362:ENST00000506722:exon29:c.G3047C:p.G1016A		S	0,00058
ANK2	4_114263066_T_G	ENSG00000145362:ENST00000510275:exon1:c.T72G:p.D24E,ENSG00000145362:ENST00000509550:exon12:c.T1644G:p.D548E,ENSG00000145362:ENST00000264366:exon32:c.T4017G:p.D1339E,ENSG00000145362:ENST00000503423:exon32:c.T3855G:p.D1285E,ENSG00000145362:ENST00000357077:exon33:c.T4116G:p.D1372E,ENSG00000145362:ENST00000394537:exon33:c.T4116G:p.D1372E,ENSG00000145362:ENST00000504454:exon34:c.T4161G:p.D1387E,ENSG00000145362:ENST00000506722:exon35:c.T4089G:p.D1363E		S	0,00057

ANK2	4_114267117_C_T	ENSG00000145362:ENST00000510275:exon3:c.C266T:p.T89M,ENSG00000145362:ENST00000509550:exon14:c.C1838T:p.T613M,ENSG00000145362:ENST00000264366:exon34:c.C4211T:p.T1404M,ENSG00000145362:ENST00000503423:exon34:c.C4049T:p.T1350M,ENSG00000145362:ENST00000357077:exon35:c.C4310T:p.T1437M,ENSG00000145362:ENST00000394537:exon35:c.C4310T:p.T1437M,ENSG00000145362:ENST00000504454:exon36:c.C4355T:p.T1452M,ENSG00000145362:ENST00000506722:exon37:c.C4283T:p.T1428M	rs142534126	D;S	0,00057
ANK2	4_114267122_G_T	ENSG00000145362:ENST00000510275:exon3:c.G271T:p.G91C,ENSG00000145362:ENST00000509550:exon14:c.G1843T:p.G615C,ENSG00000145362:ENST00000264366:exon34:c.G4216T:p.G1406C,ENSG00000145362:ENST00000503423:exon34:c.G4054T:p.G1352C,ENSG00000145362:ENST00000357077:exon35:c.G4315T:p.G1439C,ENSG00000145362:ENST00000394537:exon35:c.G4315T:p.G1439C,ENSG00000145362:ENST00000504454:exon36:c.G4360T:p.G1454C,ENSG00000145362:ENST00000506722:exon37:c.G4288T:p.G1430C	rs34591340	D;P	0,00057
ANK2	4_114267176_A_G	ENSG00000145362:ENST00000510275:exon3:c.A325G:p.K109E,ENSG00000145362:ENST00000509550:exon14:c.A1897G:p.K633E,ENSG00000145362:ENST00000264366:exon34:c.A4270G:p.K1424E,ENSG00000145362:ENST00000503423:exon34:c.A4108G:p.K1370E,ENSG00000145362:ENST00000357077:exon35:c.A4369G:p.K1457E,ENSG00000145362:ENST00000394537:exon35:c.A4369G:p.K1457E,ENSG00000145362:ENST00000504454:exon36:c.A4414G:p.K1472E,ENSG00000145362:ENST00000506722:exon37:c.A4342G:p.K1448E		N	0,00057
ANK2	4_114269433_A_G	ENSG00000145362:ENST00000510275:exon4:c.A329G:p.E110G,ENSG00000145362:ENST00000509550:exon15:c.A1901G:p.E634G,ENSG00000145362:ENST00000264366:exon35:c.A4274G:p.E1425G,ENSG00000145362:ENST00000503423:exon35:c.A4112G:p.E1371G,ENSG00000145362:ENST00000357077:exon36:c.A4373G:p.E1458G,ENSG00000145362:ENST00000394537:exon36:c.A4373G:p.E1458G,ENSG00000145362:ENST00000504454:exon37:c.A4418G:p.E1473G,ENSG00000145362:ENST00000506722:exon38:c.A4346G:p.E1449G	rs72544141	D;P	0,00172
ANK2	4_114274519_G_A	ENSG00000145362:ENST00000264366:exon37:c.G4646A:p.R1549Q,ENSG00000145362:ENST00000503423:exon37:c.G4484A:p.R1495Q,ENSG00000145362:ENST00000357077:exon38:c.G4745A:p.R1582Q,ENSG00000145362:ENST00000504454:exon39:c.G4790A:p.R1597Q	rs138842207	D	0,00286
ANK2	4_114274546_A_G	ENSG00000145362:ENST00000264366:exon37:c.A4673G:p.E1558G,ENSG00000145362:ENST00000503423:exon37:c.A4511G:p.E1504G,ENSG00000145362:ENST00000357077:exon38:c.A4772G:p.E1591G,ENSG00000145362:ENST00000504454:exon39:c.A4817G:p.E1606G		S	0,00057
ANK2	4_114274806_G_A	ENSG00000145362:ENST00000264366:exon37:c.G4933A:p.E1645K,ENSG00000145362:ENST00000503423:exon37:c.G4771A:p.E1591K,ENSG00000145362:ENST00000357077:exon38:c.G5032A:p.E1678K,ENSG00000145362:ENST00000504454:exon39:c.G5077A:p.E1693K		N	0,00057
ANK2	4_114275005_C_A	ENSG00000145362:ENST00000264366:exon37:c.C5132A:p.A1711D,ENSG00000145362:ENST00000357077:exon38:c.C5231A:p.A1744D	rs147706514	D	0,00057
ANK2	4_114275005_C_T	ENSG00000145362:ENST00000264366:exon37:c.C5132T:p.A1711V,ENSG00000145362:ENST00000357077:exon38:c.C5231T:p.A1744V		N	0,00057

ANK2	4_114275096_G_T	ENSG00000145362:ENST00000264366:exon37:c.G5223T:p.Q1741H,ENSG00000145362:ENST00000357077:exon38:c.G5322T:p.Q1774H		S	0,00057
ANK2	4_114275262_C_T	ENSG00000145362:ENST00000264366:exon37:c.C5389T:p.H1797Y,ENSG00000145362:ENST00000357077:exon38:c.C5488T:p.H1830Y		N	0,00057
ANK2	4_114275344_T_A	ENSG00000145362:ENST00000264366:exon37:c.T5471A:p.V1824E,ENSG00000145362:ENST00000357077:exon38:c.T5570A:p.V1857E	rs141212932	D	0,00057
ANK2	4_114275425_C_T	ENSG00000145362:ENST00000264366:exon37:c.C5552T:p.S1851L,ENSG00000145362:ENST00000357077:exon38:c.C5651T:p.S1884L	rs150737736	D	0,00057
ANK2	4_114275688_C_A	ENSG00000145362:ENST00000264366:exon37:c.C5815A:p.Q1939K,ENSG00000145362:ENST00000357077:exon38:c.C5914A:p.Q1972K		N	0,00057
ANK2	4_114275852_A_T	ENSG00000145362:ENST00000264366:exon37:c.A5979T:p.K1993N,ENSG00000145362:ENST00000357077:exon38:c.A6078T:p.K2026N		N	0,00115
ANK2	4_114275950_C_T	ENSG00000145362:ENST00000264366:exon37:c.C6077T:p.T2026M,ENSG00000145362:ENST00000357077:exon38:c.C6176T:p.T2059M	rs200765866	D;S	0,00058
ANK2	4_114275980_G_A	ENSG00000145362:ENST00000264366:exon37:c.G6107A:p.R2036H,ENSG00000145362:ENST00000357077:exon38:c.G6206A:p.R2069H	rs149645600	D;S	0,00058
ANK2	4_114276279_G_A	ENSG00000145362:ENST00000264366:exon37:c.G6406A:p.V2136I,ENSG00000145362:ENST00000357077:exon38:c.G6505A:p.V2169I	rs149292242	D;S	0,00057
ANK2	4_114276360_G_A	ENSG00000145362:ENST00000264366:exon37:c.G6487A:p.G2163S,ENSG00000145362:ENST00000357077:exon38:c.G6586A:p.G2196S		N	0,00057
ANK2	4_114276657_A_G	ENSG00000145362:ENST00000264366:exon37:c.A6784G:p.T2262A,ENSG00000145362:ENST00000357077:exon38:c.A6883G:p.T2295A		N	0,00058
ANK2	4_114276879_G_T	ENSG00000145362:ENST00000264366:exon37:c.G7006T:p.V2336F,ENSG00000145362:ENST00000357077:exon38:c.G7105T:p.V2369F		N	0,00130
ANK2	4_114276891_A_G	ENSG00000145362:ENST00000264366:exon37:c.A7018G:p.T2340A,ENSG00000145362:ENST00000357077:exon38:c.A7117G:p.T2373A	rs184514058	D	0,00066
ANK2	4_114276922_C_T	ENSG00000145362:ENST00000264366:exon37:c.C7049T:p.P2350L,ENSG00000145362:ENST00000357077:exon38:c.C7148T:p.P2383L	rs35960628	D	0,00066
ANK2	4_114276957_A_C	ENSG00000145362:ENST00000264366:exon37:c.A7084C:p.T2362P,ENSG00000145362:ENST00000357077:exon38:c.A7183C:p.T2395P	rs201693280	D	0,00133
ANK2	4_114277041_G_A	ENSG00000145362:ENST00000264366:exon37:c.G7168A:p.A2390T,ENSG00000145362:ENST00000357077:exon38:c.G7267A:p.A2423T	rs3733616	D	0,00062
ANK2	4_114277108_A_G	ENSG00000145362:ENST00000264366:exon37:c.A7235G:p.D2412G,ENSG00000145362:ENST00000357077:exon38:c.A7334G:p.D2445G		S	0,00059
ANK2	4_114277642_C_G	ENSG00000145362:ENST00000264366:exon37:c.C7769G:p.S2590C,ENSG00000145362:ENST00000357077:exon38:c.C7868G:p.S2623C	rs116253689	D	0,00057

ANK2	4_114277871_G_A	ENSG00000145362:ENST00000264366:exon37:c.G7998A:p.M2666I,ENSG00000145362:ENST00000357077:exon38:c.G8097A:p.M2699I	rs148904454	D	0,00058
ANK2	4_114278014_G_A	ENSG00000145362:ENST00000264366:exon37:c.G8141A:p.R2714H,ENSG00000145362:ENST00000357077:exon38:c.G8240A:p.R2747H	rs142137451	D	0,00057
ANK2	4_114278542_A_G	ENSG00000145362:ENST00000264366:exon37:c.A8669G:p.Q2890R,ENSG00000145362:ENST00000357077:exon38:c.A8768G:p.Q2923R		S	0,00057
ANK2	4_114278701_C_G	ENSG00000145362:ENST00000264366:exon37:c.C8828G:p.S2943C,ENSG00000145362:ENST00000357077:exon38:c.C8927G:p.S2976C		S	0,00057
ANK2	4_114278820_G_A	ENSG00000145362:ENST00000505342:exon1:c.G76A:p.E26K,ENSG00000145362:ENST00000264366:exon37:c.G8947A:p.E2983K,ENSG00000145362:ENST00000357077:exon38:c.G9046A:p.E3016K	rs149963885	D	0,00057
ANK2	4_114279054_C_G	ENSG00000145362:ENST00000505342:exon1:c.C310G:p.P104A,ENSG00000145362:ENST00000264366:exon37:c.C9181G:p.P3061A,ENSG00000145362:ENST00000357077:exon38:c.C9280G:p.P3094A		S	0,00115
ANK2	4_114279615_C_G	ENSG00000145362:ENST00000505342:exon1:c.C871G:p.Q291E,ENSG00000145362:ENST00000264366:exon37:c.C9742G:p.Q3248E,ENSG00000145362:ENST00000357077:exon38:c.C9841G:p.Q3281E		N	0,00057
ANK2	4_114279646_C_G	ENSG00000145362:ENST00000505342:exon1:c.C902G:p.P301R,ENSG00000145362:ENST00000264366:exon37:c.C9773G:p.P3258R,ENSG00000145362:ENST00000357077:exon38:c.C9872G:p.P3291R		N	0,00057
ANK2	4_114279892_C_T	ENSG00000145362:ENST00000505342:exon1:c.C1148T:p.A383V,ENSG00000145362:ENST00000264366:exon37:c.C10019T:p.A3340V,ENSG00000145362:ENST00000357077:exon38:c.C10118T:p.A3373V		N	0,00059
ANK2	4_114280441_A_G	ENSG00000145362:ENST00000505342:exon1:c.A1697G:p.H566R,ENSG00000145362:ENST00000264366:exon37:c.A10568G:p.H3523R,ENSG00000145362:ENST00000357077:exon38:c.A10667G:p.H3556R		S	0,00057
ANK2	4_114281999_C_T	ENSG00000145362:ENST00000505342:exon2:c.C1732T:p.R578W,ENSG00000145362:ENST00000510275:exon6:c.C403T:p.R135W,ENSG00000145362:ENST00000509550:exon17:c.C1975T:p.R659W,ENSG00000145362:ENST00000264366:exon38:c.C10603T:p.R3535W,ENSG00000145362:ENST00000394537:exon38:c.C4447T:p.R1483W,ENSG00000145362:ENST00000357077:exon39:c.C10702T:p.R3568W,ENSG00000145362:ENST00000506722:exon40:c.C4420T:p.R1474W	rs72556376	D;P	0,00057
ANK2	4_114282005_G_A	ENSG00000145362:ENST00000505342:exon2:c.G1738A:p.E580K,ENSG00000145362:ENST00000510275:exon6:c.G409A:p.E137K,ENSG00000145362:ENST00000509550:exon17:c.G1981A:p.E661K,ENSG00000145362:ENST00000264366:exon38:c.G10609A:p.E3537K,ENSG00000145362:ENST00000394537:exon38:c.G4453A:p.E1485K,ENSG00000145362:ENST00000357077:exon39:c.G10708A:p.E3570K,ENSG00000145362:ENST00000506722:exon40:c.G4426A:p.E1476K	rs180843436	D;P	0,00057

ANK2	4_114290677_A_G	ENSG00000145362:ENST00000505342:exon6:c.A2356G:p.T786A,ENSG00000145362:ENST00000510275:exon10:c.A1027G:p.T343A,ENSG00000145362:ENST00000509550:exon21:c.A2599G:p.T867A,ENSG00000145362:ENST00000264366:exon42:c.A11227G:p.T3743A,ENSG00000145362:ENST00000394537:exon42:c.A5071G:p.T1691A,ENSG00000145362:ENST00000357077:exon43:c.A11326G:p.T3776A,ENSG00000145362:ENST00000506722:exon44:c.A5044G:p.T1682A		S	0,00057
ANK2	4_114290837_C_T	ENSG00000145362:ENST00000505342:exon6:c.C2516T:p.P839L,ENSG00000145362:ENST00000510275:exon10:c.C1187T:p.P396L,ENSG00000145362:ENST00000509550:exon21:c.C2759T:p.P920L,ENSG00000145362:ENST00000264366:exon42:c.C11387T:p.P3796L,ENSG00000145362:ENST00000394537:exon42:c.C5231T:p.P1744L,ENSG00000145362:ENST00000357077:exon43:c.C11486T:p.P3829L,ENSG00000145362:ENST00000506722:exon44:c.C5204T:p.P1735L		N	0,00058
ANK2	4_114290844_G_T	ENSG00000145362:ENST00000505342:exon6:c.G2523T:p.E841D,ENSG00000145362:ENST00000510275:exon10:c.G1194T:p.E398D,ENSG00000145362:ENST00000509550:exon21:c.G2766T:p.E922D,ENSG00000145362:ENST00000264366:exon42:c.G11394T:p.E3798D,ENSG00000145362:ENST00000394537:exon42:c.G5238T:p.E1746D,ENSG00000145362:ENST00000357077:exon43:c.G11493T:p.E3831D,ENSG00000145362:ENST00000506722:exon44:c.G5211T:p.E1737D	rs144046572	D;S	0,00058
ANK2	4_114290875_C_T	ENSG00000145362:ENST00000505342:exon6:c.C2554T:p.R852W,ENSG00000145362:ENST00000510275:exon10:c.C1225T:p.R409W,ENSG00000145362:ENST00000509550:exon21:c.C2797T:p.R933W,ENSG00000145362:ENST00000264366:exon42:c.C11425T:p.R3809W,ENSG00000145362:ENST00000394537:exon42:c.C5269T:p.R1757W,ENSG00000145362:ENST00000357077:exon43:c.C11524T:p.R3842W,ENSG00000145362:ENST00000506722:exon44:c.C5242T:p.R1748W	rs139797180	D;S	0,00057
ANK2	4_114293765_G_A	ENSG00000145362:ENST00000510275:exon11:c.G1388A:p.R463Q		N	0,00057
ANK2	4_114294276_A_G	ENSG00000145362:ENST00000505342:exon7:c.A2671G:p.T891A,ENSG00000145362:ENST00000510275:exon12:c.A1435G:p.T479A,ENSG00000145362:ENST00000509550:exon22:c.A2914G:p.T972A,ENSG00000145362:ENST00000264366:exon43:c.A11542G:p.T3848A,ENSG00000145362:ENST00000394537:exon43:c.A5386G:p.T1796A,ENSG00000145362:ENST00000357077:exon44:c.A11641G:p.T3881A,ENSG00000145362:ENST00000506722:exon45:c.A5359G:p.T1787A		N	0,00057
ANK2	4_114294462_C_T	ENSG00000145362:ENST00000505342:exon8:c.C2746T:p.R916W,ENSG00000145362:ENST00000510275:exon13:c.C1510T:p.R504W,ENSG00000145362:ENST00000509550:exon23:c.C2989T:p.R997W,ENSG00000145362:ENST00000264366:exon44:c.C11617T:p.R3873W,ENSG00000145362:ENST00000394537:exon44:c.C5461T:p.R1821W,ENSG00000145362:ENST00000357077:exon45:c.C11716T:p.R3906W,ENSG00000145362:ENST00000506722:exon46:c.C5434T:p.R1812W	rs121912706	D;P	0,00172
ANK2	4_114294488_G_C	ENSG00000145362:ENST00000505342:exon8:c.G2772C:p.E924D,ENSG00000145362:ENST00000510275:exon13:c.G1536C:p.E512D,ENSG00000145362:ENST00000509550:exon23:c.G3015C:p.E1005D,ENSG00000145362:ENST00000264366:exon44:c.G11643C:p.E3881D,ENSG00000145362:ENST00000394537:exon44:c.G5487C:p.E1829D,ENSG00000145362:ENST00000357077:exon45:c.G11742C:p.E3914D,ENSG00000145362:ENST00000506722:exon46:c.G5460C:p.E1820D		S	0,00057

ANK2	4_114295915_A_G	ENST00000514960:exon19:c.2702-5A>G,ENST00000505342:exon9:c.2890-5A>G,ENST00000506344:exon2:c.134-5A>G		L	0,00057
ANK2	4_114296105_A_G	ENST00000514960:exon19:c.2884+3A>G,ENST00000505342:exon9:c.3072+3A>G,ENST00000506344:exon2:c.316+3A>G		L	0,00057
ANK2	4_114299338_G_A	ENST00000506344:exon3:c.408+1G>A,ENST00000514167:exon2:c.183+1G>A	rs180914830	D;L	0,00057
CASQ2	1_116243897_C_A	ENSG00000118729:ENST00000456138:exon9:c.G952T:p.D318Y,ENSG00000118729:ENST00000261448:exon11:c.G1165T:p.D389Y		N	0,00061
CASQ2	1_116243928_ATC_-	ENSG00000118729:ENST00000456138:exon9:c.919_921del:p.307_307del,ENSG00000118729:ENST00000261448:exon11:c.1132_1134del:p.378_378del		N	0,00060
CASQ2	1_116247824_C_T	ENSG00000118729:ENST00000456138:exon7:c.G715A:p.D239N,ENSG00000118729:ENST00000261448:exon9:c.G928A:p.D310N	rs141314684	D	0,00057
CASQ2	1_116269737_T_C	ENSG00000118729:ENST00000456138:exon4:c.A400G:p.K134E,ENSG00000118729:ENST00000261448:exon6:c.A613G:p.K205E		S	0,00057
CASQ2	1_116275587_C_G	ENSG00000118729:ENST00000456138:exon3:c.G328C:p.A110P,ENSG00000118729:ENST00000261448:exon5:c.G541C:p.A181P		N	0,00057
CASQ2	1_116283431_C_T	ENSG00000118729:ENST00000261448:exon3:c.G338A:p.S113N	rs199750975	D	0,00057
CASQ2	1_116287470_C_T	ENSG00000118729:ENST00000261448:exon2:c.G298A:p.A100T,ENSG00000118729:ENST00000456138:exon2:c.G298A:p.A100T		N	0,00057
CASQ2	1_116310926_T_C	ENST00000261448:exon2:c.234+3A>G,ENST00000456138:exon2:c.234+3A>G		L	0,00057
CAV3	3_8775602_G_A	ENSG00000182533:ENST00000343849:exon1:c.G40A:p.V14I,ENSG00000182533:ENST00000397368:exon1:c.G40A:p.V14I	rs121909281	D	0,00115
CAV3	3_8787374_G_A	ENSG00000182533:ENST00000343849:exon2:c.G277A:p.A93T,ENSG00000182533:ENST00000397368:exon2:c.G277A:p.A93T	rs28936686	D;P	0,00063
CSRP3	11_19204270_G_A	ENSG00000129170:ENST00000265968:exon6:c.C532T:p.P178S,ENSG00000129170:ENST00000533783:exon7:c.C532T:p.P178S		S	0,00057
CSRP3	11_19206558_C_T	ENSG00000129170:ENST00000265968:exon5:c.G449A:p.C150Y,ENSG00000129170:ENST00000533783:exon6:c.G449A:p.C150Y		S	0,00172
CSRP3	11_19206593_C_A	ENST00000265968:exon6:c.415-1G>T,ENST00000533783:exon7:c.415-1G>T		L	0,00115
CSRP3	11_19209682_C_G	ENST00000265968:exon4:c.281+1G>C,ENST00000533783:exon5:c.281+1G>C		L	0,00057
CSRP3	11_19209752_A_G	ENSG00000129170:ENST00000265968:exon3:c.T212C:p.I71T,ENSG00000129170:ENST00000533783:exon4:c.T212C:p.I71T		N	0,00057
CSRP3	11_19209773_C_T	ENSG00000129170:ENST00000265968:exon3:c.G191A:p.R64H,ENSG00000129170:ENST00000533783:exon4:c.G191A:p.R64H		S	0,00057

CSRP3	11_19209833_A_G	ENSG00000129170:ENST00000265968:exon3:c.T131C:p.L44P,ENSG00000129170:ENST00000533783:exon4:c.T131C:p.L44P	rs104894205	D;P	0,00172
CSRP3	11_19213968_A_G	ENSG00000129170:ENST00000265968:exon2:c.T28C:p.C10R,ENSG00000129170:ENST00000533783:exon3:c.T28C:p.C10R		S	0,00057
CSRP3	11_19213974_C_T	ENSG00000129170:ENST00000265968:exon2:c.G22A:p.A8T,ENSG00000129170:ENST00000533783:exon3:c.G22A:p.A8T	rs45531937	D	0,00057
DES	2_220284854_A_G	ENSG00000175084:ENST00000373960:exon2:c.A616G:p.N206D		N	0,00058
DES	2_220285382_GG_-			L	0,00059
DES	2_220288540_G_A	ENSG00000175084:ENST00000373960:exon7:c.G1286A:p.R429Q	rs200580581	D	0,00059
DSC2	18_28648063_C_T	ENSG00000134755:ENST00000280904:exon16:c.G2624A:p.R875Q		N	0,00057
DSC2	18_28648870_C_T	ENSG00000134755:ENST00000251081:exon15:c.G2498A:p.R833H,ENSG00000134755:ENST00000280904:exon15:c.G2498A:p.R833H		P	0,00058
DSC2	18_28648998_TCC_-	ENSG00000134755:ENST00000251081:exon15:c.2368_2370del:p.790_790del,ENSG00000134755:ENST00000280904:exon15:c.2368_2370del:p.790_790del		N	0,00058
DSC2	18_28649002_CCT_-	ENSG00000134755:ENST00000251081:exon15:c.2364_2366del:p.788_789del,ENSG00000134755:ENST00000280904:exon15:c.2364_2366del:p.788_789del		P	0,00058
DSC2	18_28649033_C_T	ENSG00000134755:ENST00000251081:exon15:c.G2335A:p.G779R,ENSG00000134755:ENST00000280904:exon15:c.G2335A:p.G779R	rs139290300	D;S	0,00058
DSC2	18_28649081_C_T	ENSG00000134755:ENST00000251081:exon15:c.G2287A:p.A763T,ENSG00000134755:ENST00000280904:exon15:c.G2287A:p.A763T		N	0,00057
DSC2	18_28650748_A_C	ENSG00000134755:ENST00000251081:exon14:c.T2194G:p.L732V,ENSG00000134755:ENST00000280904:exon14:c.T2194G:p.L732V	rs151024019	D;P	0,00458
DSC2	18_28651640_C_T	ENSG00000134755:ENST00000251081:exon13:c.G2056A:p.G686S,ENSG00000134755:ENST00000280904:exon13:c.G2056A:p.G686S		N	0,00057
DSC2	18_28654750_G_A	ENSG00000134755:ENST00000251081:exon12:c.C1787T:p.A596V,ENSG00000134755:ENST00000280904:exon12:c.C1787T:p.A596V	rs148185335	P	0,00115
DSC2	18_28654874_C_A	ENST00000251081:exon13:c.1664-1G>T,ENST00000280904:exon13:c.1664-1G>T		L	0,00058
DSC2	18_28662896_G_A	ENSG00000134755:ENST00000251081:exon8:c.C1073T:p.T358I,ENSG00000134755:ENST00000280904:exon8:c.C1073T:p.T358I	rs139399951	D	0,00057
DSC2	18_28666574_C_T	ENSG00000134755:ENST00000251081:exon7:c.G907A:p.V303M,ENSG00000134755:ENST00000280904:exon7:c.G907A:p.V303M	rs145560678	D;P	0,00229
DSC2	18_28666624_C_A	ENSG00000134755:ENST00000251081:exon7:c.G857T:p.G286V,ENSG00000134755:ENST00000280904:exon7:c.G857T:p.G286V	rs199682735	D	0,00057

DSC2	18_28666627_A_G	ENSG00000134755:ENST00000251081:exon7:c.T854C:p.I285T,ENSG00000134755:ENST00000280904:exon7:c.T854C:p.I285T	rs199918720	D	0,00057
DSC2	18_28666657_G_A	ENSG00000134755:ENST00000251081:exon7:c.C824T:p.T275M,ENSG00000134755:ENST00000280904:exon7:c.C824T:p.T275M		P	0,00057
DSC2	18_28671095_G_A	ENSG00000134755:ENST00000251081:exon4:c.C370T:p.H124Y,ENSG00000134755:ENST00000280904:exon4:c.C370T:p.H124Y		N	0,00057
DSC2	18_28672152_G_A	ENSG00000134755:ENST00000251081:exon3:c.C266T:p.S89L,ENSG00000134755:ENST00000280904:exon3:c.C266T:p.S89L	rs141379407	D;P	0,00057
DSC2	18_28672246_A_C	ENSG00000134755:ENST00000251081:exon3:c.T172G:p.F58V,ENSG00000134755:ENST00000280904:exon3:c.T172G:p.F58V	rs138749562	D;P	0,00057
DSC2	18_28681901_C_T	ENSG00000134755:ENST00000251081:exon1:c.G34A:p.G12R,ENSG00000134755:ENST00000280904:exon1:c.G34A:p.G12R		N	0,00127
DSC2	18_28681932_C_A	ENSG00000134755:ENST00000251081:exon1:c.G3T:p.M1I,ENSG00000134755:ENST00000280904:exon1:c.G3T:p.M1I		S	0,00153
DSG2	18_29099805_C_A	ENSG00000046604:ENST00000261590:exon3:c.C121A:p.H41N,ENSG00000046604:ENST00000585206:exon3:c.C121A:p.H41N	rs201499704	D;P	0,00114
DSG2	18_29101120_G_A	ENSG00000046604:ENST00000261590:exon5:c.G437A:p.R146H,ENSG00000046604:ENST00000585206:exon5:c.G437A:p.R146H		P	0,00058
DSG2	18_29102067_A_G	ENSG00000046604:ENST00000261590:exon6:c.A545G:p.N182S,ENSG00000046604:ENST00000585206:exon6:c.A545G:p.N182S		N	0,00057
DSG2	18_29104717_A_G	ENSG00000046604:ENST00000261590:exon8:c.A880G:p.K294E		P	0,00057
DSG2	18_29104798_T_A	ENSG00000046604:ENST00000261590:exon8:c.T961A:p.F321I	rs201040643	D;P	0,00057
DSG2	18_29104840_A_G	ENSG00000046604:ENST00000261590:exon8:c.A1003G:p.T335A	rs191564916	D;P	0,00057
DSG2	18_29110986_-_C	ENSG00000046604:ENST00000261590:exon9:c.1051_1052insC:p.S351fs		L	0,00057
DSG2	18_29110986_A_C	ENSG00000046604:ENST00000261590:exon9:c.A1051C:p.S351R		N	0,00057
DSG2	18_29115255_G_A	ENSG00000046604:ENST00000261590:exon10:c.G1303A:p.D435N		P	0,00057
DSG2	18_29115328_A_G	ENSG00000046604:ENST00000261590:exon10:c.A1376G:p.Y459C		S	0,00057
DSG2	18_29122591_A_G	ENSG00000046604:ENST00000261590:exon14:c.A2110G:p.I704V	rs141388237	D	0,00174
DSG2	18_29122696_A_T	ENSG00000046604:ENST00000261590:exon14:c.A2215T:p.I739F		N	0,00059
DSG2	18_29126099_C_T	ENSG00000046604:ENST00000261590:exon15:c.C2750T:p.A917V		P	0,00057
DSG2	18_29126255_C_T	ENSG00000046604:ENST00000261590:exon15:c.C2906T:p.A969V		S	0,00057
DSG2	18_29126389_G_A	ENSG00000046604:ENST00000261590:exon15:c.G3040A:p.V1014I	rs200830807	D;P	0,00057

DSG2	18_29126426_G_A	ENSG00000046604:ENST00000261590:exon15:c.G3077A:p.S1026N		S	0,00057
DSG2	18_29126458_A_G	ENSG00000046604:ENST00000261590:exon15:c.A3109G:p.N1037D		N	0,00057
DSG2	18_29126475_G_C	ENSG00000046604:ENST00000261590:exon15:c.G3126C:p.Q1042H		S	0,00057
DSG2	18_29126516_C_T	ENSG00000046604:ENST00000261590:exon15:c.C3167T:p.T1056I		P	0,00057
DSP	6_7562969_A_G	ENSG00000096696:ENST00000379802:exon5:c.A682G:p.I228V,ENSG00000096696:ENST00000418664:exon5:c.A682G:p.I228V		N	0,00058
DSP	6_7562975_G_A	ENSG00000096696:ENST00000379802:exon5:c.G688A:p.D230N,ENSG00000096696:ENST00000418664:exon5:c.G688A:p.D230N	rs147315869	D;P	0,00172
DSP	6_7564006_A_T	ENSG00000096696:ENST00000379802:exon6:c.A764T:p.Y255F,ENSG00000096696:ENST00000418664:exon6:c.A764T:p.Y255F		S	0,00057
DSP	6_7565758_G_T	ENST00000379802:exon7:c.939+5G>T,ENST00000418664:exon7:c.939+5G>T		L	0,00058
DSP	6_7569444_G_A	ENSG00000096696:ENST00000379802:exon12:c.G1445A:p.C482Y,ENSG00000096696:ENST00000418664:exon12:c.G1445A:p.C482Y		S	0,00058
DSP	6_7569482_G_A	ENSG00000096696:ENST00000379802:exon12:c.G1483A:p.V495M,ENSG00000096696:ENST00000418664:exon12:c.G1483A:p.V495M		N	0,00058
DSP	6_7570791_G_A	ENSG00000096696:ENST00000379802:exon13:c.G1696A:p.A566T,ENSG00000096696:ENST00000418664:exon13:c.G1696A:p.A566T	rs148147581	D;P	0,00059
DSP	6_7571692_A_G	ENSG00000096696:ENST00000379802:exon14:c.A1778G:p.N593S,ENSG00000096696:ENST00000418664:exon14:c.A1778G:p.N593S	rs34239595	D	0,00057
DSP	6_7574937_A_G	ENSG00000096696:ENST00000379802:exon17:c.A2345G:p.D782G,ENSG00000096696:ENST00000418664:exon17:c.A2345G:p.D782G		S	0,00057
DSP	6_7575014_C_T	ENSG00000096696:ENST00000379802:exon17:c.C2422T:p.R808C,ENSG00000096696:ENST00000418664:exon17:c.C2422T:p.R808C	rs150339369	D;S	0,00172
DSP	6_7576669_C_T	ENSG00000096696:ENST00000379802:exon19:c.C2773T:p.R925W,ENSG00000096696:ENST00000418664:exon19:c.C2773T:p.R925W	rs145933612	D;S	0,00057
DSP	6_7578770_T_C	ENSG00000096696:ENST00000379802:exon22:c.T3059C:p.M1020T,ENSG00000096696:ENST00000418664:exon22:c.T3059C:p.M1020T		N	0,00057
DSP	6_7580129_A_G	ENSG00000096696:ENST00000379802:exon23:c.A3706G:p.R1236G		S	0,00115
DSP	6_7580528_G_A	ENSG00000096696:ENST00000379802:exon23:c.G4105A:p.E1369K	rs141805096	D	0,00058
DSP	6_7580810_G_A	ENSG00000096696:ENST00000379802:exon23:c.G4387A:p.V1463I		N	0,00058
DSP	6_7580912_C_T	ENSG00000096696:ENST00000379802:exon23:c.C4489T:p.R1497W	rs148041814	D	0,00057
DSP	6_7580913_G_A	ENSG00000096696:ENST00000379802:exon23:c.G4490A:p.R1497Q		N	0,00057

DSP	6_7580981_A_T	ENSG00000096696:ENST00000379802:exon23:c.A4558T:p.S1520C		S	0,00058
DSP	6_7581198_A_G	ENSG00000096696:ENST00000379802:exon23:c.A4775G:p.K1592R	rs200421954	D;P	0,00120
DSP	6_7581420_G_A	ENSG00000096696:ENST00000379802:exon23:c.G4997A:p.R1666Q		N	0,00058
DSP	6_7581543_A_G	ENSG00000096696:ENST00000379802:exon23:c.A5120G:p.Q1707R		S	0,00057
DSP	6_7581601_C_A	ENSG00000096696:ENST00000379802:exon23:c.C5178A:p.N1726K	rs147415451	D;P	0,00057
DSP	6_7581644_G_A	ENSG00000096696:ENST00000379802:exon23:c.G5221A:p.A1741T		N	0,00057
DSP	6_7581786_A_G	ENSG00000096696:ENST00000379802:exon23:c.A5363G:p.Q1788R	rs139673146	D	0,00057
DSP	6_7583299_A_G	ENSG00000096696:ENST00000379802:exon24:c.A5804G:p.Y1935C,ENSG00000096696:ENST00000418664:exon24:c.A4007G:p.Y1336C		N	0,00058
DSP	6_7583347_G_A	ENSG00000096696:ENST00000379802:exon24:c.G5852A:p.R1951Q,ENSG00000096696:ENST00000418664:exon24:c.G4055A:p.R1352Q		N	0,00058
DSP	6_7583376_G_A	ENSG00000096696:ENST00000379802:exon24:c.G5881A:p.V1961I,ENSG00000096696:ENST00000418664:exon24:c.G4084A:p.V1362I		N	0,00058
DSP	6_7584376_C_G	ENSG00000096696:ENST00000379802:exon24:c.C6881G:p.A2294G,ENSG00000096696:ENST00000418664:exon24:c.C5084G:p.A1695G	rs147000526	D;P	0,00115
DSP	6_7584845_G_C	ENSG00000096696:ENST00000379802:exon24:c.G7350C:p.K2450N,ENSG00000096696:ENST00000418664:exon24:c.G5553C:p.K1851N		S	0,00057
DSP	6_7584922_A_T	ENSG00000096696:ENST00000379802:exon24:c.A7427T:p.E2476V,ENSG00000096696:ENST00000418664:exon24:c.A5630T:p.E1877V		S	0,00057
DSP	6_7585127_T_G	ENSG00000096696:ENST00000379802:exon24:c.T7632G:p.F2544L,ENSG00000096696:ENST00000418664:exon24:c.T5835G:p.F1945L		N	0,00057
DSP	6_7585233_G_T	ENSG00000096696:ENST00000379802:exon24:c.G7738T:p.D2580Y,ENSG00000096696:ENST00000418664:exon24:c.G5941T:p.D1981Y		N	0,00057
DSP	6_7585612_A_T	ENSG00000096696:ENST00000379802:exon24:c.A8117T:p.K2706M,ENSG00000096696:ENST00000418664:exon24:c.A6320T:p.K2107M		N	0,00059
DSP	6_7585686_T_C	ENSG00000096696:ENST00000379802:exon24:c.T8191C:p.Y2731H,ENSG00000096696:ENST00000418664:exon24:c.T6394C:p.Y2132H	rs201397978	D	0,00240
DSP	6_7585774_C_T	ENSG00000096696:ENST00000379802:exon24:c.C8279T:p.A2760V,ENSG00000096696:ENST00000418664:exon24:c.C6482T:p.A2161V		N	0,00058
DSP	6_7585921_C_T	ENSG00000096696:ENST00000379802:exon24:c.C8426T:p.S2809L,ENSG00000096696:ENST00000418664:exon24:c.C6629T:p.S2210L		N	0,00058
DSP	6_7585962_C_G	ENSG00000096696:ENST00000379802:exon24:c.C8467G:p.P2823A,ENSG00000096696:ENST00000418664:exon24:c.C6670G:p.P2224A	rs142717240	D;S	0,00115

DSP	6_7586019_C_T	ENSG00000096696:ENST00000379802:exon24:c.C8524T:p.R2842C,ENSG00000096696:ENST00000418664:exon24:c.C6727T:p.R2243C	rs144850908	D	0,00058
JUP	17_39912055_C_T	ENSG00000173801:ENST00000310706:exon14:c.G2179A:p.D727N,ENSG00000173801:ENST00000393930:exon14:c.G2179A:p.D727N,ENSG00000173801:ENST00000393931:exon14:c.G2179A:p.D727N		N	0,00093
JUP	17_39912444_T_C	ENSG00000173801:ENST00000310706:exon13:c.A2069G:p.N690S,ENSG00000173801:ENST00000393930:exon13:c.A2069G:p.N690S,ENSG00000173801:ENST00000393931:exon13:c.A2069G:p.N690S	rs147628503	D	0,00070
JUP	17_39919360_C_T	ENSG00000173801:ENST00000310706:exon8:c.G1372A:p.A458T,ENSG00000173801:ENST00000393930:exon8:c.G1372A:p.A458T,ENSG00000173801:ENST00000393931:exon8:c.G1372A:p.A458T	rs139559495	D	0,00080
JUP	17_39919408_T_A	ENSG00000173801:ENST00000310706:exon8:c.A1324T:p.I442F,ENSG00000173801:ENST00000393930:exon8:c.A1324T:p.I442F,ENSG00000173801:ENST00000393931:exon8:c.A1324T:p.I442F	rs142213474	D;S	0,00064
JUP	17_39921027_G_C	ENSG00000173801:ENST00000310706:exon7:c.C1096G:p.P366A,ENSG00000173801:ENST00000393930:exon7:c.C1096G:p.P366A,ENSG00000173801:ENST00000393931:exon7:c.C1096G:p.P366A		N	0,00070
JUP	17_39925401_C_T	ENSG00000173801:ENST00000540235:exon3:c.G527A:p.R176Q,ENSG00000173801:ENST00000310706:exon4:c.G527A:p.R176Q,ENSG00000173801:ENST00000393930:exon4:c.G527A:p.R176Q,ENSG00000173801:ENST00000393931:exon4:c.G527A:p.R176Q,ENSG00000173801:ENST00000420370:exon4:c.G527A:p.R176Q,ENSG00000173801:ENST00000437187:exon4:c.G527A:p.R176Q,ENSG00000173801:ENST00000449889:exon4:c.G527A:p.R176Q,ENSG00000173801:ENST00000424457:exon5:c.G527A:p.R176Q	rs144171604	D	0,00107
JUP	17_39925704_G_C	ENSG00000173801:ENST00000540235:exon2:c.C434G:p.P145R,ENSG00000173801:ENST00000310706:exon3:c.C434G:p.P145R,ENSG00000173801:ENST00000393930:exon3:c.C434G:p.P145R,ENSG00000173801:ENST00000393931:exon3:c.C434G:p.P145R,ENSG00000173801:ENST00000420370:exon3:c.C434G:p.P145R,ENSG00000173801:ENST00000437187:exon3:c.C434G:p.P145R,ENSG00000173801:ENST00000449889:exon3:c.C434G:p.P145R,ENSG00000173801:ENST00000424457:exon4:c.C434G:p.P145R		S	0,00085
KCNE1	21_35821619_G_A	ENSG00000180509:ENST00000416357:exon2:c.C314T:p.S105L,ENSG00000180509:ENST00000337385:exon3:c.C314T:p.S105L,ENSG00000180509:ENST00000399284:exon3:c.C314T:p.S105L,ENSG00000180509:ENST00000399289:exon3:c.C314T:p.S105L,ENSG00000180509:ENST00000432085:exon3:c.C314T:p.S105L,ENSG00000180509:ENST00000399286:exon4:c.C314T:p.S105L		N	0,00057
KCNE1	21_35821827_G_A	ENSG00000180509:ENST00000416357:exon2:c.C106T:p.R36C,ENSG00000180509:ENST00000337385:exon3:c.C106T:p.R36C,ENSG00000180509:ENST00000399284:exon3:c.C106T:p.R36C,ENSG00000180509:ENST00000399289:exon3:c.C106T:p.R36C,ENSG00000180509:ENST00000432085:exon3:c.C106T:p.R36C,ENSG00000180509:ENST00000399286:exon4:c.C106T:p.R36C		N	0,00058

KCNE1	21_35821850_G_A	ENSG00000180509:ENST00000416357:exon2:c.C83T:p.S28L,ENSG00000180509:ENST00000337385:exon3:c.C83T:p.S28L,ENSG00000180509:ENST00000399284:exon3:c.C83T:p.S28L,ENSG00000180509:ENST00000399289:exon3:c.C83T:p.S28L,ENSG00000180509:ENST00000432085:exon3:c.C83T:p.S28L,ENSG00000180509:ENST00000399286:exon4:c.C83T:p.S28L	rs199473350	D;P	0,00058
KCNE1	21_35821916_G_T	ENSG00000180509:ENST00000416357:exon2:c.C17A:p.T6N,ENSG00000180509:ENST00000337385:exon3:c.C17A:p.T6N,ENSG00000180509:ENST00000399284:exon3:c.C17A:p.T6N,ENSG00000180509:ENST00000399289:exon3:c.C17A:p.T6N,ENSG00000180509:ENST00000432085:exon3:c.C17A:p.T6N,ENSG00000180509:ENST00000399286:exon4:c.C17A:p.T6N		N	0,00058
KCNE2	21_35736455_G_A			L	0,00057
KCNE2	21_35742806_C_T	ENSG00000159197:ENST00000290310:exon2:c.C29T:p.T10M	rs199473648	D;P	0,00057
KCNE2	21_35742817_G_A	ENSG00000159197:ENST00000290310:exon2:c.G40A:p.V14I	rs142153692	D;P	0,00115
KCNE2	21_35742938_T_C	ENSG00000159197:ENST00000290310:exon2:c.T161C:p.M54T	rs74315447	D;P	0,00057
KCNE2	21_35742947_T_C	ENSG00000159197:ENST00000290310:exon2:c.T170C:p.I57T	rs74315448	D;P	0,00057
KCNE2	21_35743134_C_A	ENSG00000159197:ENST00000290310:exon2:c.C357A:p.F119L	rs139202426	D	0,00058
KCNH2	7_150642517_G_A	ENSG00000055118:ENST00000330883:exon11:c.C2396T:p.P799L,ENSG00000055118:ENST00000392968:exon13:c.C3128T:p.P1043L,ENSG00000055118:ENST00000262186:exon15:c.C3416T:p.P1139L		N	0,00093
KCNH2	7_150644131_C_T	ENSG00000055118:ENST00000330883:exon10:c.G2144A:p.R715Q,ENSG00000055118:ENST00000392968:exon12:c.G2876A:p.R959Q,ENSG00000055118:ENST00000262186:exon14:c.G3164A:p.R1055Q	rs41307270	D;S	0,00063
KCNH2	7_150644718_T_C	ENSG00000055118:ENST00000330883:exon8:c.A1921G:p.S641G,ENSG00000055118:ENST00000392968:exon10:c.A2653G:p.S885G,ENSG00000055118:ENST00000262186:exon12:c.A2941G:p.S981G	rs76649554	D	0,00111
KCNH2	7_150645607_C_T	ENSG00000055118:ENST00000330883:exon7:c.G1597A:p.G533S,ENSG00000055118:ENST00000392968:exon9:c.G2329A:p.G777S,ENSG00000055118:ENST00000262186:exon11:c.G2617A:p.G873S	rs41314354	D	0,00117
KCNH2	7_150647078_G_A	ENSG00000055118:ENST00000430723:exon9:c.C2576T:p.T859M	rs41314366	D;S	0,00059
KCNH2	7_150647144_C_A	ENSG00000055118:ENST00000430723:exon9:c.G2510T:p.G837V		N	0,00060
KCNH2	7_150647353_GT_-	ENSG00000055118:ENST00000330883:exon5:c.1280_1281del:p.427_427del,ENSG00000055118:ENST00000392968:exon7:c.2012_2013del:p.671_671del,ENSG00000055118:ENST00000262186:exon9:c.2300_2301del:p.767_767del,ENSG00000055118:ENST00000430723:exon9:c.2300_2301del:p.767_767del		N	0,00073
KCNH2	7_150654468_G_A	ENSG00000055118:ENST00000392968:exon3:c.C751T:p.P251S,ENSG00000055118:ENST00000262186:exon5:c.C1039T:p.P347S,ENSG00000055118:ENST00000430723:exon5:c.C1039T:p.P347S	rs138776684	D;P	0,00118
KCNH2	7_150655425_T_C	ENSG00000055118:ENST00000392968:exon2:c.A350G:p.D117G,ENSG00000055118:ENST00000262186:exon4:c.A638G:p.D213G,ENSG00000055118:ENST00000430723:exon4:c.A638G:p.D213G		N	0,00148

KCNH2	7_150655495_CGCCCGCGC_-	ENSG00000055118:ENST00000392968:exon2:c.272_280del:p.91_94del,ENSG00000055118:ENST00000262186:exon4:c.560_568del:p.187_190del,ENSG00000055118:ENST00000430723:exon4:c.560_568del:p.187_190del		P	0,01220
KCNH2	7_150655510_CGCCGCCCG_-	ENSG00000055118:ENST00000392968:exon2:c.257_265del:p.86_89del,ENSG00000055118:ENST00000262186:exon4:c.545_553del:p.182_185del,ENSG00000055118:ENST00000430723:exon4:c.545_553del:p.182_185del		N	0,01124
KCNH2	7_150655521_C_T	ENSG00000055118:ENST00000392968:exon2:c.G254A:p.R85Q,ENSG00000055118:ENST00000262186:exon4:c.G542A:p.R181Q,ENSG00000055118:ENST00000430723:exon4:c.G542A:p.R181Q	rs41308954	D	0,01099
KCNH2	7_150655537_G_A	ENSG00000055118:ENST00000392968:exon2:c.C238T:p.R80W,ENSG00000055118:ENST00000262186:exon4:c.C526T:p.R176W,ENSG00000055118:ENST00000430723:exon4:c.C526T:p.R176W	rs36210422	D;S	0,00543
KCNH2	7_150656690_G_A	ENSG00000055118:ENST00000392968:exon1:c.C154T:p.R52W,ENSG00000055118:ENST00000262186:exon3:c.C442T:p.R148W,ENSG00000055118:ENST00000430723:exon3:c.C442T:p.R148W	rs139544114	D	0,00140
KCNH2	7_150674983_G_A	ENSG00000055118:ENST00000262186:exon1:c.C19T:p.H7Y,ENSG00000055118:ENST00000430723:exon1:c.C19T:p.H7Y		S	0,00175
KCNJ2	17_68172133_A_G	ENSG00000123700:ENST00000243457:exon2:c.A953G:p.N318S,ENSG00000123700:ENST00000535240:exon2:c.A953G:p.N318S		N	0,00057
KCNQ1	11_2466480_-_CGCGCCCAT	ENSG00000053918:ENST00000155840:exon1:c.152_153insCGCGCCCAT:p.Y51delinsYAPI		N	0,01376
KCNQ1	11_2466545_C_A	ENSG00000053918:ENST00000155840:exon1:c.C217A:p.P73T	rs199472676	D;P	0,00110
KCNQ1	11_2542757_C_T	ENSG00000053918:ENST00000380776:exon2:c.C133T:p.R45C	rs80269976	D	0,00058
KCNQ1	11_2542793_G_A	ENSG00000053918:ENST00000380776:exon2:c.G169A:p.D57N	rs10400212	D	0,00059
KCNQ1	11_2549229_C_T	ENSG00000053918:ENST00000155840:exon2:c.C458T:p.T153M,ENSG00000053918:ENST00000335475:exon2:c.C77T:p.T26M,ENSG00000053918:ENST00000496887:exon3:c.C197T:p.T66M	rs143709408	D;P	0,00068
KCNQ1	11_2591921_C_T	ENSG00000053918:ENST00000155840:exon3:c.C541T:p.R181C,ENSG00000053918:ENST00000335475:exon3:c.C160T:p.R54C,ENSG00000053918:ENST00000496887:exon4:c.C280T:p.R94C	rs199473395	D;P	0,00094
KCNQ1	11_2594092_T_G	ENSG00000053918:ENST00000155840:exon6:c.T797G:p.L266R,ENSG00000053918:ENST00000335475:exon6:c.T416G:p.L139R,ENSG00000053918:ENST00000496887:exon7:c.T536G:p.L179R		S	0,00062
KCNQ1	11_2604687_A_T	ENSG00000053918:ENST00000155840:exon7:c.A944T:p.Y315F,ENSG00000053918:ENST00000335475:exon7:c.A563T:p.Y188F	rs74462309	D;P	0,00074
KCNQ1	11_2608850_G_T	ENSG00000053918:ENST00000155840:exon9:c.G1179T:p.K393N,ENSG00000053918:ENST00000335475:exon9:c.G798T:p.K266N	rs12720457	D	0,00158
KCNQ1	11_2790079_G_A	ENSG00000053918:ENST00000155840:exon12:c.G1520A:p.R507Q,ENSG00000053918:ENST00000335475:exon12:c.G1139A:p.R380Q		N	0,00058
KCNQ1	11_2797186_G_A	ENST00000155840:exon13:c.1591-4G>A,ENST00000335475:exon13:c.1210-4G>A		L	0,00085

KCNQ1	11_2869087_G_A	ENSG00000053918:ENST00000155840:exon16:c.G1885A:p.G629S,ENSG00000053918:ENST00000335475:exon16:c.G1504A:p.G502S		N	0,00097
KCNQ1	11_2869105_G_A	ENSG00000053918:ENST00000155840:exon16:c.G1903A:p.G635R,ENSG00000053918:ENST00000335475:exon16:c.G1522A:p.G508R	rs199473484	D;P	0,00097
KCNQ1	11_2869127_G_T	ENSG00000053918:ENST00000155840:exon16:c.G1925T:p.C642F,ENSG00000053918:ENST00000335475:exon16:c.G1544T:p.C515F		N	0,00096
LDB3	10_88428332_G_C			L	0,00070
LDB3	10_88439193_G_A	ENSG00000122367:ENST00000263066:exon2:c.G163A:p.V55I,ENSG00000122367:ENST00000310944:exon2:c.G163A:p.V55I,ENSG00000122367:ENST00000361373:exon2:c.G163A:p.V55I,ENSG00000122367:ENST00000372056:exon2:c.G163A:p.V55I,ENSG00000122367:ENST00000372066:exon2:c.G163A:p.V55I,ENSG00000122367:ENST00000542786:exon2:c.G163A:p.V55I,ENSG00000122367:ENST00000352360:exon3:c.G163A:p.V55I,ENSG00000122367:ENST00000429277:exon3:c.G163A:p.V55I,ENSG00000122367:ENST00000458213:exon3:c.G163A:p.V55I	rs3740343	D;P	0,00115
LDB3	10_88441437_C_T	ENSG00000122367:ENST00000310944:exon4:c.C566T:p.S189L,ENSG00000122367:ENST00000361373:exon4:c.C566T:p.S189L,ENSG00000122367:ENST00000372056:exon4:c.C566T:p.S189L,ENSG00000122367:ENST00000542786:exon4:c.C566T:p.S189L,ENSG00000122367:ENST00000429277:exon5:c.C566T:p.S189L	rs45487699	D;P	0,00072
LDB3	10_88441535_G_A	ENSG00000122367:ENST00000310944:exon4:c.G664A:p.A222T,ENSG00000122367:ENST00000361373:exon4:c.G664A:p.A222T,ENSG00000122367:ENST00000372056:exon4:c.G664A:p.A222T,ENSG00000122367:ENST00000542786:exon4:c.G664A:p.A222T,ENSG00000122367:ENST00000429277:exon5:c.G664A:p.A222T	rs139922045	D	0,00066
LDB3	10_88446940_G_C	ENSG00000122367:ENST00000263066:exon5:c.G459C:p.Q153H,ENSG00000122367:ENST00000372056:exon5:c.G804C:p.Q268H,ENSG00000122367:ENST00000372066:exon5:c.G459C:p.Q153H,ENSG00000122367:ENST00000429277:exon6:c.G804C:p.Q268H,ENSG00000122367:ENST00000458213:exon6:c.G459C:p.Q153H		N	0,00067
LDB3	10_88446992_G_A	ENSG00000122367:ENST00000263066:exon5:c.G511A:p.A171T,ENSG00000122367:ENST00000372056:exon5:c.G856A:p.A286T,ENSG00000122367:ENST00000372066:exon5:c.G511A:p.A171T,ENSG00000122367:ENST00000429277:exon6:c.G856A:p.A286T,ENSG00000122367:ENST00000458213:exon6:c.G511A:p.A171T		N	0,00066
LDB3	10_88466427_G_A	ENSG00000122367:ENST00000361373:exon7:c.G1036A:p.A346T	rs201968775	D	0,00094
LDB3	10_88466440_C_T	ENSG00000122367:ENST00000361373:exon7:c.C1049T:p.T350I	rs200796750	D;P	0,00092
LDB3	10_88469687_G_A	ENSG00000122367:ENST00000352360:exon5:c.G340A:p.A114T,ENSG00000122367:ENST00000263066:exon8:c.G781A:p.A261T,ENSG00000122367:ENST00000361373:exon8:c.G1111A:p.A371T,ENSG00000122367:ENST00000429277:exon9:c.G1126A:p.A376T,ENSG00000122367:ENST00000458213:exon9:c.G781A:p.A261T	rs45539535	D;P	0,00143

LDB3	10_88476312_G_A	ENSG00000122367:ENST00000352360:exon6:c.G689A:p.R230H,ENSG00000122367:ENST00000263066:exon9:c.G1130A:p.R377H,ENSG00000122367:ENST00000361373:exon9:c.G1460A:p.R487H,ENSG00000122367:ENST00000429277:exon10:c.G1475A:p.R492H,ENSG00000122367:ENST00000458213:exon10:c.G1130A:p.R377H	rs146265188	D;S	0,00088
LDB3	10_88476339_T_C	ENSG00000122367:ENST00000352360:exon6:c.T716C:p.F239S,ENSG00000122367:ENST00000263066:exon9:c.T1157C:p.F386S,ENSG00000122367:ENST00000361373:exon9:c.T1487C:p.F496S,ENSG00000122367:ENST00000429277:exon10:c.T1502C:p.F501S,ENSG00000122367:ENST00000458213:exon10:c.T1157C:p.F386S	rs147072071	D	0,00083
LDB3	10_88476446_G_C	ENSG00000122367:ENST00000352360:exon6:c.G823C:p.A275P,ENSG00000122367:ENST00000263066:exon9:c.G1264C:p.A422P,ENSG00000122367:ENST00000361373:exon9:c.G1594C:p.A532P,ENSG00000122367:ENST00000429277:exon10:c.G1609C:p.A537P,ENSG00000122367:ENST00000458213:exon10:c.G1264C:p.A422P	rs143764931	D;S	0,00258
LDB3	10_88476524_A_G	ENSG00000122367:ENST00000352360:exon6:c.A901G:p.I301V,ENSG00000122367:ENST00000263066:exon9:c.A1342G:p.I448V,ENSG00000122367:ENST00000361373:exon9:c.A1672G:p.I558V,ENSG00000122367:ENST00000429277:exon10:c.A1687G:p.I563V,ENSG00000122367:ENST00000458213:exon10:c.A1342G:p.I448V		P	0,00079
LMNA	1_156096679_G_A	ENSG00000160789:ENST00000392353:exon1:c.G86A:p.R29K,ENSG00000160789:ENST00000368297:exon2:c.G86A:p.R29K,ENSG00000160789:ENST00000515459:exon2:c.G86A:p.R29K		N	0,00123
LMNA	1_156096738_C_T			L	0,00067
LMNA	1_156105051_C_T	ENSG00000160789:ENST00000347559:exon5:c.C884T:p.S295L,ENSG00000160789:ENST00000361308:exon5:c.C884T:p.S295L,ENSG00000160789:ENST00000368299:exon5:c.C884T:p.S295L,ENSG00000160789:ENST00000368300:exon5:c.C884T:p.S295L,ENSG00000160789:ENST00000392353:exon5:c.C641T:p.S214L,ENSG00000160789:ENST00000448611:exon5:c.C548T:p.S183L,ENSG00000160789:ENST00000368297:exon6:c.C641T:p.S214L,ENSG00000160789:ENST00000473598:exon6:c.C587T:p.S196L,ENSG00000160789:ENST00000368301:exon8:c.C884T:p.S295L		N	0,00081
LMNA	1_156105062_A_G	ENSG00000160789:ENST00000347559:exon5:c.A895G:p.I299V,ENSG00000160789:ENST00000361308:exon5:c.A895G:p.I299V,ENSG00000160789:ENST00000368299:exon5:c.A895G:p.I299V,ENSG00000160789:ENST00000368300:exon5:c.A895G:p.I299V,ENSG00000160789:ENST00000392353:exon5:c.A652G:p.I218V,ENSG00000160789:ENST00000448611:exon5:c.A559G:p.I187V,ENSG00000160789:ENST00000368297:exon6:c.A652G:p.I218V,ENSG00000160789:ENST00000473598:exon6:c.A598G:p.I200V,ENSG00000160789:ENST00000368301:exon8:c.A895G:p.I299V	rs150924946	D	0,00083

LMNA	1_156105708_C_T	ENSG00000160789:ENST00000347559:exon6:c.C953T:p.A318V,ENSG00000160789:ENST00000361308:exon6:c.C953T:p.A318V,ENSG00000160789:ENST00000368299:exon6:c.C953T:p.A318V,ENSG00000160789:ENST00000368300:exon6:c.C953T:p.A318V,ENSG00000160789:ENST00000392353:exon6:c.C710T:p.A237V,ENSG00000160789:ENST00000448611:exon6:c.C617T:p.A206V,ENSG00000160789:ENST00000368297:exon7:c.C710T:p.A237V,ENSG00000160789:ENST00000473598:exon7:c.C656T:p.A219V,ENSG00000160789:ENST00000368301:exon9:c.C953T:p.A318V		N	0,00063
LMNA	1_156106078_G_T	ENSG00000160789:ENST00000508500:exon2:c.G109T:p.G37C,ENSG00000160789:ENST00000347559:exon7:c.G1231T:p.G411C,ENSG00000160789:ENST00000361308:exon7:c.G1231T:p.G411C,ENSG00000160789:ENST00000368299:exon7:c.G1231T:p.G411C,ENSG00000160789:ENST00000368300:exon7:c.G1231T:p.G411C,ENSG00000160789:ENST00000392353:exon7:c.G988T:p.G330C,ENSG00000160789:ENST00000448611:exon7:c.G895T:p.G299C,ENSG00000160789:ENST00000368297:exon8:c.G988T:p.G330C,ENSG00000160789:ENST00000473598:exon8:c.G934T:p.G312C,ENSG00000160789:ENST00000368301:exon10:c.G1231T:p.G411C		N	0,00076
LMNA	1_156108453_A_C	ENSG00000160789:ENST00000508500:exon5:c.A661C:p.S221R,ENSG00000160789:ENST00000347559:exon10:c.A1783C:p.S595R,ENSG00000160789:ENST00000368300:exon11:c.A1873C:p.S625R,ENSG00000160789:ENST00000448611:exon11:c.A1537C:p.S513R,ENSG00000160789:ENST00000473598:exon12:c.A1576C:p.S526R		N	0,00076
LMNA	1_156108454_G_C	ENSG00000160789:ENST00000508500:exon5:c.G662C:p.S221T,ENSG00000160789:ENST00000347559:exon10:c.G1784C:p.S595T,ENSG00000160789:ENST00000368300:exon11:c.G1874C:p.S625T,ENSG00000160789:ENST00000448611:exon11:c.G1538C:p.S513T,ENSG00000160789:ENST00000473598:exon12:c.G1577C:p.S526T		N	0,00076
LMNA	1_156108510_C_T	ENSG00000160789:ENST00000508500:exon5:c.C718T:p.R240C,ENSG00000160789:ENST00000347559:exon10:c.C1840T:p.R614C,ENSG00000160789:ENST00000368300:exon11:c.C1930T:p.R644C,ENSG00000160789:ENST00000448611:exon11:c.C1594T:p.R532C,ENSG00000160789:ENST00000473598:exon12:c.C1633T:p.R545C	rs142000963	D;S	0,00151
MYBPC3	11_47353626_G_A	ENSG00000134571:ENST00000256993:exon31:c.C3808T:p.R1270X,ENSG00000134571:ENST00000399249:exon32:c.C3811T:p.R1271X,ENSG00000134571:ENST00000545968:exon33:c.C3811T:p.R1271X		P	0,00061
MYBPC3	11_47353639_G_C	ENSG00000134571:ENST00000256993:exon31:c.C3795G:p.C1265W,ENSG00000134571:ENST00000399249:exon32:c.C3798G:p.C1266W,ENSG00000134571:ENST00000545968:exon33:c.C3798G:p.C1266W		S	0,00060
MYBPC3	11_47353666_G_T	ENSG00000134571:ENST00000256993:exon31:c.C3768A:p.N1256K,ENSG00000134571:ENST00000399249:exon32:c.C3771A:p.N1257K,ENSG00000134571:ENST00000545968:exon33:c.C3771A:p.N1257K		S	0,00237
MYBPC3	11_47353674_C_T	ENSG00000134571:ENST00000256993:exon31:c.G3760A:p.A1254T,ENSG00000134571:ENST00000399249:exon32:c.G3763A:p.A1255T,ENSG00000134571:ENST00000545968:exon33:c.G3763A:p.A1255T		P	0,00117

MYBPC3	11_47353685_T_C	ENSG00000134571:ENST00000256993:exon31:c.A3749G:p.Y1250C,ENSG00000134571:ENST00000399249:exon32:c.A3752G:p.Y1251C,ENSG00000134571:ENST00000545968:exon33:c.A3752G:p.Y1251C		P	0,00059
MYBPC3	11_47353686_A_G	ENSG00000134571:ENST00000256993:exon31:c.T3748C:p.Y1250H,ENSG00000134571:ENST00000399249:exon32:c.T3751C:p.Y1251H,ENSG00000134571:ENST00000545968:exon33:c.T3751C:p.Y1251H		S	0,00058
MYBPC3	11_47353709_G_C	ENSG00000134571:ENST00000256993:exon31:c.C3725G:p.P1242R,ENSG00000134571:ENST00000399249:exon32:c.C3728G:p.P1243R,ENSG00000134571:ENST00000545968:exon33:c.C3728G:p.P1243R		S	0,00058
MYBPC3	11_47353740_G_A	ENSG00000134571:ENST00000256993:exon31:c.C3694T:p.Q1232X,ENSG00000134571:ENST00000399249:exon32:c.C3697T:p.Q1233X,ENSG00000134571:ENST00000545968:exon33:c.C3697T:p.Q1233X		P	0,00058
MYBPC3	11_47353755_G_A	ENSG00000134571:ENST00000256993:exon31:c.C3679T:p.R1227C,ENSG00000134571:ENST00000399249:exon32:c.C3682T:p.R1228C,ENSG00000134571:ENST00000545968:exon33:c.C3682T:p.R1228C	rs201312636	D;S	0,00057
MYBPC3	11_47354116_C_T	ENST00000256993:exon31:c.3624+1G>A,ENST00000399249:exon32:c.3627+1G>A,ENST00000545968:exon33:c.3627+1G>A		P	0,00059
MYBPC3	11_47354123_G_-	ENSG00000134571:ENST00000256993:exon30:c.3618delC:p.S1206fs,ENSG00000134571:ENST00000399249:exon31:c.3621delC:p.S1207fs,ENSG00000134571:ENST00000545968:exon32:c.3621delC:p.S1207fs		L	0,00059
MYBPC3	11_47354139_CAGCAGAGCA_-	ENSG00000134571:ENST00000256993:exon30:c.3593_3602del:p.1198_1201del,ENSG00000134571:ENST00000399249:exon31:c.3596_3605del:p.1199_1202del,ENSG00000134571:ENST00000545968:exon32:c.3596_3605del:p.1199_1202del		L	0,00059
MYBPC3	11_47354151_GCAGTGTAGCCC_-	ENSG00000134571:ENST00000256993:exon30:c.3579_3590del:p.1193_1197del,ENSG00000134571:ENST00000399249:exon31:c.3582_3593del:p.1194_1198del,ENSG00000134571:ENST00000545968:exon32:c.3582_3593del:p.1194_1198del		N	0,00118
MYBPC3	11_47354172_G_A	ENSG00000134571:ENST00000256993:exon30:c.C3569T:p.S1190L,ENSG00000134571:ENST00000399249:exon31:c.C3572T:p.S1191L,ENSG00000134571:ENST00000545968:exon32:c.C3572T:p.S1191L		P	0,00060
MYBPC3	11_47354209_C_T	ENSG00000134571:ENST00000256993:exon30:c.G3532A:p.E1178K,ENSG00000134571:ENST00000399249:exon31:c.G3535A:p.E1179K,ENSG00000134571:ENST00000545968:exon32:c.G3535A:p.E1179K	rs199669878	D;P	0,00059
MYBPC3	11_47354256_G_C	ENST00000256993:exon31:c.3488-3C>G,ENST00000399249:exon32:c.3491-3C>G,ENST00000545968:exon33:c.3491-3C>G		L	0,00059
MYBPC3	11_47354389_CCTTGGTGGTGG_-	ENSG00000134571:ENST00000256993:exon29:c.3452_3463del:p.1151_1155del,ENSG00000134571:ENST00000399249:exon30:c.3455_3466del:p.1152_1156del,ENSG00000134571:ENST00000545968:exon31:c.3455_3466del:p.1152_1156del		N	0,00059

MYBPC3	11_47354451_TAG_-	ENSG00000134571:ENST00000256993:exon29:c.3399_3401del:p.1133_1134del,ENSG00000134571:ENST00000399249:exon30:c.3402_3404del:p.1134_1135del,ENSG00000134571:ENST00000545968:exon31:c.3402_3404del:p.1134_1135del		N	0,00059
MYBPC3	11_47354471_C_G	ENSG00000134571:ENST00000256993:exon29:c.G3381C:p.E1127D,ENSG00000134571:ENST00000399249:exon30:c.G3384C:p.E1128D,ENSG00000134571:ENST00000545968:exon31:c.G3384C:p.E1128D		N	0,00059
MYBPC3	11_47354522_-_CAC	ENSG00000134571:ENST00000256993:exon29:c.3330_3331insGTG:p.E1110delinsEW,ENSG00000134571:ENST00000399249:exon30:c.3333_3334insGTG:p.E1111delinsEW,ENSG00000134571:ENST00000545968:exon31:c.3333_3334insGTG:p.E1111delinsEW		N	0,00062
MYBPC3	11_47354740_C_G	ENST00000256993:exon29:c.3327+5G>C,ENST00000399249:exon30:c.3330+5G>C,ENST00000545968:exon31:c.3330+5G>C		P	0,00348
MYBPC3	11_47354749_G_A	ENSG00000134571:ENST00000256993:exon28:c.C3323T:p.T1108I,ENSG00000134571:ENST00000399249:exon29:c.C3326T:p.T1109I,ENSG00000134571:ENST00000545968:exon30:c.C3326T:p.T1109I		P	0,00138
MYBPC3	11_47354781_C_T	ENSG00000134571:ENST00000256993:exon28:c.G3291A:p.W1097X,ENSG00000134571:ENST00000399249:exon29:c.G3294A:p.W1098X,ENSG00000134571:ENST00000545968:exon30:c.G3294A:p.W1098X		P	0,00071
MYBPC3	11_47354818_C_T	ENSG00000134571:ENST00000256993:exon28:c.G3254A:p.W1085X,ENSG00000134571:ENST00000399249:exon29:c.G3257A:p.W1086X,ENSG00000134571:ENST00000545968:exon30:c.G3257A:p.W1086X		L	0,00075
MYBPC3	11_47354848_-_A	ENSG00000134571:ENST00000256993:exon28:c.3224_3225insT:p.D1075fs,ENSG00000134571:ENST00000399249:exon29:c.3227_3228insT:p.D1076fs,ENSG00000134571:ENST00000545968:exon30:c.3227_3228insT:p.D1076fs		P	0,00378
MYBPC3	11_47355103_C_T	ENST00000256993:exon28:c.3187+5G>A,ENST00000399249:exon29:c.3190+5G>A,ENST00000545968:exon30:c.3190+5G>A		P	0,00134
MYBPC3	11_47355191_C_T	ENSG00000134571:ENST00000256993:exon27:c.G3104A:p.R1035H,ENSG00000134571:ENST00000399249:exon28:c.G3107A:p.R1036H,ENSG00000134571:ENST00000545968:exon29:c.G3107A:p.R1036H		N	0,00062
MYBPC3	11_47355233_C_G	ENSG00000134571:ENST00000256993:exon27:c.G3062C:p.R1021P,ENSG00000134571:ENST00000399249:exon28:c.G3065C:p.R1022P,ENSG00000134571:ENST00000545968:exon29:c.G3065C:p.R1022P		P	0,00063
MYBPC3	11_47355249_C_T	ENSG00000134571:ENST00000256993:exon27:c.G3046A:p.E1016K,ENSG00000134571:ENST00000399249:exon28:c.G3049A:p.E1017K,ENSG00000134571:ENST00000545968:exon29:c.G3049A:p.E1017K		P	0,00064
MYBPC3	11_47355294_G_A	ENSG00000134571:ENST00000256993:exon27:c.C3001T:p.R1001W,ENSG00000134571:ENST00000399249:exon28:c.C3004T:p.R1002W,ENSG00000134571:ENST00000545968:exon29:c.C3004T:p.R1002W	rs3729799	D	0,00065

MYBPC3	11_47355304_C_T	ENST00000256993:exon28:c.2992-1G>A,ENST00000399249:exon29:c.2995-1G>A,ENST00000545968:exon30:c.2995-1G>A		L	0,00066
MYBPC3	11_47355514_T_A	ENSG00000134571:ENST00000256993:exon26:c.A2950T:p.K984X,ENSG00000134571:ENST00000399249:exon27:c.A2953T:p.K985X,ENSG00000134571:ENST00000545968:exon28:c.A2953T:p.K985X		L	0,00068
MYBPC3	11_47356588_C_A	ENST00000256993:exon26:c.2902+5G>T,ENST00000399249:exon27:c.2905+5G>T,ENST00000545968:exon28:c.2905+5G>T		L	0,00074
MYBPC3	11_47356592_C_T	ENST00000256993:exon26:c.2902+1G>A,ENST00000399249:exon27:c.2905+1G>A,ENST00000545968:exon28:c.2905+1G>A		P	0,00147
MYBPC3	11_47356633_AG_-	ENSG00000134571:ENST00000256993:exon25:c.2861_2862del:p.954_954del,ENSG00000134571:ENST00000399249:exon26:c.2864_2865del:p.955_955del,ENSG00000134571:ENST00000545968:exon27:c.2864_2865del:p.955_955del		L	0,00156
MYBPC3	11_47356671_G_A	ENSG00000134571:ENST00000256993:exon25:c.C2824T:p.R942X,ENSG00000134571:ENST00000399249:exon26:c.C2827T:p.R943X,ENSG00000134571:ENST00000545968:exon27:c.C2827T:p.R943X		P	0,00312
MYBPC3	11_47356710_G_C	ENSG00000134571:ENST00000256993:exon25:c.C2785G:p.L929V,ENSG00000134571:ENST00000399249:exon26:c.C2788G:p.L930V,ENSG00000134571:ENST00000545968:exon27:c.C2788G:p.L930V		N	0,00082
MYBPC3	11_47357425_CA_-			P	0,00061
MYBPC3	11_47357437_G_T	ENSG00000134571:ENST00000256993:exon24:c.C2725A:p.P909T,ENSG00000134571:ENST00000399249:exon25:c.C2728A:p.P910T,ENSG00000134571:ENST00000545968:exon26:c.C2728A:p.P910T		P	0,00060
MYBPC3	11_47357547_G_T	ENSG00000134571:ENST00000256993:exon24:c.C2615A:p.P872H,ENSG00000134571:ENST00000399249:exon25:c.C2618A:p.P873H,ENSG00000134571:ENST00000545968:exon26:c.C2618A:p.P873H		P	0,00063
MYBPC3	11_47357555_G_-	ENSG00000134571:ENST00000256993:exon24:c.2607delC:p.P869fs,ENSG00000134571:ENST00000399249:exon25:c.2610delC:p.P870fs,ENSG00000134571:ENST00000545968:exon26:c.2610delC:p.P870fs		P	0,00252
MYBPC3	11_47357560_G_T	ENSG00000134571:ENST00000256993:exon24:c.C2602A:p.P868T,ENSG00000134571:ENST00000399249:exon25:c.C2605A:p.P869T,ENSG00000134571:ENST00000545968:exon26:c.C2605A:p.P869T		P	0,00188
MYBPC3	11_47357560_GA_GGA	ENSG00000134571:ENST00000256993:exon24:c.2601_2602TCC,ENSG00000134571:ENST00000399249:exon25:c.2604_2605TCC,ENSG00000134571:ENST00000545968:exon26:c.2604_2605TCC		N	0,00063
MYBPC3	11_47357561_A_-	ENSG00000134571:ENST00000256993:exon24:c.2601delT:p.G867fs,ENSG00000134571:ENST00000399249:exon25:c.2604delT:p.G868fs,ENSG00000134571:ENST00000545968:exon26:c.2604delT:p.G868fs		P	0,00253
MYBPC3	11_47357563_C_G	ENST00000256993:exon25:c.2600-1G>C,ENST00000399249:exon26:c.2603-1G>C,ENST00000545968:exon27:c.2603-1G>C,ENST00000544791:exon27:c.2535-1G>C		L	0,00062
MYBPC3	11_47357564_T_C	ENST00000256993:exon25:c.2600-2A>G,ENST00000399249:exon26:c.2603-2A>G,ENST00000545968:exon27:c.2603-2A>G,ENST00000544791:exon27:c.2535-2A>G		L	0,00125

MYBPC3	11_47358987_C_-	ENSG00000134571:ENST00000256993:exon23:c.2554delG:p.G852fs,ENSG00000134571:ENST00000399249:exon24:c.2557delG:p.G853fs,ENSG00000134571:ENST00000545968:exon25:c.2557delG:p.G853fs		P	0,00080
MYBPC3	11_47358999_C_-	ENSG00000134571:ENST00000256993:exon23:c.2542delG:p.V848fs,ENSG00000134571:ENST00000399249:exon24:c.2545delG:p.V849fs,ENSG00000134571:ENST00000545968:exon25:c.2545delG:p.V849fs		L	0,00152
MYBPC3	11_47359000_C_-	ENSG00000134571:ENST00000256993:exon23:c.2541delG:p.A847fs,ENSG00000134571:ENST00000399249:exon24:c.2544delG:p.A848fs,ENSG00000134571:ENST00000545968:exon25:c.2544delG:p.A848fs		P	0,00151
MYBPC3	11_47359020_-_A	ENSG00000134571:ENST00000256993:exon23:c.2521_2522insT:p.Y841fs,ENSG00000134571:ENST00000399249:exon24:c.2524_2525insT:p.Y842fs,ENSG00000134571:ENST00000545968:exon25:c.2524_2525insT:p.Y842fs		L	0,00072
MYBPC3	11_47359032_-_C	ENSG00000134571:ENST00000256993:exon23:c.2509_2510insG:p.E837fs,ENSG00000134571:ENST00000399249:exon24:c.2512_2513insG:p.E838fs,ENSG00000134571:ENST00000545968:exon25:c.2512_2513insG:p.E838fs		L	0,00071
MYBPC3	11_47359047_C_T	ENSG00000134571:ENST00000256993:exon23:c.G2494A:p.A832T,ENSG00000134571:ENST00000399249:exon24:c.G2497A:p.A833T,ENSG00000134571:ENST00000545968:exon25:c.G2497A:p.A833T	rs199865688	D;P	0,00138
MYBPC3	11_47359085_C_T	ENSG00000134571:ENST00000256993:exon23:c.G2456A:p.R819Q,ENSG00000134571:ENST00000399249:exon24:c.G2459A:p.R820Q,ENSG00000134571:ENST00000545968:exon25:c.G2459A:p.R820Q	rs2856655	D;P	0,00142
MYBPC3	11_47359095_G_A	ENSG00000134571:ENST00000256993:exon23:c.C2446T:p.R816W,ENSG00000134571:ENST00000399249:exon24:c.C2449T:p.R817W,ENSG00000134571:ENST00000545968:exon25:c.C2449T:p.R817W		S	0,00071
MYBPC3	11_47359109_T_C	ENSG00000134571:ENST00000256993:exon23:c.A2432G:p.K811R,ENSG00000134571:ENST00000399249:exon24:c.A2435G:p.K812R,ENSG00000134571:ENST00000545968:exon25:c.A2435G:p.K812R		N	0,00072
MYBPC3	11_47359115_C_T	ENSG00000134571:ENST00000256993:exon23:c.G2426A:p.R809H,ENSG00000134571:ENST00000399249:exon24:c.G2429A:p.R810H,ENSG00000134571:ENST00000545968:exon25:c.G2429A:p.R810H		P	0,00218
MYBPC3	11_47359116_G_A	ENSG00000134571:ENST00000256993:exon23:c.C2425T:p.R809C,ENSG00000134571:ENST00000399249:exon24:c.C2428T:p.R810C,ENSG00000134571:ENST00000545968:exon25:c.C2428T:p.R810C		S	0,00074
MYBPC3	11_47359251_C_G	ENSG00000134571:ENST00000256993:exon22:c.G2400C:p.Q800H,ENSG00000134571:ENST00000399249:exon23:c.G2403C:p.Q801H,ENSG00000134571:ENST00000544791:exon24:c.G2403C:p.Q801H,ENSG00000134571:ENST00000545968:exon24:c.G2403C:p.Q801H		N	0,00073

MYBPC3	11_47359280_-_C	ENSG00000134571:ENST00000256993:exon22:c.2371_2372insG:p.W791fs,ENSG00000134571:ENST00000399249:exon23:c.2374_2375insG:p.W792fs,ENSG00000134571:ENST00000544791:exon24:c.2374_2375insG:p.W792fs,ENSG00000134571:ENST00000545968:exon24:c.2374_2375insG:p.W792fs		P	0,00645
MYBPC3	11_47359281_-_C	ENSG00000134571:ENST00000256993:exon22:c.2370_2371insG:p.Q790fs,ENSG00000134571:ENST00000399249:exon23:c.2373_2374insG:p.Q791fs,ENSG00000134571:ENST00000544791:exon24:c.2373_2374insG:p.Q791fs,ENSG00000134571:ENST00000545968:exon24:c.2373_2374insG:p.Q791fs		P	0,00646
MYBPC3	11_47359347_T_C	ENST00000256993:exon23:c.2306-2A>G,ENST00000399249:exon24:c.2309-2A>G,ENST00000545968:exon25:c.2309-2A>G,ENST00000544791:exon25:c.2309-2A>G		P	0,00064
MYBPC3	11_47360071_C_T	ENSG00000134571:ENST00000256993:exon21:c.G2305A:p.D769N,ENSG00000134571:ENST00000399249:exon22:c.G2308A:p.D770N,ENSG00000134571:ENST00000544791:exon23:c.G2308A:p.D770N,ENSG00000134571:ENST00000545968:exon23:c.G2308A:p.D770N	rs36211723	D;P	0,00064
MYBPC3	11_47360104_C_T	ENSG00000134571:ENST00000256993:exon21:c.G2272A:p.E758K,ENSG00000134571:ENST00000399249:exon22:c.G2275A:p.E759K,ENSG00000134571:ENST00000544791:exon23:c.G2275A:p.E759K,ENSG00000134571:ENST00000545968:exon23:c.G2275A:p.E759K		S	0,00062
MYBPC3	11_47360110_C_T	ENSG00000134571:ENST00000256993:exon21:c.G2266A:p.V756M,ENSG00000134571:ENST00000399249:exon22:c.G2269A:p.V757M,ENSG00000134571:ENST00000544791:exon23:c.G2269A:p.V757M,ENSG00000134571:ENST00000545968:exon23:c.G2269A:p.V757M		P	0,00061
MYBPC3	11_47360200_C_T	ENSG00000134571:ENST00000256993:exon21:c.G2176A:p.V726M,ENSG00000134571:ENST00000399249:exon22:c.G2179A:p.V727M,ENSG00000134571:ENST00000544791:exon23:c.G2179A:p.V727M,ENSG00000134571:ENST00000545968:exon23:c.G2179A:p.V727M		S	0,00066
MYBPC3	11_47360235_G_A	ENST00000256993:exon22:c.2146-5C>T,ENST00000399249:exon23:c.2149-5C>T,ENST00000545968:exon24:c.2149-5C>T,ENST00000544791:exon24:c.2149-5C>T	rs36211722	D;P	0,00139
MYBPC3	11_47360927_G_-	ENSG00000134571:ENST00000256993:exon20:c.2093delC:p.P698fs,ENSG00000134571:ENST00000399249:exon21:c.2096delC:p.P699fs,ENSG00000134571:ENST00000544791:exon22:c.2096delC:p.P699fs,ENSG00000134571:ENST00000545968:exon22:c.2096delC:p.P699fs		P	0,00173
MYBPC3	11_47360930_G_-	ENSG00000134571:ENST00000256993:exon20:c.2090delC:p.A697fs,ENSG00000134571:ENST00000399249:exon21:c.2093delC:p.A698fs,ENSG00000134571:ENST00000544791:exon22:c.2093delC:p.A698fs,ENSG00000134571:ENST00000545968:exon22:c.2093delC:p.A698fs		P	0,00173
MYBPC3	11_47361309_G_A	ENSG00000134571:ENST00000256993:exon19:c.C1957T:p.R653C,ENSG00000134571:ENST00000399249:exon20:c.C1960T:p.R654C,ENSG00000134571:ENST00000544791:exon21:c.C1960T:p.R654C,ENSG00000134571:ENST00000545968:exon21:c.C1960T:p.R654C		P	0,00059
MYBPC3	11_47361343_T_C	ENST00000256993:exon20:c.1925-2A>G,ENST00000399249:exon21:c.1928-2A>G,ENST00000545968:exon22:c.1928-2A>G,ENST00000544791:exon22:c.1928-2A>G		P	0,00061

MYBPC3	11_47362731_C_T	ENSG00000134571:ENST00000256993:exon17:c.G1852A:p.E618K,ENSG00000134571:ENST00000399249:exon18:c.G1855A:p.E619K,ENSG00000134571:ENST00000544791:exon19:c.G1855A:p.E619K,ENSG00000134571:ENST00000545968:exon19:c.G1855A:p.E619K	rs200352299	D;P	0,00065
MYBPC3	11_47363546_C_T	ENSG00000134571:ENST00000256993:exon16:c.G1783A:p.G595R,ENSG00000134571:ENST00000399249:exon17:c.G1786A:p.G596R,ENSG00000134571:ENST00000544791:exon18:c.G1786A:p.G596R,ENSG00000134571:ENST00000545968:exon18:c.G1786A:p.G596R	rs199728019	D;P	0,00116
MYBPC3	11_47363567_G_A	ENSG00000134571:ENST00000256993:exon16:c.C1762T:p.R588C,ENSG00000134571:ENST00000399249:exon17:c.C1765T:p.R589C,ENSG00000134571:ENST00000544791:exon18:c.C1765T:p.R589C,ENSG00000134571:ENST00000545968:exon18:c.C1765T:p.R589C		S	0,00058
MYBPC3	11_47363704_T_-	ENSG00000134571:ENST00000256993:exon16:c.1625delA:p.K542fs,ENSG00000134571:ENST00000399249:exon17:c.1628delA:p.K543fs,ENSG00000134571:ENST00000544791:exon18:c.1628delA:p.K543fs,ENSG00000134571:ENST00000545968:exon18:c.1628delA:p.K543fs		L	0,00058
MYBPC3	11_47364125_T_A	ENST00000256993:exon16:c.1621+4A>T,ENST00000399249:exon17:c.1624+4A>T,ENST00000545968:exon18:c.1624+4A>T,ENST00000544791:exon18:c.1624+4A>T		P	0,01346
MYBPC3	11_47364129_C_A	ENSG00000134571:ENST00000256993:exon15:c.G1621T:p.E541X,ENSG00000134571:ENST00000399249:exon16:c.G1624T:p.E542X,ENSG00000134571:ENST00000544791:exon17:c.G1624T:p.E542X,ENSG00000134571:ENST00000545968:exon17:c.G1624T:p.E542X		L	0,00090
MYBPC3	11_47364129_C_G	ENSG00000134571:ENST00000256993:exon15:c.G1621C:p.E541Q,ENSG00000134571:ENST00000399249:exon16:c.G1624C:p.E542Q,ENSG00000134571:ENST00000544791:exon17:c.G1624C:p.E542Q,ENSG00000134571:ENST00000545968:exon17:c.G1624C:p.E542Q	rs121909374	D;P	0,00269
MYBPC3	11_47364162_C_G	ENSG00000134571:ENST00000256993:exon15:c.G1588C:p.G530R,ENSG00000134571:ENST00000399249:exon16:c.G1591C:p.G531R,ENSG00000134571:ENST00000544791:exon17:c.G1591C:p.G531R,ENSG00000134571:ENST00000545968:exon17:c.G1591C:p.G531R		P	0,00083
MYBPC3	11_47364173_A_G	ENSG00000134571:ENST00000256993:exon15:c.T1577C:p.L526P,ENSG00000134571:ENST00000399249:exon16:c.T1580C:p.L527P,ENSG00000134571:ENST00000544791:exon17:c.T1580C:p.L527P,ENSG00000134571:ENST00000545968:exon17:c.T1580C:p.L527P		P	0,00078
MYBPC3	11_47364180_A_G	ENSG00000134571:ENST00000256993:exon15:c.T1570C:p.Y524H,ENSG00000134571:ENST00000399249:exon16:c.T1573C:p.Y525H,ENSG00000134571:ENST00000544791:exon17:c.T1573C:p.Y525H,ENSG00000134571:ENST00000545968:exon17:c.T1573C:p.Y525H		S	0,00076
MYBPC3	11_47364204_C_G	ENSG00000134571:ENST00000256993:exon15:c.G1546C:p.A516P,ENSG00000134571:ENST00000399249:exon16:c.G1549C:p.A517P,ENSG00000134571:ENST00000544791:exon17:c.G1549C:p.A517P,ENSG00000134571:ENST00000545968:exon17:c.G1549C:p.A517P		N	0,00071
MYBPC3	11_47364249_G_A	ENSG00000134571:ENST00000256993:exon15:c.C1501T:p.R501W,ENSG00000134571:ENST00000399249:exon16:c.C1504T:p.R502W,ENSG00000134571:ENST00000544791:exon17:c.C1504T:p.R502W,ENSG00000134571:ENST00000545968:exon17:c.C1504T:p.R502W		P	0,01038

MYBPC3	11_47364269_C_T	ENSG00000134571:ENST00000256993:exon15:c.G1481A:p.R494Q,ENSG00000134571:ENST00000399249:exon16:c.G1484A:p.R495Q,ENSG00000134571:ENST00000544791:exon17:c.G1484A:p.R495Q,ENSG00000134571:ENST00000545968:exon17:c.G1484A:p.R495Q	rs200411226	D;P	0,00319
MYBPC3	11_47364270_G_A	ENSG00000134571:ENST00000256993:exon15:c.C1480T:p.R494W,ENSG00000134571:ENST00000399249:exon16:c.C1483T:p.R495W,ENSG00000134571:ENST00000544791:exon17:c.C1483T:p.R495W,ENSG00000134571:ENST00000545968:exon17:c.C1483T:p.R495W		P	0,00064
MYBPC3	11_47364270_G_C	ENSG00000134571:ENST00000256993:exon15:c.C1480G:p.R494G,ENSG00000134571:ENST00000399249:exon16:c.C1483G:p.R495G,ENSG00000134571:ENST00000544791:exon17:c.C1483G:p.R495G,ENSG00000134571:ENST00000545968:exon17:c.C1483G:p.R495G		N	0,00256
MYBPC3	11_47364282_C_T	ENSG00000134571:ENST00000256993:exon15:c.G1468A:p.V490M,ENSG00000134571:ENST00000399249:exon16:c.G1471A:p.V491M,ENSG00000134571:ENST00000544791:exon17:c.G1471A:p.V491M,ENSG00000134571:ENST00000545968:exon17:c.G1471A:p.V491M		P	0,00064
MYBPC3	11_47364376_C_G	ENST00000256993:exon15:c.1454+5G>C,ENST00000399249:exon16:c.1457+5G>C,ENST00000545968:exon17:c.1457+5G>C,ENST00000544791:exon17:c.1457+5G>C		L	0,00060
MYBPC3	11_47364377_T_C	ENST00000256993:exon15:c.1454+4A>G,ENST00000399249:exon16:c.1457+4A>G,ENST00000545968:exon17:c.1457+4A>G,ENST00000544791:exon17:c.1457+4A>G		L	0,00120
MYBPC3	11_47364393_G_A	ENSG00000134571:ENST00000256993:exon14:c.C1442T:p.A481V,ENSG00000134571:ENST00000399249:exon15:c.C1445T:p.A482V,ENSG00000134571:ENST00000544791:exon16:c.C1445T:p.A482V,ENSG00000134571:ENST00000545968:exon16:c.C1445T:p.A482V		S	0,00060
MYBPC3	11_47364479_A_-	ENSG00000134571:ENST00000256993:exon14:c.1356delT:p.P452fs,ENSG00000134571:ENST00000399249:exon15:c.1359delT:p.P453fs,ENSG00000134571:ENST00000544791:exon16:c.1359delT:p.P453fs,ENSG00000134571:ENST00000545968:exon16:c.1359delT:p.P453fs		L	0,00061
MYBPC3	11_47364620_G_A	ENSG00000134571:ENST00000256993:exon13:c.C1300T:p.Q434X,ENSG00000134571:ENST00000399249:exon14:c.C1303T:p.Q435X,ENSG00000134571:ENST00000544791:exon15:c.C1303T:p.Q435X,ENSG00000134571:ENST00000545968:exon15:c.C1303T:p.Q435X		L	0,00063
MYBPC3	11_47364621_G_C	ENSG00000134571:ENST00000256993:exon13:c.C1299G:p.Y433X,ENSG00000134571:ENST00000399249:exon14:c.C1302G:p.Y434X,ENSG00000134571:ENST00000544791:exon15:c.C1302G:p.Y434X,ENSG00000134571:ENST00000545968:exon15:c.C1302G:p.Y434X		L	0,00063
MYBPC3	11_47364621_G_T	ENSG00000134571:ENST00000256993:exon13:c.C1299A:p.Y433X,ENSG00000134571:ENST00000399249:exon14:c.C1302A:p.Y434X,ENSG00000134571:ENST00000544791:exon15:c.C1302A:p.Y434X,ENSG00000134571:ENST00000545968:exon15:c.C1302A:p.Y434X		L	0,00063
MYBPC3	11_47364650_G_A	ENSG00000134571:ENST00000256993:exon13:c.C1270T:p.Q424X,ENSG00000134571:ENST00000399249:exon14:c.C1273T:p.Q425X,ENSG00000134571:ENST00000544791:exon15:c.C1273T:p.Q425X,ENSG00000134571:ENST00000545968:exon15:c.C1273T:p.Q425X		P	0,00063
MYBPC3	11_47364677_C_T	ENSG00000134571:ENST00000256993:exon13:c.G1243A:p.G415S,ENSG00000134571:ENST00000399249:exon14:c.G1246A:p.G416S,ENSG00000134571:ENST00000544791:exon15:c.G1246A:p.G416S,ENSG00000134571:ENST00000545968:exon15:c.G1246A:p.G416S		P	0,00066

MYBPC3	11_47364686_-_A	ENSG00000134571:ENST00000256993:exon13:c.1234_1235insT:p.E412_S413delinsX,ENSG00000134571:ENST00000399249:exon14:c.1237_1238insT:p.E413_S414delinsX,ENSG00000134571:ENST00000544791:exon15:c.1237_1238insT:p.E413_S414delinsX,ENSG00000134571:ENST00000545968:exon15:c.1237_1238insT:p.E413_S414delinsX		L	0,00137
MYBPC3	11_47364689_-_A	ENSG00000134571:ENST00000256993:exon13:c.1231_1232insT:p.F411fs,ENSG00000134571:ENST00000399249:exon14:c.1234_1235insT:p.F412fs,ENSG00000134571:ENST00000544791:exon15:c.1234_1235insT:p.F412fs,ENSG00000134571:ENST00000545968:exon15:c.1234_1235insT:p.F412fs		P	0,00137
MYBPC3	11_47364698_T_C	ENST00000256993:exon14:c.1224-2A>G,ENST00000399249:exon15:c.1227-2A>G,ENST00000545968:exon16:c.1227-2A>G,ENST00000544791:exon16:c.1227-2A>G		L	0,00070
MYBPC3	11_47365143_C_T	ENSG00000134571:ENST00000256993:exon12:c.G1123A:p.V375M,ENSG00000134571:ENST00000399249:exon12:c.G1123A:p.V375M,ENSG00000134571:ENST00000544791:exon13:c.G1123A:p.V375M,ENSG00000134571:ENST00000545968:exon13:c.G1123A:p.V375M		S	0,00095
MYBPC3	11_47367757_C_A	ENST00000256993:exon12:c.1090+1G>T,ENST00000399249:exon12:c.1090+1G>T,ENST00000545968:exon13:c.1090+1G>T,ENST00000544791:exon13:c.1090+1G>T		P	0,00231
MYBPC3	11_47367757_C_T	ENST00000256993:exon12:c.1090+1G>A,ENST00000399249:exon12:c.1090+1G>A,ENST00000545968:exon13:c.1090+1G>A,ENST00000544791:exon13:c.1090+1G>A		P	0,00058
MYBPC3	11_47367764_T_C	ENSG00000134571:ENST00000256993:exon11:c.A1084G:p.S362G,ENSG00000134571:ENST00000399249:exon11:c.A1084G:p.S362G,ENSG00000134571:ENST00000544791:exon12:c.A1084G:p.S362G,ENSG00000134571:ENST00000545968:exon12:c.A1084G:p.S362G		S	0,00058
MYBPC3	11_47367776_C_T	ENSG00000134571:ENST00000256993:exon11:c.G1072A:p.D358N,ENSG00000134571:ENST00000399249:exon11:c.G1072A:p.D358N,ENSG00000134571:ENST00000544791:exon12:c.G1072A:p.D358N,ENSG00000134571:ENST00000545968:exon12:c.G1072A:p.D358N		N	0,00058
MYBPC3	11_47367822_GACGCCGT_-	ENSG00000134571:ENST00000256993:exon11:c.1019_1026del:p.340_342del,ENSG00000134571:ENST00000399249:exon11:c.1019_1026del:p.340_342del,ENSG00000134571:ENST00000544791:exon12:c.1019_1026del:p.340_342del,ENSG00000134571:ENST00000545968:exon12:c.1019_1026del:p.340_342del		L	0,00058
MYBPC3	11_47367848_C_T	ENSG00000134571:ENST00000256993:exon11:c.G1000A:p.E334K,ENSG00000134571:ENST00000399249:exon11:c.G1000A:p.E334K,ENSG00000134571:ENST00000544791:exon12:c.G1000A:p.E334K,ENSG00000134571:ENST00000545968:exon12:c.G1000A:p.E334K		P	0,00061
MYBPC3	11_47367849_G_C	ENSG00000134571:ENST00000256993:exon11:c.C999G:p.Y333X,ENSG00000134571:ENST00000399249:exon11:c.C999G:p.Y333X,ENSG00000134571:ENST00000544791:exon12:c.C999G:p.Y333X,ENSG00000134571:ENST00000545968:exon12:c.C999G:p.Y333X		L	0,00061
MYBPC3	11_47367887_C_T	ENSG00000134571:ENST00000256993:exon11:c.G961A:p.V321M,ENSG00000134571:ENST00000399249:exon11:c.G961A:p.V321M,ENSG00000134571:ENST00000544791:exon12:c.G961A:p.V321M,ENSG00000134571:ENST00000545968:exon12:c.G961A:p.V321M	rs200119454	D;P	0,00187
MYBPC3	11_47367923_T_C	ENST00000256993:exon12:c.927-2A>G,ENST00000399249:exon12:c.927-2A>G,ENST00000545968:exon13:c.927-2A>G,ENST00000544791:exon13:c.927-2A>G		P	0,00132

MYBPC3	11_47368187_C_T	ENSG00000134571:ENST00000256993:exon10:c.G917A:p.R306Q,ENSG00000134571:ENST00000399249:exon10:c.G917A:p.R306Q,ENSG00000134571:ENST00000544791:exon11:c.G917A:p.R306Q,ENSG00000134571:ENST00000545968:exon11:c.G917A:p.R306Q		N	0,00059
MYBPC3	11_47368202_C_T	ENST00000256993:exon11:c.906-4G>A,ENST00000399249:exon11:c.906-4G>A		L	0,00060
MYBPC3	11_47369023_G_A	ENSG00000134571:ENST00000256993:exon9:c.C859T:p.H287Y,ENSG00000134571:ENST00000399249:exon9:c.C859T:p.H287Y,ENSG00000134571:ENST00000544791:exon9:c.C859T:p.H287Y,ENSG00000134571:ENST00000545968:exon9:c.C859T:p.H287Y		P	0,00066
MYBPC3	11_47369406_A_G	ENST00000256993:exon8:c.821+2T>C,ENST00000399249:exon8:c.821+2T>C,ENST00000545968:exon8:c.821+2T>C,ENST00000544791:exon8:c.821+2T>C		P	0,00087
MYBPC3	11_47369975_C_T	ENSG00000134571:ENST00000256993:exon6:c.G772A:p.E258K,ENSG00000134571:ENST00000399249:exon6:c.G772A:p.E258K,ENSG00000134571:ENST00000544791:exon6:c.G772A:p.E258K,ENSG00000134571:ENST00000545968:exon6:c.G772A:p.E258K		P	0,00616
MYBPC3	11_47370037_T_G	ENSG00000134571:ENST00000256993:exon6:c.A710C:p.Y237S,ENSG00000134571:ENST00000399249:exon6:c.A710C:p.Y237S,ENSG00000134571:ENST00000544791:exon6:c.A710C:p.Y237S,ENSG00000134571:ENST00000545968:exon6:c.A710C:p.Y237S		P	0,00140
MYBPC3	11_47370092_C_G	ENSG00000134571:ENST00000256993:exon6:c.G655C:p.V219L,ENSG00000134571:ENST00000399249:exon6:c.G655C:p.V219L,ENSG00000134571:ENST00000544791:exon6:c.G655C:p.V219L,ENSG00000134571:ENST00000545968:exon6:c.G655C:p.V219L		P	0,00442
MYBPC3	11_47371330_T_C	ENSG00000134571:ENST00000256993:exon5:c.A649G:p.S217G,ENSG00000134571:ENST00000399249:exon5:c.A649G:p.S217G,ENSG00000134571:ENST00000544791:exon5:c.A649G:p.S217G,ENSG00000134571:ENST00000545968:exon5:c.A649G:p.S217G	rs138753870	D;P	0,00067
MYBPC3	11_47371366_G_A	ENSG00000134571:ENST00000256993:exon5:c.C613T:p.Q205X,ENSG00000134571:ENST00000399249:exon5:c.C613T:p.Q205X,ENSG00000134571:ENST00000544791:exon5:c.C613T:p.Q205X,ENSG00000134571:ENST00000545968:exon5:c.C613T:p.Q205X		L	0,00068
MYBPC3	11_47371390_CCCATTTG_-	ENSG00000134571:ENST00000256993:exon5:c.582_589del:p.194_197del,ENSG00000134571:ENST00000399249:exon5:c.582_589del:p.194_197del,ENSG00000134571:ENST00000544791:exon5:c.582_589del:p.194_197del,ENSG00000134571:ENST00000545968:exon5:c.582_589del:p.194_197del		L	0,00071
MYBPC3	11_47371560_C_T	ENST00000256993:exon5:c.505+5G>A,ENST00000399249:exon5:c.505+5G>A,ENST00000545968:exon5:c.505+5G>A,ENST00000544791:exon5:c.505+5G>A		L	0,00096
MYBPC3	11_47372107_CA_-	ENSG00000134571:ENST00000256993:exon3:c.351_352del:p.117_118del,ENSG00000134571:ENST00000399249:exon3:c.351_352del:p.117_118del,ENSG00000134571:ENST00000544791:exon3:c.351_352del:p.117_118del,ENSG00000134571:ENST00000545968:exon3:c.351_352del:p.117_118del		L	0,00182
MYBPC3	11_47372859_C_T	ENSG00000134571:ENST00000256993:exon2:c.G223A:p.D75N,ENSG00000134571:ENST00000399249:exon2:c.G223A:p.D75N,ENSG00000134571:ENST00000544791:exon2:c.G223A:p.D75N,ENSG00000134571:ENST00000545968:exon2:c.G223A:p.D75N		P	0,00082

MYBPC3	11_47372866_-_CC	ENSG00000134571:ENST00000256993:exon2:c.216_217insGG:p.G72fs,ENSG00000134571:ENST00000399249:exon2:c.216_217insGG:p.G72fs,ENSG00000134571:ENST00000544791:exon2:c.216_217insGG:p.G72fs,ENSG00000134571:ENST00000545968:exon2:c.216_217insGG:p.G72fs		L	0,00081
MYBPC3	11_47372898_TGCCCTCTGTG_-	ENSG00000134571:ENST00000256993:exon2:c.174_184del:p.58_62del,ENSG00000134571:ENST00000399249:exon2:c.174_184del:p.58_62del,ENSG00000134571:ENST00000544791:exon2:c.174_184del:p.58_62del,ENSG00000134571:ENST00000545968:exon2:c.174_184del:p.58_62del		P	0,00319
MYBPC3	11_47372956_C_T	ENSG00000134571:ENST00000256993:exon2:c.G126A:p.W42X,ENSG00000134571:ENST00000399249:exon2:c.G126A:p.W42X,ENSG00000134571:ENST00000544791:exon2:c.G126A:p.W42X,ENSG00000134571:ENST00000545968:exon2:c.G126A:p.W42X		L	0,00078
MYBPC3	11_47372961_-_G	ENSG00000134571:ENST00000256993:exon2:c.121_122insC:p.R41fs,ENSG00000134571:ENST00000399249:exon2:c.121_122insC:p.R41fs,ENSG00000134571:ENST00000544791:exon2:c.121_122insC:p.R41fs,ENSG00000134571:ENST00000545968:exon2:c.121_122insC:p.R41fs		L	0,00077
MYBPC3	11_47374186_C_G	ENSG00000134571:ENST00000256993:exon1:c.G13C:p.G5R,ENSG00000134571:ENST00000399249:exon1:c.G13C:p.G5R,ENSG00000134571:ENST00000544791:exon1:c.G13C:p.G5R,ENSG00000134571:ENST00000545968:exon1:c.G13C:p.G5R	rs201278114	D;P	0,00117
MYH6	14_23852451_G_T	ENSG00000197616:ENST00000356287:exon36:c.C5644A:p.R1882S,ENSG00000197616:ENST00000405093:exon37:c.C5644A:p.R1882S		S	0,00058
MYH6	14_23852501_C_T	ENSG00000197616:ENST00000356287:exon36:c.G5594A:p.R1865Q,ENSG00000197616:ENST00000405093:exon37:c.G5594A:p.R1865Q	rs138720701	D;P	0,00059
MYH6	14_23852520_C_T	ENSG00000197616:ENST00000356287:exon36:c.G5575A:p.D1859N,ENSG00000197616:ENST00000405093:exon37:c.G5575A:p.D1859N		S	0,00069
MYH6	14_23853739_C_T	ENSG00000197616:ENST00000356287:exon35:c.G5477A:p.G1826D,ENSG00000197616:ENST00000405093:exon36:c.G5477A:p.G1826D	rs200260229	D	0,00119
MYH6	14_23853740_C_T	ENSG00000197616:ENST00000356287:exon35:c.G5476A:p.G1826S,ENSG00000197616:ENST00000405093:exon36:c.G5476A:p.G1826S	rs202141059	D	0,00119
MYH6	14_23853806_G_T	ENSG00000197616:ENST00000356287:exon35:c.C5410A:p.Q1804K,ENSG00000197616:ENST00000405093:exon36:c.C5410A:p.Q1804K	rs144571463	D	0,00060
MYH6	14_23854220_TCT_-	ENSG00000197616:ENST00000356287:exon34:c.5192_5194del:p.1731_1732del,ENSG00000197616:ENST00000405093:exon35:c.5192_5194del:p.1731_1732del		N	0,00057
MYH6	14_23855136_C_A	ENST00000405093:exon35:c.5163+1G>T,ENST00000356287:exon34:c.5163+1G>T		L	0,00058
MYH6	14_23855160_G_A	ENSG00000197616:ENST00000356287:exon33:c.C5140T:p.R1714W,ENSG00000197616:ENST00000405093:exon34:c.C5140T:p.R1714W	rs140651265	D;S	0,00058
MYH6	14_23855228_C_T	ENSG00000197616:ENST00000356287:exon33:c.G5072A:p.R1691H,ENSG00000197616:ENST00000405093:exon34:c.G5072A:p.R1691H		S	0,00060
MYH6	14_23855609_T_C	ENSG00000197616:ENST00000356287:exon32:c.A4874G:p.N1625S,ENSG00000197616:ENST00000405093:exon33:c.A4874G:p.N1625S		S	0,00058

MYH6	14_23855779_G_T	ENSG00000197616:ENST00000356287:exon32:c.C4704A:p.N1568K,ENSG00000197616:ENST00000405093:exon33:c.C4704A:p.N1568K	rs149771264	D;S	0,00058
MYH6	14_23856786_C_G	ENSG00000197616:ENST00000356287:exon31:c.G4602C:p.Q1534H,ENSG00000197616:ENST00000405093:exon32:c.G4602C:p.Q1534H	rs199600772	D;S	0,00057
MYH6	14_23856987_C_T	ENSG00000197616:ENST00000356287:exon30:c.G4505A:p.R1502Q,ENSG00000197616:ENST00000405093:exon31:c.G4505A:p.R1502Q	rs199936506	D	0,00115
MYH6	14_23857371_A_T	ENSG00000197616:ENST00000356287:exon29:c.T4352A:p.F1451Y,ENSG00000197616:ENST00000405093:exon30:c.T4352A:p.F1451Y		N	0,00058
MYH6	14_23857395_G_T	ENSG00000197616:ENST00000356287:exon29:c.C4328A:p.A1443D,ENSG00000197616:ENST00000405093:exon30:c.C4328A:p.A1443D		P	0,00058
MYH6	14_23857530_C_T	ENSG00000197616:ENST00000356287:exon29:c.G4193A:p.R1398Q,ENSG00000197616:ENST00000405093:exon30:c.G4193A:p.R1398Q	rs150815925	D	0,00058
MYH6	14_23858107_G_A	ENSG00000197616:ENST00000356287:exon28:c.C4136T:p.T1379M,ENSG00000197616:ENST00000405093:exon29:c.C4136T:p.T1379M	rs145611185	D;P	0,00058
MYH6	14_23858161_C_T	ENSG00000197616:ENST00000356287:exon28:c.G4082A:p.R1361H,ENSG00000197616:ENST00000405093:exon29:c.G4082A:p.R1361H		S	0,00058
MYH6	14_23858648_G_A	ENSG00000197616:ENST00000356287:exon27:c.C3932T:p.T1311I,ENSG00000197616:ENST00000405093:exon28:c.C3932T:p.T1311I		S	0,00058
MYH6	14_23858687_G_A	ENSG00000197616:ENST00000356287:exon27:c.C3893T:p.A1298V,ENSG00000197616:ENST00000405093:exon28:c.C3893T:p.A1298V		N	0,00058
MYH6	14_23858720_C_T	ENSG00000197616:ENST00000356287:exon27:c.G3860A:p.G1287E,ENSG00000197616:ENST00000405093:exon28:c.G3860A:p.G1287E		N	0,00058
MYH6	14_23859370_C_T	ENSG00000197616:ENST00000356287:exon25:c.G3628A:p.D1210N,ENSG00000197616:ENST00000405093:exon26:c.G3628A:p.D1210N		S	0,00065
MYH6	14_23859484_C_T	ENSG00000197616:ENST00000356287:exon25:c.G3514A:p.E1172K,ENSG00000197616:ENST00000405093:exon26:c.G3514A:p.E1172K		S	0,00092
MYH6	14_23859571_G_A	ENSG00000197616:ENST00000356287:exon25:c.C3427T:p.R1143W,ENSG00000197616:ENST00000405093:exon26:c.C3427T:p.R1143W		S	0,00093
MYH6	14_23859652_G_T	ENSG00000197616:ENST00000356287:exon25:c.C3346A:p.R1116S,ENSG00000197616:ENST00000405093:exon26:c.C3346A:p.R1116S		P	0,00080
MYH6	14_23861814_T_A	ENSG00000197616:ENST00000356287:exon24:c.A3299T:p.Q1100L,ENSG00000197616:ENST00000405093:exon25:c.A3299T:p.Q1100L		S	0,00057
MYH6	14_23862208_C_T	ENSG00000197616:ENST00000356287:exon23:c.G3164A:p.R1055Q,ENSG00000197616:ENST00000405093:exon24:c.G3164A:p.R1055Q		N	0,00057
MYH6	14_23862646_C_A	ENSG00000197616:ENST00000356287:exon22:c.G3010T:p.A1004S,ENSG00000197616:ENST00000405093:exon23:c.G3010T:p.A1004S	rs143978652	D;P	0,00057

MYH6	14_23862870_C_T	ENST00000405093:exon23:c.2928+5G>A,ENST00000356287:exon22:c.2928+5G>A	rs28730772	D;L	0,00400
MYH6	14_23863087_G_A	ENSG00000197616:ENST00000356287:exon21:c.C2716T:p.R906C,ENSG00000197616:ENST00000405093:exon22:c.C2716T:p.R906C	rs143928061	D;S	0,00057
MYH6	14_23863348_G_A	ENSG00000197616:ENST00000356287:exon20:c.C2614T:p.R872C,ENSG00000197616:ENST00000405093:exon21:c.C2614T:p.R872C	rs201193346	D;S	0,00058
MYH6	14_23863384_G_A	ENSG00000197616:ENST00000356287:exon20:c.C2578T:p.R860C,ENSG00000197616:ENST00000405093:exon21:c.C2578T:p.R860C		S	0,00057
MYH6	14_23865497_G_A	ENSG00000197616:ENST00000356287:exon19:c.C2425T:p.R809C,ENSG00000197616:ENST00000405093:exon20:c.C2425T:p.R809C		S	0,00057
MYH6	14_23865569_G_A	ENSG00000197616:ENST00000356287:exon19:c.C2353T:p.R785C,ENSG00000197616:ENST00000405093:exon20:c.C2353T:p.R785C		S	0,00058
MYH6	14_23866275_G_T	ENSG00000197616:ENST00000356287:exon17:c.C2065A:p.P689T,ENSG00000197616:ENST00000405093:exon18:c.C2065A:p.P689T		N	0,00060
MYH6	14_23868065_T_G	ENSG00000197616:ENST00000356287:exon14:c.A1763C:p.D588A,ENSG00000197616:ENST00000405093:exon15:c.A1763C:p.D588A	rs142992009	D	0,00057
MYH6	14_23869462_C_T	ENST00000405093:exon15:c.1581+3G>A,ENST00000356287:exon14:c.1581+3G>A		L	0,00057
MYH6	14_23869967_C_A	ENSG00000197616:ENST00000356287:exon12:c.G1361T:p.R454L,ENSG00000197616:ENST00000405093:exon13:c.G1361T:p.R454L		S	0,00058
MYH6	14_23870001_G_A	ENSG00000197616:ENST00000356287:exon12:c.C1327T:p.R443C,ENSG00000197616:ENST00000405093:exon13:c.C1327T:p.R443C	rs182373896	D;S	0,00058
MYH6	14_23870012_C_T	ENSG00000197616:ENST00000356287:exon12:c.G1316A:p.W439X,ENSG00000197616:ENST00000405093:exon13:c.G1316A:p.W439X		L	0,00058
MYH6	14_23870034_C_G	ENSG00000197616:ENST00000356287:exon12:c.G1294C:p.V432L,ENSG00000197616:ENST00000405093:exon13:c.G1294C:p.V432L		N	0,00057
MYH6	14_23870151_A_C	ENSG00000197616:ENST00000356287:exon12:c.T1177G:p.S393A,ENSG00000197616:ENST00000405093:exon13:c.T1177G:p.S393A	rs199877580	D	0,00057
MYH6	14_23871758_C_G	ENSG00000197616:ENST00000356287:exon11:c.G1056C:p.K352N,ENSG00000197616:ENST00000405093:exon12:c.G1056C:p.K352N		S	0,00058
MYH6	14_23871766_C_T	ENSG00000197616:ENST00000356287:exon11:c.G1048A:p.V350I,ENSG00000197616:ENST00000405093:exon12:c.G1048A:p.V350I	rs200260629	D	0,00058
MYH6	14_23872631_A_T	ENSG00000197616:ENST00000356287:exon9:c.T824A:p.I275N,ENSG00000197616:ENST00000405093:exon10:c.T824A:p.I275N	rs201327273	D;P	0,00120
MYH6	14_23874309_G_A	ENSG00000197616:ENST00000356287:exon5:c.C530T:p.T177M,ENSG00000197616:ENST00000405093:exon6:c.C530T:p.T177M		S	0,00058
MYH6	14_23874525_C_T	ENSG00000197616:ENST00000356287:exon4:c.G409A:p.E137K,ENSG00000197616:ENST00000405093:exon5:c.G409A:p.E137K		N	0,00061

MYH6	14_23874889_C_T	ENSG00000197616:ENST00000356287:exon3:c.G292A:p.E98K,ENSG00000197616:ENST00000405093:exon4:c.G292A:p.E98K	rs140596256	D	0,00059
MYH6	14_23874936_G_A	ENSG00000197616:ENST00000356287:exon3:c.C245T:p.P82L,ENSG00000197616:ENST00000405093:exon4:c.C245T:p.P82L		S	0,00058
MYH6	14_23876352_C_G	ENSG00000197616:ENST00000356287:exon2:c.G81C:p.Q27H,ENSG00000197616:ENST00000405093:exon3:c.G81C:p.Q27H		N	0,00058
MYH6	14_23876363_G_T	ENSG00000197616:ENST00000356287:exon2:c.C70A:p.L24I,ENSG00000197616:ENST00000405093:exon3:c.C70A:p.L24I		N	0,00115
MYH6	14_23876785_C_T			L	0,00059
MYH7	14_23882979_T_A	ENSG00000092054:ENST00000355349:exon39:c.A5779T:p.I1927F		P	0,00058
MYH7	14_23883068_C_T	ENSG00000092054:ENST00000355349:exon39:c.G5690A:p.R1897H		S	0,00058
MYH7	14_23883217_G_A	ENSG00000092054:ENST00000355349:exon38:c.C5654T:p.A1885V		N	0,00059
MYH7	14_23884200_G_A	ENST00000355349:exon38:c.5559+4C>T		L	0,00058
MYH7	14_23884421_C_T	ENSG00000092054:ENST00000355349:exon37:c.G5342A:p.R1781H		P	0,00058
MYH7	14_23884469_A_T	ENSG00000092054:ENST00000355349:exon37:c.T5294A:p.M1765K		S	0,00058
MYH7	14_23884860_C_T	ENSG00000092054:ENST00000355349:exon35:c.G5135A:p.R1712Q	rs193922390	D;P	0,00063
MYH7	14_23885349_C_T	ENSG00000092054:ENST00000355349:exon34:c.G4817A:p.R1606H		N	0,00058
MYH7	14_23885502_T_C	ENSG00000092054:ENST00000355349:exon34:c.A4664G:p.E1555G		P	0,00058
MYH7	14_23886806_C_T	ENSG00000092054:ENST00000355349:exon31:c.G4259A:p.R1420Q		P	0,00058
MYH7	14_23886815_G_A	ENSG00000092054:ENST00000355349:exon31:c.C4250T:p.T1417I		S	0,00058
MYH7	14_23887522_C_T	ENSG00000092054:ENST00000355349:exon30:c.G4066A:p.E1356K		P	0,00296
MYH7	14_23887607_G_T	ENSG00000092054:ENST00000355349:exon30:c.C3981A:p.N1327K	rs141764279	D	0,00058
MYH7	14_23888775_T_C	ENSG00000092054:ENST00000355349:exon28:c.A3770G:p.N1257S		N	0,00058
MYH7	14_23889158_C_T	ENSG00000092054:ENST00000355349:exon27:c.G3622A:p.D1208N		S	0,00062
MYH7	14_23889187_T_C	ENSG00000092054:ENST00000355349:exon27:c.A3593G:p.D1198G		S	0,00070
MYH7	14_23892910_A_G	ENSG00000092054:ENST00000355349:exon24:c.T2945C:p.M982T	rs145532615	D;P	0,00057
MYH7	14_23893148_C_G	ENSG00000092054:ENST00000355349:exon23:c.G2890C:p.V964L	rs45496496	D;P	0,00344
MYH7	14_23893192_T_A	ENSG00000092054:ENST00000355349:exon23:c.A2846T:p.E949V		S	0,00057

MYH7	14_23893246_T_C	ENSG00000092054:ENST00000355349:exon23:c.A2792G:p.E931G		S	0,00057
MYH7	14_23893255_T_C	ENSG00000092054:ENST00000355349:exon23:c.A2783G:p.D928G		S	0,00057
MYH7	14_23893268_C_G	ENSG00000092054:ENST00000355349:exon23:c.G2770C:p.E924Q		P	0,00057
MYH7	14_23893268_C_T	ENSG00000092054:ENST00000355349:exon23:c.G2770A:p.E924K	rs121913628	D;P	0,00057
MYH7	14_23893316_G_C	ENSG00000092054:ENST00000355349:exon23:c.C2722G:p.L908V	rs121913631	D;P	0,00057
MYH7	14_23893321_T_C	ENSG00000092054:ENST00000355349:exon23:c.A2717G:p.D906G		P	0,00286
MYH7	14_23893357_T_C	ENSG00000092054:ENST00000355349:exon23:c.A2681G:p.E894G		P	0,00172
MYH7	14_23893988_T_G	ENSG00000092054:ENST00000355349:exon22:c.A2669C:p.Q890P		S	0,00060
MYH7	14_23894048_C_T	ENSG00000092054:ENST00000355349:exon22:c.G2609A:p.R870H	rs36211715	D;P	0,00293
MYH7	14_23894051_C_G	ENSG00000092054:ENST00000355349:exon22:c.G2606C:p.R869P		N	0,00117
MYH7	14_23894051_C_T	ENSG00000092054:ENST00000355349:exon22:c.G2606A:p.R869H	rs202141173	D;P	0,00058
MYH7	14_23894052_G_A	ENSG00000092054:ENST00000355349:exon22:c.C2605T:p.R869C		P	0,00117
MYH7	14_23894052_G_T	ENSG00000092054:ENST00000355349:exon22:c.C2605A:p.R869S		N	0,00058
MYH7	14_23894084_C_T	ENSG00000092054:ENST00000355349:exon22:c.G2573A:p.R858H	rs2856897	P	0,00058
MYH7	14_23894118_T_C	ENSG00000092054:ENST00000355349:exon22:c.A2539G:p.K847E		P	0,00116
MYH7	14_23894118_TCT_-	ENSG00000092054:ENST00000355349:exon22:c.2537_2539del:p.846_847del		N	0,00058
MYH7	14_23894123_C_T	ENSG00000092054:ENST00000355349:exon22:c.G2534A:p.R845K		N	0,00058
MYH7	14_23894193_T_A	ENSG00000092054:ENST00000355349:exon22:c.A2464T:p.M822L		P	0,00058
MYH7	14_23894525_C_T	ENSG00000092054:ENST00000355349:exon21:c.G2389A:p.A797T	rs3218716	D;P	0,00172
MYH7	14_23894539_G_T	ENSG00000092054:ENST00000355349:exon21:c.C2375A:p.S792Y		N	0,00058
MYH7	14_23894554_C_T	ENSG00000092054:ENST00000355349:exon21:c.G2360A:p.R787H		P	0,00173
MYH7	14_23894566_C_T	ENSG00000092054:ENST00000355349:exon21:c.G2348A:p.R783H		P	0,00058
MYH7	14_23894969_C_A	ENSG00000092054:ENST00000355349:exon20:c.G2221T:p.G741W		P	0,00057
MYH7	14_23894969_C_G	ENSG00000092054:ENST00000355349:exon20:c.G2221C:p.G741R	rs121913632	D;P	0,00057
MYH7	14_23895023_G_A	ENSG00000092054:ENST00000355349:exon20:c.C2167T:p.R723C	rs121913630	D;P	0,00230
MYH7	14_23895023_G_C	ENSG00000092054:ENST00000355349:exon20:c.C2167G:p.R723G		P	0,00057

MYH7	14_23895179_C_T	ENSG00000092054:ENST00000355349:exon19:c.G2156A:p.R719Q	rs121913641	D;P	0,00118
MYH7	14_23895180_G_A	ENSG00000092054:ENST00000355349:exon19:c.C2155T:p.R719W	rs121913637	D;P	0,00059
MYH7	14_23895243_C_G	ENSG00000092054:ENST00000355349:exon19:c.G2092C:p.V698L		N	0,00065
MYH7	14_23895254_C_T	ENSG00000092054:ENST00000355349:exon19:c.G2081A:p.R694H		P	0,00065
MYH7	14_23896042_C_T	ENSG00000092054:ENST00000355349:exon18:c.G1988A:p.R663H		P	0,00287
MYH7	14_23896043_G_A	ENSG00000092054:ENST00000355349:exon18:c.C1987T:p.R663C		P	0,00172
MYH7	14_23896812_A_T	ENSG00000092054:ENST00000355349:exon16:c.T1870A:p.Y624N		P	0,00057
MYH7	14_23896866_C_T	ENSG00000092054:ENST00000355349:exon16:c.G1816A:p.V606M	rs121913627	D;P	0,00229
MYH7	14_23897796_C_A	ENSG00000092054:ENST00000355349:exon15:c.G1491T:p.E497D		P	0,00057
MYH7	14_23897840_C_T	ENSG00000092054:ENST00000355349:exon15:c.G1447A:p.E483K	rs121913651	D;P	0,00057
MYH7	14_23897854_A_T	ENSG00000092054:ENST00000355349:exon15:c.T1433A:p.I478N		S	0,00057
MYH7	14_23898213_C_A	ENSG00000092054:ENST00000355349:exon14:c.G1358T:p.R453L		S	0,00057
MYH7	14_23898240_T_C	ENSG00000092054:ENST00000355349:exon14:c.A1331G:p.N444S		P	0,00057
MYH7	14_23898292_G_T	ENSG00000092054:ENST00000355349:exon14:c.C1279A:p.L427M		P	0,00230
MYH7	14_23898464_C_T	ENSG00000092054:ENST00000355349:exon13:c.G1231A:p.V411I		P	0,00057
MYH7	14_23898487_C_T	ENSG00000092054:ENST00000355349:exon13:c.G1208A:p.R403Q	rs121913624	D;P	0,00057
MYH7	14_23898488_G_A	ENSG00000092054:ENST00000355349:exon13:c.C1207T:p.R403W	rs3218714	D;P	0,00172
MYH7	14_23898551_C_A	ENSG00000092054:ENST00000355349:exon13:c.G1144T:p.D382Y		P	0,00115
MYH7	14_23899059_C_A	ENSG00000092054:ENST00000355349:exon12:c.G1063T:p.A355S		N	0,00115
MYH7	14_23899059_C_T	ENSG00000092054:ENST00000355349:exon12:c.G1063A:p.A355T		P	0,00229
MYH7	14_23899071_T_C	ENSG00000092054:ENST00000355349:exon12:c.A1051G:p.K351E		P	0,00115
MYH7	14_23899101_A_G	ENSG00000092054:ENST00000355349:exon12:c.T1021C:p.F341L		S	0,00057
MYH7	14_23899864_G_T	ENSG00000092054:ENST00000355349:exon11:c.C904A:p.L302M		S	0,00057
MYH7	14_23900635_A_G	ENSG00000092054:ENST00000355349:exon9:c.T788C:p.I263T		P	0,00057
MYH7	14_23900656_C_T	ENSG00000092054:ENST00000355349:exon9:c.G767A:p.G256E	rs121913633	D;P	0,00057
MYH7	14_23900677_C_T	ENSG00000092054:ENST00000355349:exon9:c.G746A:p.R249Q	rs3218713	D;P	0,00057

MYH7	14_23900811_C_T	ENSG00000092054:ENST00000355349:exon8:c.G715A:p.D239N		P	0,00058
MYH7	14_23901712_C_T	ENSG00000092054:ENST00000355349:exon6:c.G506A:p.R169K		N	0,00058
MYH7	14_23901922_C_T	ENSG00000092054:ENST00000355349:exon5:c.G428A:p.R143Q		P	0,00059
MYH7	14_23902293_G_T	ENSG00000092054:ENST00000355349:exon4:c.C345A:p.Y115X		L	0,00058
MYH7	14_23902340_C_T	ENSG00000092054:ENST00000355349:exon4:c.G298A:p.A100T		S	0,00058
MYH7	14_23902892_C_T	ENSG00000092054:ENST00000355349:exon3:c.G50A:p.R17H		S	0,00058
MYH7	14_23903456_G_A		rs45566639	D;L	0,00401
MYL2	12_111350901_T_G	ENSG00000111245:ENST00000548438:exon5:c.A359C:p.E120A,ENSG00000111245:ENST00000228841:exon6:c.A401C:p.E134A	rs143139258	D;S	0,00115
MYL2	12_111350926_G_C	ENSG00000111245:ENST00000548438:exon5:c.C334G:p.Q112E,ENSG00000111245:ENST00000550439:exon5:c.C319G:p.Q107E,ENSG00000111245:ENST00000228841:exon6:c.C376G:p.Q126E		S	0,00058
MYL2	12_111352004_C_G	ENSG00000111245:ENST00000548438:exon3:c.G218C:p.G73A,ENSG00000111245:ENST00000550439:exon3:c.G203C:p.G68A,ENSG00000111245:ENST00000228841:exon4:c.G260C:p.G87A		S	0,00058
MYL2	12_111352091_C_T	ENSG00000111245:ENST00000548438:exon3:c.G131A:p.R44Q,ENSG00000111245:ENST00000550439:exon3:c.G116A:p.R39Q,ENSG00000111245:ENST00000228841:exon4:c.G173A:p.R58Q	rs104894369	D;P	0,00057
MYL2	12_111353547_G_T	ENSG00000111245:ENST00000550439:exon2:c.C84A:p.N28K,ENSG00000111245:ENST00000228841:exon3:c.C141A:p.N47K	rs199474808	D;P	0,00057
MYL2	12_111356937_C_T	ENSG00000111245:ENST00000550439:exon1:c.G7A:p.E3K,ENSG00000111245:ENST00000228841:exon2:c.G64A:p.E22K,ENSG00000111245:ENST00000548438:exon2:c.G64A:p.E22K	rs104894368	D;P	0,00057
MYL2	12_111356964_C_T	ENSG00000111245:ENST00000228841:exon2:c.G37A:p.A13T,ENSG00000111245:ENST00000548438:exon2:c.G37A:p.A13T	rs104894363	D;P	0,00115
MYL3	3_46899903_T_C	ENSG00000160808:ENST00000292327:exon5:c.A530G:p.E177G,ENSG00000160808:ENST00000395869:exon5:c.A530G:p.E177G	rs193922391	D;S	0,00058
MYL3	3_46900970_G_A	ENSG00000160808:ENST00000292327:exon4:c.C476T:p.T159M,ENSG00000160808:ENST00000395869:exon4:c.C476T:p.T159M		S	0,00058
MYL3	3_46900986_G_A	ENSG00000160808:ENST00000292327:exon4:c.C460T:p.R154C,ENSG00000160808:ENST00000395869:exon4:c.C460T:p.R154C	rs143852164	D;S	0,00115
MYL3	3_46901009_C_T	ENSG00000160808:ENST00000292327:exon4:c.G437A:p.G146D,ENSG00000160808:ENST00000395869:exon4:c.G437A:p.G146D		S	0,00058
MYL3	3_46902285_C_T	ENSG00000160808:ENST00000292327:exon3:c.G188A:p.R63H,ENSG00000160808:ENST00000395869:exon3:c.G188A:p.R63H	rs139354105	D;S	0,00058
PDLIM3	4_186423516_T_C	ENSG00000154553:ENST00000284771:exon7:c.A883G:p.T295A,ENSG00000154553:ENST00000284770:exon8:c.A1027G:p.T343A		N	0,00172

PDLIM3	4_186423579_C_T	ENSG00000154553:ENST00000284771:exon7:c.G820A:p.D274N,ENSG00000154553:ENST00000284770:exon8:c.G964A:p.D322N		S	0,00057
PDLIM3	4_186427754_C_T	ENSG00000154553:ENST00000284771:exon5:c.G571A:p.D191N,ENSG00000154553:ENST00000284770:exon6:c.G715A:p.D239N	rs142143310	D	0,00058
PDLIM3	4_186427786_G_A	ENSG00000154553:ENST00000284771:exon5:c.C539T:p.P180L,ENSG00000154553:ENST00000284770:exon6:c.C683T:p.P228L	rs201185673	D	0,00115
PDLIM3	4_186429568_C_A	ENSG00000154553:ENST00000284770:exon5:c.G547T:p.V183L		N	0,00057
PDLIM3	4_186435447_A_G	ENSG00000154553:ENST00000284767:exon5:c.T563C:p.L188P		N	0,00057
PDLIM3	4_186435483_G_C	ENSG00000154553:ENST00000284770:exon4:c.C339G:p.N113K,ENSG00000154553:ENST00000284767:exon5:c.C527G:p.T176S		N	0,00057
PDLIM3	4_186444600_C_T	ENSG00000154553:ENST00000505886:exon2:c.G94A:p.G32R,ENSG00000154553:ENST00000512293:exon2:c.G94A:p.G32R	rs200354645	D	0,00115
PDLIM3	4_186446250_T_C	ENSG00000154553:ENST00000284767:exon2:c.A169G:p.T57A,ENSG00000154553:ENST00000284770:exon2:c.A169G:p.T57A,ENSG00000154553:ENST00000284771:exon2:c.A169G:p.T57A	rs142951316	D	0,00115
PKP2	12_32949101_G_T	ENSG00000057294:ENST00000340811:exon11:c.C2299A:p.R767S,ENSG00000057294:ENST00000070846:exon12:c.C2431A:p.R811S	rs139734328	D	0,00058
PKP2	12_32955491_C_G	ENST00000340811:exon11:c.2014-1G>C,ENST00000070846:exon12:c.2146-1G>C	rs193922674	D;P	0,00057
PKP2	12_32975431_A_C	ENSG00000057294:ENST00000340811:exon8:c.T1809G:p.C603W,ENSG00000057294:ENST00000070846:exon9:c.T1941G:p.C647W	rs149392678	D;S	0,00114
PKP2	12_32994007_C_-	ENSG00000057294:ENST00000340811:exon6:c.1511delG:p.G504fs,ENSG00000057294:ENST00000070846:exon7:c.1643delG:p.G548fs		P	0,00057
PKP2	12_32994100_T_C	ENSG00000057294:ENST00000340811:exon6:c.A1418G:p.N473S,ENSG00000057294:ENST00000070846:exon7:c.A1550G:p.N517S	rs144536197	D	0,00115
PKP2	12_32996206_C_T	ENSG00000057294:ENST00000070846:exon6:c.G1420A:p.A474T	rs138538072	D	0,00193
PKP2	12_33021936_C_T	ENSG00000057294:ENST00000070846:exon4:c.G1095A:p.M365I,ENSG00000057294:ENST00000340811:exon4:c.G1095A:p.M365I		N	0,00058
PKP2	12_33021997_C_G	ENST00000340811:exon5:c.1035-1G>C,ENST00000070846:exon5:c.1035-1G>C		L	0,00057
PKP2	12_33030850_C_A	ENSG00000057294:ENST00000070846:exon3:c.G964T:p.G322C,ENSG00000057294:ENST00000340811:exon3:c.G964T:p.G322C		S	0,00064
PKP2	12_33031039_C_A	ENSG00000057294:ENST00000070846:exon3:c.G775T:p.E259X,ENSG00000057294:ENST00000340811:exon3:c.G775T:p.E259X		P	0,00062
PKP2	12_33031321_C_T	ENSG00000057294:ENST00000070846:exon3:c.G493A:p.D165N,ENSG00000057294:ENST00000340811:exon3:c.G493A:p.D165N		S	0,00118

PKP2	12_33031329_G_A	ENSG00000057294:ENST00000070846:exon3:c.C485T:p.T162M,ENSG00000057294:ENST00000340811:exon3:c.C485T:p.T162M		N	0,00059
PKP2	12_33031468_T_C	ENSG00000057294:ENST00000070846:exon3:c.A346G:p.T116A,ENSG00000057294:ENST00000340811:exon3:c.A346G:p.T116A		N	0,00057
PKP2	12_33031883_G_C	ENSG00000057294:ENST00000070846:exon2:c.C307G:p.P103A,ENSG00000057294:ENST00000340811:exon2:c.C307G:p.P103A		N	0,00057
PKP2	12_33031883_G_T	ENSG00000057294:ENST00000070846:exon2:c.C307A:p.P103T,ENSG00000057294:ENST00000340811:exon2:c.C307A:p.P103T	rs139215336	D	0,00057
PKP2	12_33031888_C_T	ENSG00000057294:ENST00000070846:exon2:c.G302A:p.R101H,ENSG00000057294:ENST00000340811:exon2:c.G302A:p.R101H	rs149542398	D;P	0,00115
PKP2	12_33031931_C_G	ENSG00000057294:ENST00000070846:exon2:c.G259C:p.V87L,ENSG00000057294:ENST00000340811:exon2:c.G259C:p.V87L		P	0,00057
PKP2	12_33049492_C_A	ENSG00000057294:ENST00000070846:exon1:c.G174T:p.E58D,ENSG00000057294:ENST00000340811:exon1:c.G174T:p.E58D	rs146708884	D;P	0,00771
PKP4	2_159313728_A_C			L	0,00993
PKP4	2_159459593_A_G	ENSG00000144283:ENST00000389757:exon4:c.A257G:p.K86R,ENSG00000144283:ENST00000389759:exon4:c.A257G:p.K86R,ENSG00000144283:ENST00000421462:exon4:c.A257G:p.K86R,ENSG00000144283:ENST00000426248:exon4:c.A257G:p.K86R	rs144452632	D	0,00057
PKP4	2_159459601_C_T	ENSG00000144283:ENST00000389757:exon4:c.C265T:p.P89S,ENSG00000144283:ENST00000389759:exon4:c.C265T:p.P89S,ENSG00000144283:ENST00000421462:exon4:c.C265T:p.P89S,ENSG00000144283:ENST00000426248:exon4:c.C265T:p.P89S	rs141436976	D	0,00057
PKP4	2_159477550_A_G	ENSG00000144283:ENST00000389757:exon5:c.A329G:p.Q110R,ENSG00000144283:ENST00000389759:exon5:c.A329G:p.Q110R,ENSG00000144283:ENST00000421462:exon5:c.A329G:p.Q110R,ENSG00000144283:ENST00000426248:exon5:c.A329G:p.Q110R	rs148019751	D	0,00057
PKP4	2_159477598_A_G	ENSG00000144283:ENST00000389757:exon5:c.A377G:p.Y126C,ENSG00000144283:ENST00000389759:exon5:c.A377G:p.Y126C,ENSG00000144283:ENST00000421462:exon5:c.A377G:p.Y126C,ENSG00000144283:ENST00000426248:exon5:c.A377G:p.Y126C		S	0,00114
PKP4	2_159481523_G_C	ENSG00000144283:ENST00000389757:exon7:c.G737C:p.G246A,ENSG00000144283:ENST00000389759:exon7:c.G737C:p.G246A		N	0,00058
PKP4	2_159481526_C_T	ENSG00000144283:ENST00000389757:exon7:c.C740T:p.S247F,ENSG00000144283:ENST00000389759:exon7:c.C740T:p.S247F		N	0,00058
PKP4	2_159481840_C_A	ENSG00000144283:ENST00000389757:exon7:c.C1054A:p.P352T,ENSG00000144283:ENST00000389759:exon7:c.C1054A:p.P352T		N	0,00058
PKP4	2_159481864_G_T	ENSG00000144283:ENST00000389757:exon7:c.G1078T:p.D360Y,ENSG00000144283:ENST00000389759:exon7:c.G1078T:p.D360Y	rs147567711	D;S	0,00058

PKP4	2_159481865_A_G	ENSG00000144283:ENST00000389757:exon7:c.A1079G:p.D360G,ENSG00000144283:ENST00000389759:exon7:c.A1079G:p.D360G	rs147809285	D	0,00058
PKP4	2_159497153_G_A	ENSG00000144283:ENST00000389757:exon10:c.G1577A:p.R526H,ENSG00000144283:ENST00000389759:exon10:c.G1577A:p.R526H	rs144253065	D;S	0,00057
PKP4	2_159499094_G_A	ENSG00000144283:ENST00000389757:exon11:c.G1792A:p.V598I,ENSG00000144283:ENST00000389759:exon11:c.G1792A:p.V598I		N	0,00057
PKP4	2_159499181_A_G	ENSG00000144283:ENST00000389757:exon11:c.A1879G:p.I627V,ENSG00000144283:ENST00000389759:exon11:c.A1879G:p.I627V		N	0,00057
PKP4	2_159517919_A_G	ENSG00000144283:ENST00000389757:exon13:c.A2168G:p.Y723C,ENSG00000144283:ENST00000389759:exon13:c.A2168G:p.Y723C	rs150425961	D;S	0,00057
PKP4	2_159519410_C_T	ENSG00000144283:ENST00000389757:exon14:c.C2213T:p.T738M,ENSG00000144283:ENST00000389759:exon14:c.C2213T:p.T738M	rs201257617	D;S	0,00058
PKP4	2_159519557_G_A	ENSG00000144283:ENST00000389757:exon14:c.G2360A:p.G787E,ENSG00000144283:ENST00000389759:exon14:c.G2360A:p.G787E		S	0,00057
PKP4	2_159519581_G_A	ENSG00000144283:ENST00000389757:exon14:c.G2384A:p.R795K,ENSG00000144283:ENST00000389759:exon14:c.G2384A:p.R795K	rs139221917	D	0,00115
PKP4	2_159519846_G_T	ENSG00000144283:ENST00000389757:exon15:c.G2466T:p.W822C,ENSG00000144283:ENST00000389759:exon15:c.G2466T:p.W822C		S	0,00058
PKP4	2_159523058_G_A	ENSG00000144283:ENST00000389757:exon16:c.G2711A:p.R904H,ENSG00000144283:ENST00000389759:exon16:c.G2711A:p.R904H		S	0,00057
PKP4	2_159530233_C_T	ENSG00000144283:ENST00000389757:exon18:c.C2969T:p.T990I,ENSG00000144283:ENST00000389759:exon18:c.C2969T:p.T990I	rs190318071	D	0,00057
PKP4	2_159530257_G_A	ENSG00000144283:ENST00000389757:exon18:c.G2993A:p.R998Q,ENSG00000144283:ENST00000389759:exon18:c.G2993A:p.R998Q		S	0,00057
PKP4	2_159536972_C_G	ENSG00000144283:ENST00000389757:exon21:c.C3233G:p.S1078C,ENSG00000144283:ENST00000389759:exon22:c.C3362G:p.S1121C		S	0,00114
PKP4	2_159537050_G_A	ENSG00000144283:ENST00000389757:exon21:c.G3311A:p.R1104Q,ENSG00000144283:ENST00000389759:exon22:c.G3440A:p.R1147Q		N	0,00057
PLN	6_118879986_A_T			L	0,00057
PLN	6_118880110_G_A	ENSG00000198523:ENST00000357525:exon2:c.G26A:p.R9H		S	0,00057
PLN	6_118880137_T_C	ENSG00000198523:ENST00000357525:exon2:c.T53C:p.I18T		N	0,00057
PLN	6_118880145_-_CT	ENSG00000198523:ENST00000357525:exon2:c.61_62insCT:p.P21fs		L	0,00057
PLN	6_118880157_C_T	ENSG00000198523:ENST00000357525:exon2:c.C73T:p.R25C		N	0,00057
PLN	6_118880158_G_A	ENSG00000198523:ENST00000357525:exon2:c.G74A:p.R25H		N	0,00057

PLN	6_118880236_T_C	ENSG00000198523:ENST00000357525:exon2:c.T152C:p.L51P		S	0,00057
PNN	14_39646638_A_G	ENSG00000100941:ENST00000216832:exon4:c.A277G:p.R93G,ENSG00000100941:ENST00000553331:exon4:c.A277G:p.R93G,ENSG00000100941:ENST00000556530:exon4:c.A277G:p.R93G		N	0,00058
PNN	14_39649827_C_T	ENSG00000100941:ENST00000216832:exon9:c.C914T:p.A305V	rs140529795	D	0,00057
PNN	14_39650139_A_G	ENSG00000100941:ENST00000216832:exon9:c.A1226G:p.N409S	rs143201887	D	0,00057
PNN	14_39650169_A_G	ENSG00000100941:ENST00000216832:exon9:c.A1256G:p.N419S	rs145173115	D	0,00057
PNN	14_39650261_A_G	ENSG00000100941:ENST00000216832:exon9:c.A1348G:p.S450G	rs145827544	D	0,00057
PNN	14_39650320_A_T	ENSG00000100941:ENST00000216832:exon9:c.A1407T:p.Q469H	rs148193612	D	0,00058
PNN	14_39650334_CTCAAC_-	ENSG00000100941:ENST00000216832:exon9:c.1421_1426del:p.474_476del		N	0,00058
PNN	14_39650338_ACCTCA_-	ENSG00000100941:ENST00000216832:exon9:c.1425_1430del:p.475_477del		N	0,00058
PNN	14_39650559_A_C	ENSG00000100941:ENST00000216832:exon9:c.A1646C:p.H549P		N	0,00057
PNN	14_39650883_C_G	ENSG00000100941:ENST00000216832:exon9:c.C1970G:p.T657S		N	0,00057
RBM20	10_112404334_-_GCA	ENSG00000203867:ENST00000369519:exon1:c.122_123insGCA:p.M41delinsMQ		N	0,01980
RBM20	10_112404342_-_AGC	ENSG00000203867:ENST00000369519:exon1:c.130_131insAGC:p.P44delinsQP		N	0,01754
RBM20	10_112541047_G_T	ENSG00000203867:ENST00000369519:exon2:c.G680T:p.G227V	rs202238753	D	0,00058
RBM20	10_112541290_T_C	ENSG00000203867:ENST00000369519:exon2:c.T923C:p.V308A		N	0,00059
RBM20	10_112541394_C_T	ENSG00000203867:ENST00000369519:exon2:c.C1027T:p.H343Y	rs112226602	D	0,00058
RBM20	10_112541424_G_A	ENSG00000203867:ENST00000369519:exon2:c.G1057A:p.E353K		S	0,00058
RBM20	10_112541460_G_A	ENSG00000203867:ENST00000369519:exon2:c.G1093A:p.G365R	rs201047984	D;S	0,00058
RBM20	10_112541502_G_A	ENSG00000203867:ENST00000369519:exon2:c.G1135A:p.G379R	rs199842148	D	0,00057
RBM20	10_112541511_G_T	ENSG00000203867:ENST00000369519:exon2:c.G1144T:p.A382S		N	0,00057
RBM20	10_112541568_G_A	ENSG00000203867:ENST00000369519:exon2:c.G1201A:p.D401N		S	0,00057
RBM20	10_112541632_T_C	ENSG00000203867:ENST00000369519:exon2:c.T1265C:p.F422S		S	0,00057
RBM20	10_112544571_C_T	ENSG00000203867:ENST00000369519:exon5:c.C1451T:p.T484I	rs116442272	D	0,00057
RBM20	10_112557371_G_A	ENSG00000203867:ENST00000369519:exon6:c.G1633A:p.V545I		N	0,00057
RBM20	10_112559642_G_A	ENSG00000203867:ENST00000369519:exon7:c.G1766A:p.R589Q		N	0,00115
RBM20	10_112572113_C_T	ENSG00000203867:ENST00000369519:exon9:c.C1958T:p.T653I		S	0,00060

RBM20	10_112572169_G_A	ENSG00000203867:ENST00000369519:exon9:c.G2014A:p.G672S		N	0,00061
RBM20	10_112572271_C_A	ENSG00000203867:ENST00000369519:exon9:c.C2116A:p.P706T		N	0,00059
RBM20	10_112572356_G_A	ENSG00000203867:ENST00000369519:exon9:c.G2201A:p.R734Q		S	0,00059
RBM20	10_112572368_C_T	ENSG00000203867:ENST00000369519:exon9:c.C2213T:p.P738L		N	0,00059
RBM20	10_112572394_C_T	ENSG00000203867:ENST00000369519:exon9:c.C2239T:p.H747Y		S	0,00059
RBM20	10_112572419_G_A	ENSG00000203867:ENST00000369519:exon9:c.G2264A:p.R755H		S	0,00058
RBM20	10_112572473_A_G	ENSG00000203867:ENST00000369519:exon9:c.A2318G:p.K773R	rs181769913	D	0,00058
RBM20	10_112572488_C_T	ENSG00000203867:ENST00000369519:exon9:c.C2333T:p.A778V		N	0,00058
RBM20	10_112581039_G_A	ENSG00000203867:ENST00000369519:exon11:c.G2662A:p.D888N	rs201370621	D;P	0,00344
RBM20	10_112581264_A_G	ENSG00000203867:ENST00000369519:exon11:c.A2887G:p.K963E		N	0,00057
RBM20	10_112581282_G_A	ENSG00000203867:ENST00000369519:exon11:c.G2905A:p.V969I		N	0,00057
RBM20	10_112581381_C_G	ENSG00000203867:ENST00000369519:exon11:c.C3004G:p.L1002V		N	0,00115
RBM20	10_112581391_A_G	ENSG00000203867:ENST00000369519:exon11:c.A3014G:p.D1005G		S	0,00057
RBM20	10_112581400_G_A	ENSG00000203867:ENST00000369519:exon11:c.G3023A:p.R1008Q		N	0,00115
RBM20	10_112581424_G_C	ENSG00000203867:ENST00000369519:exon11:c.G3047C:p.G1016A		S	0,00057
RBM20	10_112581492_C_T	ENSG00000203867:ENST00000369519:exon11:c.C3115T:p.P1039S		N	0,00057
RBM20	10_112581642_C_G	ENSG00000203867:ENST00000369519:exon11:c.C3265G:p.P1089A	rs147356378	D	0,00058
RBM20	10_112581643_C_G	ENSG00000203867:ENST00000369519:exon11:c.C3266G:p.P1089R		N	0,00057
RYR2	1_237538086_G_A	ENSG00000198626:ENST00000542537:exon6:c.G406A:p.D136N,ENSG00000198626:ENST00000366574:exon7:c.G454A:p.D152N,ENSG00000198626:ENST00000360064:exon8:c.G448A:p.D150N		N	0,00058
RYR2	1_237608773_A_G	ENSG00000198626:ENST00000542537:exon13:c.A1195G:p.T399A,ENSG00000198626:ENST00000366574:exon14:c.A1243G:p.T415A,ENSG00000198626:ENST00000360064:exon15:c.A1237G:p.T413A		N	0,00057
RYR2	1_237619933_C_G	ENSG00000198626:ENST00000542537:exon15:c.C1462G:p.R488G,ENSG00000198626:ENST00000366574:exon16:c.C1510G:p.R504G,ENSG00000198626:ENST00000360064:exon17:c.C1504G:p.R502G		S	0,00057
RYR2	1_237619982_G_T	ENSG00000198626:ENST00000542537:exon15:c.G1511T:p.R504L,ENSG00000198626:ENST00000366574:exon16:c.G1559T:p.R520L,ENSG00000198626:ENST00000360064:exon17:c.G1553T:p.R518L		N	0,00057
RYR2	1_237659825_T_G	ENSG00000198626:ENST00000542537:exon19:c.T1928G:p.I643S,ENSG00000198626:ENST00000366574:exon20:c.T1976G:p.I659S,ENSG00000198626:ENST00000360064:exon21:c.T1970G:p.I657S		S	0,00057

RYR2	1_237659929_C_T	ENSG00000198626:ENST00000542537:exon19:c.C2032T:p.R678X,ENSG00000198626:ENST00000366574:exon20:c.C2080T:p.R694X,ENSG00000198626:ENST00000360064:exon21:c.C2074T:p.R692X		L	0,00057
RYR2	1_237659953_G_A	ENSG00000198626:ENST00000542537:exon19:c.G2056A:p.G686R,ENSG00000198626:ENST00000366574:exon20:c.G2104A:p.G702R,ENSG00000198626:ENST00000360064:exon21:c.G2098A:p.G700R		S	0,00057
RYR2	1_237660057_G_A	ENST00000366574:exon20:c.2203+5G>A,ENST00000360064:exon21:c.2197+5G>A,ENST00000542537:exon19:c.2155+5G>A		L	0,00057
RYR2	1_237664074_G_A	ENSG00000198626:ENST00000542537:exon20:c.G2219A:p.S740N,ENSG00000198626:ENST00000366574:exon21:c.G2267A:p.S756N,ENSG00000198626:ENST00000360064:exon22:c.G2261A:p.S754N	rs193922623	D;S	0,00057
RYR2	1_237664112_C_G	ENSG00000198626:ENST00000542537:exon20:c.C2257G:p.R753G,ENSG00000198626:ENST00000366574:exon21:c.C2305G:p.R769G,ENSG00000198626:ENST00000360064:exon22:c.C2299G:p.R767G		S	0,00057
RYR2	1_237664149_A_G	ENSG00000198626:ENST00000542537:exon20:c.A2294G:p.N765S,ENSG00000198626:ENST00000366574:exon21:c.A2342G:p.N781S,ENSG00000198626:ENST00000360064:exon22:c.A2336G:p.N779S		S	0,00057
RYR2	1_237666593_C_T	ENSG00000198626:ENST00000542537:exon21:c.C2353T:p.R785C,ENSG00000198626:ENST00000366574:exon22:c.C2401T:p.R801C,ENSG00000198626:ENST00000360064:exon23:c.C2395T:p.R799C		S	0,00057
RYR2	1_237666731_A_G	ENSG00000198626:ENST00000542537:exon21:c.A2491G:p.T831A,ENSG00000198626:ENST00000366574:exon22:c.A2539G:p.T847A,ENSG00000198626:ENST00000360064:exon23:c.A2533G:p.T845A		N	0,00057
RYR2	1_237670112_C_G	ENSG00000198626:ENST00000542537:exon22:c.C2668G:p.P890A,ENSG00000198626:ENST00000366574:exon23:c.C2716G:p.P906A,ENSG00000198626:ENST00000360064:exon24:c.C2710G:p.P904A		S	0,00057
RYR2	1_237693732_T_C	ENSG00000198626:ENST00000542537:exon24:c.T2780C:p.L927S,ENSG00000198626:ENST00000366574:exon25:c.T2828C:p.L943S,ENSG00000198626:ENST00000360064:exon26:c.T2822C:p.L941S		S	0,00058
RYR2	1_237711759_G_T	ENSG00000198626:ENST00000542537:exon25:c.G2887T:p.A963S,ENSG00000198626:ENST00000366574:exon26:c.G2935T:p.A979S,ENSG00000198626:ENST00000360064:exon27:c.G2929T:p.A977S	rs202015519	D	0,00057
RYR2	1_237711861_C_T	ENSG00000198626:ENST00000542537:exon25:c.C2989T:p.R997W,ENSG00000198626:ENST00000366574:exon26:c.C3037T:p.R1013W,ENSG00000198626:ENST00000360064:exon27:c.C3031T:p.R1011W		N	0,00057
RYR2	1_237711862_G_A	ENSG00000198626:ENST00000542537:exon25:c.G2990A:p.R997Q,ENSG00000198626:ENST00000366574:exon26:c.G3038A:p.R1013Q,ENSG00000198626:ENST00000360064:exon27:c.G3032A:p.R1011Q	rs149514924	D;P	0,00115
RYR2	1_237713928_C_T	ENSG00000198626:ENST00000542537:exon26:c.C3103T:p.R1035C,ENSG00000198626:ENST00000366574:exon27:c.C3151T:p.R1051C,ENSG00000198626:ENST00000360064:exon28:c.C3145T:p.R1049C		N	0,00057

RYR2	1_237713940_C_T	ENSG00000198626:ENST00000542537:exon26:c.C3115T:p.R1039C,ENSG00000198626:ENST00000366574:exon27:c.C3163T:p.R1055C,ENSG00000198626:ENST00000360064:exon28:c.C3157T:p.R1053C		S	0,00057
RYR2	1_237730008_G_A	ENSG00000198626:ENST00000542537:exon27:c.G3308A:p.R1103H,ENSG00000198626:ENST00000366574:exon28:c.G3356A:p.R1119H,ENSG00000198626:ENST00000360064:exon29:c.G3350A:p.R1117H	rs201312753	D;S	0,00116
RYR2	1_237732502_G_C	ENSG00000198626:ENST00000542537:exon28:c.G3433C:p.V1145L,ENSG00000198626:ENST00000366574:exon29:c.G3481C:p.V1161L,ENSG00000198626:ENST00000360064:exon30:c.G3475C:p.V1159L		S	0,00057
RYR2	1_237754201_G_C	ENSG00000198626:ENST00000542537:exon30:c.G4021C:p.D1341H,ENSG00000198626:ENST00000366574:exon31:c.G4069C:p.D1357H,ENSG00000198626:ENST00000360064:exon32:c.G4063C:p.D1355H	rs193922626	D	0,00114
RYR2	1_237758826_T_C	ENSG00000198626:ENST00000542537:exon33:c.T4417C:p.C1473R,ENSG00000198626:ENST00000366574:exon34:c.T4465C:p.C1489R,ENSG00000198626:ENST00000360064:exon35:c.T4459C:p.C1487R	rs200450676	D;S	0,00058
RYR2	1_237777530_G_C	ENSG00000198626:ENST00000542537:exon36:c.G5054C:p.G1685A,ENSG00000198626:ENST00000366574:exon37:c.G5102C:p.G1701A,ENSG00000198626:ENST00000360064:exon38:c.G5096C:p.G1699A		S	0,00057
RYR2	1_237777694_T_G	ENSG00000198626:ENST00000542537:exon36:c.T5218G:p.S1740A,ENSG00000198626:ENST00000366574:exon37:c.T5266G:p.S1756A,ENSG00000198626:ENST00000360064:exon38:c.T5260G:p.S1754A		S	0,00057
RYR2	1_237777722_C_G	ENSG00000198626:ENST00000542537:exon36:c.C5246G:p.S1749C,ENSG00000198626:ENST00000366574:exon37:c.C5294G:p.S1765C,ENSG00000198626:ENST00000360064:exon38:c.C5288G:p.S1763C		S	0,00057
RYR2	1_237778090_C_T	ENSG00000198626:ENST00000542537:exon36:c.C5614T:p.R1872W,ENSG00000198626:ENST00000366574:exon37:c.C5662T:p.R1888W,ENSG00000198626:ENST00000360064:exon38:c.C5656T:p.R1886W		N	0,00058
RYR2	1_237778108_C_A	ENSG00000198626:ENST00000542537:exon36:c.C5632A:p.L1878I,ENSG00000198626:ENST00000366574:exon37:c.C5680A:p.L1894I,ENSG00000198626:ENST00000360064:exon38:c.C5674A:p.L1892I		S	0,00057
RYR2	1_237787064_G_C	ENST00000366574:exon39:c.5917-1G>C,ENST00000360064:exon40:c.5911-1G>C,ENST00000542537:exon38:c.5869-1G>C		L	0,00057
RYR2	1_237794736_G_A	ENSG00000198626:ENST00000542537:exon41:c.G6402A:p.M2134I,ENSG00000198626:ENST00000366574:exon42:c.G6450A:p.M2150I,ENSG00000198626:ENST00000360064:exon43:c.G6444A:p.M2148I		N	0,00057

RYR2	1_237813288_G_A	ENSG00000198626:ENST00000542537:exon49:c.G7576A:p.A2526T,ENSG00000198626:ENST00000366574:exon50:c.G7624A:p.A2542T,ENSG00000198626:ENST00000360064:exon51:c.G7618A:p.A2540T		N	0,00057
RYR2	1_237814727_A_G	ENSG00000198626:ENST00000542537:exon50:c.A7702G:p.M2568V,ENSG00000198626:ENST00000366574:exon51:c.A7750G:p.M2584V,ENSG00000198626:ENST00000360064:exon52:c.A7744G:p.M2582V		N	0,00057
RYR2	1_237821276_T_C	ENSG00000198626:ENST00000542537:exon53:c.T8114C:p.I2705T,ENSG00000198626:ENST00000366574:exon54:c.T8162C:p.I2721T,ENSG00000198626:ENST00000360064:exon55:c.T8156C:p.I2719T	rs201500134	D	0,00230
RYR2	1_237823350_G_T	ENSG00000198626:ENST00000542537:exon54:c.G8226T:p.K2742N,ENSG00000198626:ENST00000366574:exon55:c.G8274T:p.K2758N,ENSG00000198626:ENST00000360064:exon56:c.G8268T:p.K2756N		S	0,00057
RYR2	1_237868518_G_T	ENSG00000198626:ENST00000540213:exon5:c.G440T:p.R147L,ENSG00000198626:ENST00000542288:exon6:c.G320T:p.R107L,ENSG00000198626:ENST00000542537:exon66:c.G9407T:p.R3136L,ENSG00000198626:ENST00000366574:exon67:c.G9455T:p.R3152L,ENSG00000198626:ENST00000360064:exon68:c.G9449T:p.R3150L		N	0,00057
RYR2	1_237868623_A_G	ENSG00000198626:ENST00000540213:exon5:c.A545G:p.K182R,ENSG00000198626:ENST00000542288:exon6:c.A425G:p.K142R,ENSG00000198626:ENST00000542537:exon66:c.A9512G:p.K3171R,ENSG00000198626:ENST00000366574:exon67:c.A9560G:p.K3187R,ENSG00000198626:ENST00000360064:exon68:c.A9554G:p.K3185R	rs184218219	D;S	0,00057
RYR2	1_237868632_G_A	ENSG00000198626:ENST00000540213:exon5:c.G554A:p.R185Q,ENSG00000198626:ENST00000542288:exon6:c.G434A:p.R145Q,ENSG00000198626:ENST00000542537:exon66:c.G9521A:p.R3174Q,ENSG00000198626:ENST00000366574:exon67:c.G9569A:p.R3190Q,ENSG00000198626:ENST00000360064:exon68:c.G9563A:p.R3188Q		S	0,00057
RYR2	1_237875041_T_C	ENST00000366574:exon71:c.10231-4T>C,ENST00000360064:exon72:c.10225-4T>C,ENST00000542537:exon70:c.10183-4T>C,ENST00000542288:exon10:c.1096-4T>C,ENST00000540213:exon9:c.1216-4T>C	rs117180147	D;L	0,00058
RYR2	1_237886554_C_G	ENSG00000198626:ENST00000542288:exon13:c.C1546G:p.L516V,ENSG00000198626:ENST00000542537:exon73:c.C10633G:p.L3545V,ENSG00000198626:ENST00000366574:exon74:c.C10681G:p.L3561V,ENSG00000198626:ENST00000360064:exon75:c.C10675G:p.L3559V		N	0,00057
RYR2	1_237947785_T_A	ENSG00000198626:ENST00000542288:exon30:c.T3695A:p.M1232K,ENSG00000198626:ENST00000542537:exon89:c.T12725A:p.M4242K,ENSG00000198626:ENST00000366574:exon90:c.T12773A:p.M4258K,ENSG00000198626:ENST00000360064:exon92:c.T12791A:p.M4264K		N	0,00058
RYR2	1_237948105_G_A	ENSG00000198626:ENST00000542288:exon30:c.G4015A:p.D1339N,ENSG00000198626:ENST00000542537:exon89:c.G13045A:p.D4349N,ENSG00000198626:ENST00000366574:exon90:c.G13093A:p.D4365N,ENSG00000198626:ENST00000360064:exon92:c.G13111A:p.D4371N		N	0,00058

RYR2	1_237948273_-_TAAT	ENST00000366574:exon90:c.13260+1->TAAT,ENST00000360064:exon92:c.13278+1->TAAT,ENST00000542537:exon89:c.13212+1->TAAT,ENST00000542288:exon30:c.4182+1->TAAT		L	0,00057
RYR2	1_237961442_C_G	ENSG00000198626:ENST00000536033:exon5:c.C361G:p.P121A,ENSG00000198626:ENST00000542537:exon96:c.C14014G:p.P4672A,ENSG00000198626:ENST00000366574:exon97:c.C14062G:p.P4688A,ENSG00000198626:ENST00000360064:exon99:c.C14080G:p.P4694A		N	0,00057
RYR2	1_237969434_T_C	ENST00000366574:exon99:c.14152-3T>C,ENST00000360064:exon101:c.14170-3T>C,ENST00000542537:exon98:c.14104-3T>C,ENST00000536033:exon7:c.451-3T>C		L	0,00058
SCN5A	3_38591847_G_C	ENSG00000183873:ENST00000414099:exon26:c.C5962G:p.P1988A,ENSG00000183873:ENST00000449557:exon26:c.C5854G:p.P1952A,ENSG00000183873:ENST00000450102:exon26:c.C5854G:p.P1952A,ENSG00000183873:ENST00000423572:exon27:c.C6013G:p.P2005A,ENSG00000183873:ENST00000425664:exon27:c.C5962G:p.P1988A,ENSG00000183873:ENST00000451551:exon27:c.C5854G:p.P1952A,ENSG00000183873:ENST00000455624:exon27:c.C5917G:p.P1973A,ENSG00000183873:ENST00000333535:exon28:c.C6016G:p.P2006A,ENSG00000183873:ENST00000413689:exon28:c.C6016G:p.P2006A,ENSG00000183873:ENST00000443581:exon28:c.C6013G:p.P2005A	rs45489199	D	0,00060
SCN5A	3_38591895_C_G	ENSG00000183873:ENST00000414099:exon26:c.G5914C:p.V1972L,ENSG00000183873:ENST00000449557:exon26:c.G5806C:p.V1936L,ENSG00000183873:ENST00000450102:exon26:c.G5806C:p.V1936L,ENSG00000183873:ENST00000423572:exon27:c.G5965C:p.V1989L,ENSG00000183873:ENST00000425664:exon27:c.G5914C:p.V1972L,ENSG00000183873:ENST00000451551:exon27:c.G5806C:p.V1936L,ENSG00000183873:ENST00000455624:exon27:c.G5869C:p.V1957L,ENSG00000183873:ENST00000333535:exon28:c.G5968C:p.V1990L,ENSG00000183873:ENST00000413689:exon28:c.G5968C:p.V1990L,ENSG00000183873:ENST00000443581:exon28:c.G5965C:p.V1989L		N	0,00060
SCN5A	3_38591978_G_A	ENSG00000183873:ENST00000414099:exon26:c.C5831T:p.P1944L,ENSG00000183873:ENST00000449557:exon26:c.C5723T:p.P1908L,ENSG00000183873:ENST00000450102:exon26:c.C5723T:p.P1908L,ENSG00000183873:ENST00000423572:exon27:c.C5882T:p.P1961L,ENSG00000183873:ENST00000425664:exon27:c.C5831T:p.P1944L,ENSG00000183873:ENST00000451551:exon27:c.C5723T:p.P1908L,ENSG00000183873:ENST00000455624:exon27:c.C5786T:p.P1929L,ENSG00000183873:ENST00000333535:exon28:c.C5885T:p.P1962L,ENSG00000183873:ENST00000413689:exon28:c.C5885T:p.P1962L,ENSG00000183873:ENST00000443581:exon28:c.C5882T:p.P1961L	rs199473638	D;P	0,00061
SCN5A	3_38592120_G_A	ENSG00000183873:ENST00000414099:exon26:c.C5689T:p.H1897Y,ENSG00000183873:ENST00000449557:exon26:c.C5581T:p.H1861Y,ENSG00000183873:ENST00000450102:exon26:c.C5581T:p.H1861Y,ENSG00000183873:ENST00000423572:exon27:c.C5740T:p.H1914Y,ENSG00000183873:ENST00000425664:exon27:c.C5689T:p.H1897Y,ENSG00000183873:ENST00000451551:exon27:c.C5581T:p.H1861Y,ENSG00000183873:ENST00000455624:exon27:c.C5644T:p.H1882Y,ENSG00000183873:ENST00000333535:exon28:c.C5743T:p.H1915Y,ENSG00000183873:ENST00000413689:exon28:c.C5743T:p.H1915Y,ENSG00000183873:ENST00000443581:exon28:c.C5740T:p.H1914Y		N	0,00058

SCN5A	3_38592408_C_T	ENSG00000183873:ENST00000414099:exon26:c.G5401A:p.D1801N,ENSG00000183873:ENST00000449557:exon26:c.G5293A:p.D1765N,ENSG00000183873:ENST00000450102:exon26:c.G5293A:p.D1765N,ENSG00000183873:ENST00000423572:exon27:c.G5452A:p.D1818N,ENSG00000183873:ENST00000425664:exon27:c.G5401A:p.D1801N,ENSG00000183873:ENST00000451551:exon27:c.G5293A:p.D1765N,ENSG00000183873:ENST00000455624:exon27:c.G5356A:p.D1786N,ENSG00000183873:ENST00000333535:exon28:c.G5455A:p.D1819N,ENSG00000183873:ENST00000413689:exon28:c.G5455A:p.D1819N,ENSG00000183873:ENST00000443581:exon28:c.G5452A:p.D1818N	rs137854619	D;P	0,00058
SCN5A	3_38592503_C_T	ENSG00000183873:ENST00000414099:exon26:c.G5306A:p.S1769N,ENSG00000183873:ENST00000449557:exon26:c.G5198A:p.S1733N,ENSG00000183873:ENST00000450102:exon26:c.G5198A:p.S1733N,ENSG00000183873:ENST00000423572:exon27:c.G5357A:p.S1786N,ENSG00000183873:ENST00000425664:exon27:c.G5306A:p.S1769N,ENSG00000183873:ENST00000451551:exon27:c.G5198A:p.S1733N,ENSG00000183873:ENST00000455624:exon27:c.G5261A:p.S1754N,ENSG00000183873:ENST00000333535:exon28:c.G5360A:p.S1787N,ENSG00000183873:ENST00000413689:exon28:c.G5360A:p.S1787N,ENSG00000183873:ENST00000443581:exon28:c.G5357A:p.S1786N	rs199473316	D;P	0,00172
SCN5A	3_38592527_G_A	ENSG00000183873:ENST00000414099:exon26:c.C5282T:p.T1761M,ENSG00000183873:ENST00000449557:exon26:c.C5174T:p.T1725M,ENSG00000183873:ENST00000450102:exon26:c.C5174T:p.T1725M,ENSG00000183873:ENST00000423572:exon27:c.C5333T:p.T1778M,ENSG00000183873:ENST00000425664:exon27:c.C5282T:p.T1761M,ENSG00000183873:ENST00000451551:exon27:c.C5174T:p.T1725M,ENSG00000183873:ENST00000455624:exon27:c.C5237T:p.T1746M,ENSG00000183873:ENST00000333535:exon28:c.C5336T:p.T1779M,ENSG00000183873:ENST00000413689:exon28:c.C5336T:p.T1779M,ENSG00000183873:ENST00000443581:exon28:c.C5333T:p.T1778M	rs199473634	D;S	0,00058
SCN5A	3_38597928_G_A	ENST00000414099:exon24:c.4383+4C>T,ENST00000425664:exon25:c.4383+4C>T,ENST00000443581:exon26:c.4434+4C>T,ENST00000451551:exon25:c.4275+4C>T,ENST00000413689:exon26:c.4437+4C>T,ENST00000423572:exon25:c.4434+4C>T,ENST00000333535:exon26:c.4437+4C>T,ENST00000455624:exon25:c.4434+4C>T,ENST00000450102:exon24:c.4275+4C>T,ENST00000449557:exon24:c.4275+4C>T		L	0,00066
SCN5A	3_38598027_T_G	ENSG00000183873:ENST00000414099:exon23:c.A4288C:p.I1430L,ENSG00000183873:ENST00000449557:exon23:c.A4180C:p.I1394L,ENSG00000183873:ENST00000450102:exon23:c.A4180C:p.I1394L,ENSG00000183873:ENST00000423572:exon24:c.A4339C:p.I1447L,ENSG00000183873:ENST00000425664:exon24:c.A4288C:p.I1430L,ENSG00000183873:ENST00000451551:exon24:c.A4180C:p.I1394L,ENSG00000183873:ENST00000455624:exon24:c.A4339C:p.I1447L,ENSG00000183873:ENST00000333535:exon25:c.A4342C:p.I1448L,ENSG00000183873:ENST00000413689:exon25:c.A4342C:p.I1448L,ENSG00000183873:ENST00000443581:exon25:c.A4339C:p.I1447L	rs199473250	D	0,00059

SCN5A	3_38603947_G_A	ENSG00000183873:ENST00000449557:exon20:c.C3760T:p.L1254F,ENSG00000183873:ENST00000450102:exon20:c.C3760T:p.L1254F,ENSG00000183873:ENST00000414099:exon21:c.C3922T:p.L1308F,ENSG00000183873:ENST00000423572:exon21:c.C3919T:p.L1307F,ENSG00000183873:ENST00000451551:exon21:c.C3760T:p.L1254F,ENSG00000183873:ENST00000455624:exon21:c.C3919T:p.L1307F,ENSG00000183873:ENST00000333535:exon22:c.C3922T:p.L1308F,ENSG00000183873:ENST00000413689:exon22:c.C3922T:p.L1308F,ENSG00000183873:ENST00000425664:exon22:c.C3922T:p.L1308F,ENSG00000183873:ENST00000443581:exon22:c.C3919T:p.L1307F	rs41313031	D;P	0,00079
SCN5A	3_38603958_G_A	ENSG00000183873:ENST00000449557:exon20:c.C3749T:p.T1250M,ENSG00000183873:ENST00000450102:exon20:c.C3749T:p.T1250M,ENSG00000183873:ENST00000414099:exon21:c.C3911T:p.T1304M,ENSG00000183873:ENST00000423572:exon21:c.C3908T:p.T1303M,ENSG00000183873:ENST00000451551:exon21:c.C3749T:p.T1250M,ENSG00000183873:ENST00000455624:exon21:c.C3908T:p.T1303M,ENSG00000183873:ENST00000333535:exon22:c.C3911T:p.T1304M,ENSG00000183873:ENST00000413689:exon22:c.C3911T:p.T1304M,ENSG00000183873:ENST00000425664:exon22:c.C3911T:p.T1304M,ENSG00000183873:ENST00000443581:exon22:c.C3908T:p.T1303M	rs199473603	D;P	0,00153
SCN5A	3_38603991_A_G	ENSG00000183873:ENST00000449557:exon20:c.T3716C:p.F1239S,ENSG00000183873:ENST00000450102:exon20:c.T3716C:p.F1239S,ENSG00000183873:ENST00000414099:exon21:c.T3878C:p.F1293S,ENSG00000183873:ENST00000423572:exon21:c.T3875C:p.F1292S,ENSG00000183873:ENST00000451551:exon21:c.T3716C:p.F1239S,ENSG00000183873:ENST00000455624:exon21:c.T3875C:p.F1292S,ENSG00000183873:ENST00000333535:exon22:c.T3878C:p.F1293S,ENSG00000183873:ENST00000413689:exon22:c.T3878C:p.F1293S,ENSG00000183873:ENST00000425664:exon22:c.T3878C:p.F1293S,ENSG00000183873:ENST00000443581:exon22:c.T3875C:p.F1292S	rs41311127	D;P	0,00076
SCN5A	3_38607905_C_T	ENSG00000183873:ENST00000449557:exon19:c.G3673A:p.V1225I,ENSG00000183873:ENST00000450102:exon19:c.G3673A:p.V1225I,ENSG00000183873:ENST00000414099:exon20:c.G3835A:p.V1279I,ENSG00000183873:ENST00000423572:exon20:c.G3832A:p.V1278I,ENSG00000183873:ENST00000451551:exon20:c.G3673A:p.V1225I,ENSG00000183873:ENST00000455624:exon20:c.G3832A:p.V1278I,ENSG00000183873:ENST00000333535:exon21:c.G3835A:p.V1279I,ENSG00000183873:ENST00000413689:exon21:c.G3835A:p.V1279I,ENSG00000183873:ENST00000425664:exon21:c.G3835A:p.V1279I,ENSG00000183873:ENST00000443581:exon21:c.G3832A:p.V1278I	rs199473341	D;P	0,00059
SCN5A	3_38608022_C_G	ENSG00000183873:ENST00000449557:exon19:c.G3556C:p.E1186Q,ENSG00000183873:ENST00000450102:exon19:c.G3556C:p.E1186Q,ENSG00000183873:ENST00000414099:exon20:c.G3718C:p.E1240Q,ENSG00000183873:ENST00000423572:exon20:c.G3715C:p.E1239Q,ENSG00000183873:ENST00000451551:exon20:c.G3556C:p.E1186Q,ENSG00000183873:ENST00000455624:exon20:c.G3715C:p.E1239Q,ENSG00000183873:ENST00000333535:exon21:c.G3718C:p.E1240Q,ENSG00000183873:ENST00000413689:exon21:c.G3718C:p.E1240Q,ENSG00000183873:ENST00000425664:exon21:c.G3718C:p.E1240Q,ENSG00000183873:ENST00000443581:exon21:c.G3715C:p.E1239Q	rs199473211	D;P	0,00058

SCN5A	3_38608058_A_G	ENSG00000183873:ENST00000449557:exon19:c.T3520C:p.Y1174H,ENSG00000183873:ENST00000450102:exon19:c.T3520C:p.Y1174H,ENSG00000183873:ENST00000414099:exon20:c.T3682C:p.Y1228H,ENSG00000183873:ENST00000423572:exon20:c.T3679C:p.Y1227H,ENSG00000183873:ENST00000451551:exon20:c.T3520C:p.Y1174H,ENSG00000183873:ENST00000455624:exon20:c.T3679C:p.Y1227H,ENSG00000183873:ENST00000333535:exon21:c.T3682C:p.Y1228H,ENSG00000183873:ENST00000413689:exon21:c.T3682C:p.Y1228H,ENSG00000183873:ENST00000425664:exon21:c.T3682C:p.Y1228H,ENSG00000183873:ENST00000443581:exon21:c.T3679C:p.Y1227H	rs199473205	D;P	0,00059
SCN5A	3_38622640_A_G	ENSG00000183873:ENST00000414099:exon16:c.T3010C:p.C1004R,ENSG00000183873:ENST00000423572:exon16:c.T3010C:p.C1004R,ENSG00000183873:ENST00000449557:exon16:c.T3010C:p.C1004R,ENSG00000183873:ENST00000450102:exon16:c.T3010C:p.C1004R,ENSG00000183873:ENST00000455624:exon16:c.T3010C:p.C1004R,ENSG00000183873:ENST00000333535:exon17:c.T3010C:p.C1004R,ENSG00000183873:ENST00000413689:exon17:c.T3010C:p.C1004R,ENSG00000183873:ENST00000425664:exon17:c.T3010C:p.C1004R,ENSG00000183873:ENST00000443581:exon17:c.T3010C:p.C1004R,ENSG00000183873:ENST00000451551:exon17:c.T3010C:p.C1004R	rs199473183	D;P	0,00157
SCN5A	3_38622706_A_G	ENSG00000183873:ENST00000414099:exon16:c.T2944C:p.C982R,ENSG00000183873:ENST00000423572:exon16:c.T2944C:p.C982R,ENSG00000183873:ENST00000449557:exon16:c.T2944C:p.C982R,ENSG00000183873:ENST00000450102:exon16:c.T2944C:p.C982R,ENSG00000183873:ENST00000455624:exon16:c.T2944C:p.C982R,ENSG00000183873:ENST00000333535:exon17:c.T2944C:p.C982R,ENSG00000183873:ENST00000413689:exon17:c.T2944C:p.C982R,ENSG00000183873:ENST00000425664:exon17:c.T2944C:p.C982R,ENSG00000183873:ENST00000443581:exon17:c.T2944C:p.C982R,ENSG00000183873:ENST00000451551:exon17:c.T2944C:p.C982R	rs199473182	D;P	0,00080
SCN5A	3_38627537_G_T	ENST00000414099:exon16:c.2437-5C>A,ENST00000425664:exon17:c.2437-5C>A,ENST00000443581:exon17:c.2437-5C>A,ENST00000451551:exon17:c.2437-5C>A,ENST00000413689:exon17:c.2437-5C>A,ENST00000423572:exon16:c.2437-5C>A,ENST00000333535:exon17:c.2437-5C>A,ENST00000455624:exon16:c.2437-5C>A,ENST00000450102:exon16:c.2437-5C>A,ENST00000449557:exon16:c.2437-5C>A	rs72549411	D;L	0,00174
SCN5A	3_38628928_C_A	ENSG00000183873:ENST00000414099:exon14:c.G2399T:p.R800L,ENSG00000183873:ENST00000423572:exon14:c.G2399T:p.R800L,ENSG00000183873:ENST00000449557:exon14:c.G2399T:p.R800L,ENSG00000183873:ENST00000450102:exon14:c.G2399T:p.R800L,ENSG00000183873:ENST00000455624:exon14:c.G2399T:p.R800L,ENSG00000183873:ENST00000333535:exon15:c.G2399T:p.R800L,ENSG00000183873:ENST00000413689:exon15:c.G2399T:p.R800L,ENSG00000183873:ENST00000425664:exon15:c.G2399T:p.R800L,ENSG00000183873:ENST00000443581:exon15:c.G2399T:p.R800L,ENSG00000183873:ENST00000451551:exon15:c.G2399T:p.R800L		N	0,00070

SCN5A	3_38639408_G_T	ENSG00000183873:ENST00000414099:exon13:c.C2074A:p.Q692K,ENSG00000183873:ENST00000423572:exon13:c.C2074A:p.Q692K,ENSG00000183873:ENST00000449557:exon13:c.C2074A:p.Q692K,ENSG00000183873:ENST00000450102:exon13:c.C2074A:p.Q692K,ENSG00000183873:ENST00000455624:exon13:c.C2074A:p.Q692K,ENSG00000183873:ENST00000333535:exon14:c.C2074A:p.Q692K,ENSG00000183873:ENST00000413689:exon14:c.C2074A:p.Q692K,ENSG00000183873:ENST00000425664:exon14:c.C2074A:p.Q692K,ENSG00000183873:ENST00000443581:exon14:c.C2074A:p.Q692K,ENSG00000183873:ENST00000451551:exon14:c.C2074A:p.Q692K	rs45553235	D;P	0,00124
SCN5A	3_38639443_C_T	ENSG00000183873:ENST00000414099:exon13:c.G2039A:p.R680H,ENSG00000183873:ENST00000423572:exon13:c.G2039A:p.R680H,ENSG00000183873:ENST00000449557:exon13:c.G2039A:p.R680H,ENSG00000183873:ENST00000450102:exon13:c.G2039A:p.R680H,ENSG00000183873:ENST00000455624:exon13:c.G2039A:p.R680H,ENSG00000183873:ENST00000333535:exon14:c.G2039A:p.R680H,ENSG00000183873:ENST00000413689:exon14:c.G2039A:p.R680H,ENSG00000183873:ENST00000425664:exon14:c.G2039A:p.R680H,ENSG00000183873:ENST00000443581:exon14:c.G2039A:p.R680H,ENSG00000183873:ENST00000451551:exon14:c.G2039A:p.R680H	rs199473142	D;P	0,00061
SCN5A	3_38640418_C_T	ENSG00000183873:ENST00000414099:exon12:c.G2014A:p.A672T,ENSG00000183873:ENST00000423572:exon12:c.G2014A:p.A672T,ENSG00000183873:ENST00000449557:exon12:c.G2014A:p.A672T,ENSG00000183873:ENST00000450102:exon12:c.G2014A:p.A672T,ENSG00000183873:ENST00000455624:exon12:c.G2014A:p.A672T,ENSG00000183873:ENST00000333535:exon13:c.G2014A:p.A672T,ENSG00000183873:ENST00000413689:exon13:c.G2014A:p.A672T,ENSG00000183873:ENST00000425664:exon13:c.G2014A:p.A672T,ENSG00000183873:ENST00000443581:exon13:c.G2014A:p.A672T,ENSG00000183873:ENST00000451551:exon13:c.G2014A:p.A672T	rs199473140	D;P	0,00062
SCN5A	3_38640450_C_T	ENSG00000183873:ENST00000414099:exon12:c.G1982A:p.R661Q,ENSG00000183873:ENST00000423572:exon12:c.G1982A:p.R661Q,ENSG00000183873:ENST00000449557:exon12:c.G1982A:p.R661Q,ENSG00000183873:ENST00000450102:exon12:c.G1982A:p.R661Q,ENSG00000183873:ENST00000455624:exon12:c.G1982A:p.R661Q,ENSG00000183873:ENST00000333535:exon13:c.G1982A:p.R661Q,ENSG00000183873:ENST00000413689:exon13:c.G1982A:p.R661Q,ENSG00000183873:ENST00000425664:exon13:c.G1982A:p.R661Q,ENSG00000183873:ENST00000443581:exon13:c.G1982A:p.R661Q,ENSG00000183873:ENST00000451551:exon13:c.G1982A:p.R661Q		N	0,00067
SCN5A	3_38645332_GAGGGC_-	ENSG00000183873:ENST00000414099:exon11:c.1756_1761del:p.586_587del,ENSG00000183873:ENST00000423572:exon11:c.1756_1761del:p.586_587del,ENSG00000183873:ENST00000449557:exon11:c.1756_1761del:p.586_587del,ENSG00000183873:ENST00000450102:exon11:c.1756_1761del:p.586_587del,ENSG00000183873:ENST00000455624:exon11:c.1756_1761del:p.586_587del,ENSG00000183873:ENST00000333535:exon12:c.1756_1761del:p.586_587del,ENSG00000183873:ENST00000413689:exon12:c.1756_1761del:p.586_587del,ENSG00000183873:ENST00000425664:exon12:c.1756_1761del:p.586_587del,ENSG00000183873:ENST00000443581:exon12:c.1756_1761del:p.586_587del,ENSG00000183873:ENST00000451551:exon12:c.1756_1761del:p.586_587del		N	0,00062

SCN5A	3_38645333_AGGGCG_-	ENSG00000183873:ENST00000414099:exon11:c.1755_1760del:p.585_587del,ENSG00000183873:ENST00000423572:exon11:c.1755_1760del:p.585_587del,ENSG00000183873:ENST00000449557:exon11:c.1755_1760del:p.585_587del,ENSG00000183873:ENST00000450102:exon11:c.1755_1760del:p.585_587del,ENSG00000183873:ENST00000455624:exon11:c.1755_1760del:p.585_587del,ENSG00000183873:ENST00000333535:exon12:c.1755_1760del:p.585_587del,ENSG00000183873:ENST00000413689:exon12:c.1755_1760del:p.585_587del,ENSG00000183873:ENST00000425664:exon12:c.1755_1760del:p.585_587del,ENSG00000183873:ENST00000443581:exon12:c.1755_1760del:p.585_587del,ENSG00000183873:ENST00000451551:exon12:c.1755_1760del:p.585_587del		P	0,00062
SCN5A	3_38645438_C_G	ENSG00000183873:ENST00000414099:exon11:c.G1655C:p.G552A,ENSG00000183873:ENST00000423572:exon11:c.G1655C:p.G552A,ENSG00000183873:ENST00000449557:exon11:c.G1655C:p.G552A,ENSG00000183873:ENST00000450102:exon11:c.G1655C:p.G552A,ENSG00000183873:ENST00000455624:exon11:c.G1655C:p.G552A,ENSG00000183873:ENST00000333535:exon12:c.G1655C:p.G552A,ENSG00000183873:ENST00000413689:exon12:c.G1655C:p.G552A,ENSG00000183873:ENST00000425664:exon12:c.G1655C:p.G552A,ENSG00000183873:ENST00000443581:exon12:c.G1655C:p.G552A,ENSG00000183873:ENST00000451551:exon12:c.G1655C:p.G552A		N	0,00061
SCN5A	3_38646398_G_C	ENSG00000183873:ENST00000414099:exon10:c.C1340G:p.A447G,ENSG00000183873:ENST00000423572:exon10:c.C1340G:p.A447G,ENSG00000183873:ENST00000449557:exon10:c.C1340G:p.A447G,ENSG00000183873:ENST00000450102:exon10:c.C1340G:p.A447G,ENSG00000183873:ENST00000455624:exon10:c.C1340G:p.A447G,ENSG00000183873:ENST00000333535:exon11:c.C1340G:p.A447G,ENSG00000183873:ENST00000413689:exon11:c.C1340G:p.A447G,ENSG00000183873:ENST00000425664:exon11:c.C1340G:p.A447G,ENSG00000183873:ENST00000443581:exon11:c.C1340G:p.A447G,ENSG00000183873:ENST00000451551:exon11:c.C1340G:p.A447G	rs199473113	D	0,00057
SCN5A	3_38647444_C_T	ENSG00000183873:ENST00000414099:exon9:c.G1336A:p.E446K,ENSG00000183873:ENST00000423572:exon9:c.G1336A:p.E446K,ENSG00000183873:ENST00000449557:exon9:c.G1336A:p.E446K,ENSG00000183873:ENST00000450102:exon9:c.G1336A:p.E446K,ENSG00000183873:ENST00000455624:exon9:c.G1336A:p.E446K,ENSG00000183873:ENST00000333535:exon10:c.G1336A:p.E446K,ENSG00000183873:ENST00000413689:exon10:c.G1336A:p.E446K,ENSG00000183873:ENST00000425664:exon10:c.G1336A:p.E446K,ENSG00000183873:ENST00000443581:exon10:c.G1336A:p.E446K,ENSG00000183873:ENST00000451551:exon10:c.G1336A:p.E446K	rs199473339	D;P	0,00115
SCN5A	3_38655278_G_A	ENSG00000183873:ENST00000423572:exon5:c.C659T:p.T220I,ENSG00000183873:ENST00000449557:exon5:c.C659T:p.T220I,ENSG00000183873:ENST00000333535:exon6:c.C659T:p.T220I,ENSG00000183873:ENST00000443581:exon6:c.C659T:p.T220I	rs45620037	D;P	0,00116
SCN5A	3_38655318_T_C	ENSG00000183873:ENST00000423572:exon5:c.A619G:p.T207A,ENSG00000183873:ENST00000449557:exon5:c.A619G:p.T207A,ENSG00000183873:ENST00000333535:exon6:c.A619G:p.T207A,ENSG00000183873:ENST00000443581:exon6:c.A619G:p.T207A		N	0,00058

SCN5A	3_38662449_C_T	ENSG00000183873:ENST00000414099:exon4:c.G496A:p.A166T,ENSG00000183873:ENST00000423572:exon4:c.G496A:p.A166T,ENSG00000183873:ENST00000449557:exon4:c.G496A:p.A166T,ENSG00000183873:ENST00000450102:exon4:c.G496A:p.A166T,ENSG00000183873:ENST00000455624:exon4:c.G496A:p.A166T,ENSG00000183873:ENST00000333535:exon5:c.G496A:p.A166T,ENSG00000183873:ENST00000413689:exon5:c.G496A:p.A166T,ENSG00000183873:ENST00000425664:exon5:c.G496A:p.A166T,ENSG00000183873:ENST00000443581:exon5:c.G496A:p.A166T,ENSG00000183873:ENST00000451551:exon5:c.G496A:p.A166T	rs201232332	D;S	0,00060
SCN5A	3_38671821_C_G	ENSG00000183873:ENST00000414099:exon2:c.G373C:p.V125L,ENSG00000183873:ENST00000423572:exon2:c.G373C:p.V125L,ENSG00000183873:ENST00000449557:exon2:c.G373C:p.V125L,ENSG00000183873:ENST00000450102:exon2:c.G373C:p.V125L,ENSG00000183873:ENST00000455624:exon2:c.G373C:p.V125L,ENSG00000183873:ENST00000333535:exon3:c.G373C:p.V125L,ENSG00000183873:ENST00000413689:exon3:c.G373C:p.V125L,ENSG00000183873:ENST00000425664:exon3:c.G373C:p.V125L,ENSG00000183873:ENST00000443581:exon3:c.G373C:p.V125L,ENSG00000183873:ENST00000451551:exon3:c.G373C:p.V125L	rs199473059	D;P	0,00115
SCN5A	3_38674747_G_A	ENSG00000183873:ENST00000414099:exon1:c.C52T:p.R18W,ENSG00000183873:ENST00000423572:exon1:c.C52T:p.R18W,ENSG00000183873:ENST00000449557:exon1:c.C52T:p.R18W,ENSG00000183873:ENST00000450102:exon1:c.C52T:p.R18W,ENSG00000183873:ENST00000455624:exon1:c.C52T:p.R18W,ENSG00000183873:ENST00000327956:exon2:c.C52T:p.R18W,ENSG00000183873:ENST00000333535:exon2:c.C52T:p.R18W,ENSG00000183873:ENST00000413689:exon2:c.C52T:p.R18W,ENSG00000183873:ENST00000425664:exon2:c.C52T:p.R18W,ENSG00000183873:ENST00000443581:exon2:c.C52T:p.R18W,ENSG00000183873:ENST00000451551:exon2:c.C52T:p.R18W	rs199473044	D;P	0,00128
TCAP	17_37821616_G_A	ENSG00000173991:ENST00000309889:exon1:c.G4A:p.A2T,ENSG00000173991:ENST00000578283:exon1:c.G4A:p.A2T		N	0,00062
TCAP	17_37821644_C_T	ENSG00000173991:ENST00000309889:exon1:c.C32T:p.S11L,ENSG00000173991:ENST00000578283:exon1:c.C32T:p.S11L	rs45495192	D	0,00126
TCAP	17_37821645_GGA_-	ENSG00000173991:ENST00000309889:exon1:c.33_35del:p.11_12del,ENSG00000173991:ENST00000578283:exon1:c.33_35del:p.11_12del		N	0,00063
TCAP	17_37821649_GAG_-	ENSG00000173991:ENST00000309889:exon1:c.37_39del:p.13_13del,ENSG00000173991:ENST00000578283:exon1:c.37_39del:p.13_13del		P	0,00063
TCAP	17_37822175_G_T	ENSG00000173991:ENST00000309889:exon2:c.G317T:p.R106L,ENSG00000173991:ENST00000578283:exon3:c.G245T:p.R82L		N	0,00092
TCAP	17_37822195_C_T	ENSG00000173991:ENST00000309889:exon2:c.C337T:p.L113F,ENSG00000173991:ENST00000578283:exon3:c.C265T:p.L89F		N	0,00091
TCAP	17_37822211_C_T	ENSG00000173991:ENST00000309889:exon2:c.C353T:p.A118V,ENSG00000173991:ENST00000578283:exon3:c.C281T:p.A94V	rs143233087	D	0,00090
TGFB3	14_76429772_C_G	ENSG00000119699:ENST00000238682:exon5:c.G813C:p.K271N,ENSG00000119699:ENST00000556285:exon5:c.G813C:p.K271N	rs147601018	D	0,00058

TGFB3	14_76437523_C_T	ENSG00000119699:ENST00000238682:exon3:c.G592A:p.E198K,ENSG00000119699:ENST00000556285:exon3:c.G592A:p.E198K		N	0,00058
TGFB3	14_76437535_G_A	ENSG00000119699:ENST00000238682:exon3:c.C580T:p.R194W,ENSG00000119699:ENST00000556285:exon3:c.C580T:p.R194W		S	0,00058
TGFB3	14_76438059_C_T	ENSG00000119699:ENST00000238682:exon2:c.G355A:p.E119K,ENSG00000119699:ENST00000556285:exon2:c.G355A:p.E119K		N	0,00059
TGFB3	14_76446944_G_A	ENSG00000119699:ENST00000238682:exon1:c.C293T:p.S98L,ENSG00000119699:ENST00000556285:exon1:c.C293T:p.S98L	rs142047577	D	0,00059
TMEM43	3_14170982_G_A	ENSG00000170876:ENST00000306077:exon2:c.G83A:p.R28Q		S	0,00058
TMEM43	3_14170990_G_A	ENSG00000170876:ENST00000306077:exon2:c.G91A:p.E31K		N	0,00058
TMEM43	3_14172439_G_A	ENSG00000170876:ENST00000306077:exon3:c.G280A:p.A94T		N	0,00088
TMEM43	3_14175244_T_C	ENSG00000170876:ENST00000306077:exon7:c.T518C:p.M173T		S	0,00093
TMEM43	3_14176287_G_A	ENSG00000170876:ENST00000306077:exon8:c.G601A:p.D201N	rs138182276	D	0,00063
TMEM43	3_14177355_A_T	ENSG00000170876:ENST00000306077:exon10:c.A829T:p.T277S		N	0,00059
TNNC1	3_52485431_T_C	ENSG00000114854:ENST00000232975:exon5:c.A430G:p.N144D		N	0,00060
TNNC1	3_52485805_C_T	ENSG00000114854:ENST00000496590:exon3:c.G140A:p.G47E,ENSG00000114854:ENST00000232975:exon4:c.G272A:p.G91E		S	0,00057
TNNC1	3_52485879_G_A	ENST00000232975:exon5:c.203-5C>T,ENST00000496590:exon4:c.71-5C>T	rs142519988	D;L	0,00058
TNNC1	3_52486194_C_T	ENSG00000114854:ENST00000232975:exon3:c.G130A:p.V44M		S	0,00063
TNNI3	19_55663243_G_C	ENSG00000129991:ENST00000588882:exon5:c.C517G:p.L173V,ENSG00000129991:ENST00000344887:exon8:c.C592G:p.L198V		P	0,00057
TNNI3	19_55663249_C_T	ENSG00000129991:ENST00000588882:exon5:c.G511A:p.D171N,ENSG00000129991:ENST00000344887:exon8:c.G586A:p.D196N	rs104894727	D;P	0,00115
TNNI3	19_55663278_C_T	ENSG00000129991:ENST00000588882:exon5:c.G482A:p.R161Q,ENSG00000129991:ENST00000344887:exon8:c.G557A:p.R186Q		P	0,00057
TNNI3	19_55665421_C_T	ENSG00000129991:ENST00000588882:exon4:c.G451A:p.V151M,ENSG00000129991:ENST00000344887:exon7:c.G526A:p.V176M		S	0,00115
TNNI3	19_55665436_C_T	ENSG00000129991:ENST00000588882:exon4:c.G436A:p.A146T,ENSG00000129991:ENST00000344887:exon7:c.G511A:p.A171T	rs121917761	D;P	0,00057
TNNI3	19_55665462_C_T	ENSG00000129991:ENST00000588882:exon4:c.G410A:p.R137Q,ENSG00000129991:ENST00000344887:exon7:c.G485A:p.R162Q		P	0,00173
TNNI3	19_55665463_G_A	ENSG00000129991:ENST00000588882:exon4:c.C409T:p.R137W,ENSG00000129991:ENST00000344887:exon7:c.C484T:p.R162W		P	0,00115

TNNI3	19_55665477_G_A	ENSG00000129991:ENST00000588882:exon4:c.C395T:p.A132V,ENSG00000129991:ENST00000344887:exon7:c.C470T:p.A157V		P	0,00057
TNNI3	19_55665514_G_A	ENSG00000129991:ENST00000588882:exon4:c.C358T:p.R120W,ENSG00000129991:ENST00000344887:exon7:c.C433T:p.R145W	rs104894724	D;P	0,00229
TNNI3	19_55665525_C_T	ENSG00000129991:ENST00000588882:exon4:c.G347A:p.R116Q,ENSG00000129991:ENST00000344887:exon7:c.G422A:p.R141Q		P	0,00057
TNNI3	19_55667616_G_A	ENSG00000129991:ENST00000588882:exon2:c.C160T:p.R54C,ENSG00000129991:ENST00000344887:exon5:c.C235T:p.R79C	rs3729712	D	0,00082
TNNI3	19_55668007_T_A	ENSG00000129991:ENST00000586858:exon1:c.A39T:p.K13N,ENSG00000129991:ENST00000588882:exon1:c.A39T:p.K13N,ENSG00000129991:ENST00000344887:exon4:c.A114T:p.K38N		S	0,00062
TNNI3	19_55668029_G_T	ENSG00000129991:ENST00000586858:exon1:c.C17A:p.S6Y,ENSG00000129991:ENST00000588882:exon1:c.C17A:p.S6Y	rs139150276	D	0,00245
TNNT2	1_201328345_C_T	ENSG00000118194:ENST00000360372:exon14:c.G845A:p.W282X,ENSG00000118194:ENST00000367315:exon14:c.G851A:p.W284X,ENSG00000118194:ENST00000236918:exon15:c.G875A:p.W292X,ENSG00000118194:ENST00000367317:exon15:c.G860A:p.W287X,ENSG00000118194:ENST00000367320:exon15:c.G761A:p.W254X,ENSG00000118194:ENST00000367322:exon15:c.G851A:p.W284X,ENSG00000118194:ENST00000421663:exon15:c.G869A:p.W290X,ENSG00000118194:ENST00000367318:exon16:c.G860A:p.W287X,ENSG00000118194:ENST00000458432:exon16:c.G887A:p.W296X,ENSG00000118194:ENST00000509001:exon16:c.G860A:p.W287X		P	0,00061
TNNT2	1_201328348_C_T	ENSG00000118194:ENST00000360372:exon14:c.G842A:p.R281H,ENSG00000118194:ENST00000367315:exon14:c.G848A:p.R283H,ENSG00000118194:ENST00000236918:exon15:c.G872A:p.R291H,ENSG00000118194:ENST00000367317:exon15:c.G857A:p.R286H,ENSG00000118194:ENST00000367320:exon15:c.G758A:p.R253H,ENSG00000118194:ENST00000367322:exon15:c.G848A:p.R283H,ENSG00000118194:ENST00000421663:exon15:c.G866A:p.R289H,ENSG00000118194:ENST00000367318:exon16:c.G857A:p.R286H,ENSG00000118194:ENST00000458432:exon16:c.G884A:p.R295H,ENSG00000118194:ENST00000509001:exon16:c.G857A:p.R286H	rs141121678	D;P	0,00122
TNNT2	1_201328372_C_G	ENSG00000118194:ENST00000360372:exon14:c.G818C:p.R273P,ENSG00000118194:ENST00000367315:exon14:c.G824C:p.R275P,ENSG00000118194:ENST00000236918:exon15:c.G848C:p.R283P,ENSG00000118194:ENST00000367317:exon15:c.G833C:p.R278P,ENSG00000118194:ENST00000367320:exon15:c.G734C:p.R245P,ENSG00000118194:ENST00000367322:exon15:c.G824C:p.R275P,ENSG00000118194:ENST00000421663:exon15:c.G842C:p.R281P,ENSG00000118194:ENST00000367318:exon16:c.G833C:p.R278P,ENSG00000118194:ENST00000458432:exon16:c.G860C:p.R287P,ENSG00000118194:ENST00000509001:exon16:c.G833C:p.R278P		N	0,00062

TNNT2	1_201328373_G_A	ENSG00000118194:ENST00000360372:exon14:c.C817T:p.R273C,ENSG00000118194:ENST00000367315:exon14:c.C823T:p.R275C,ENSG00000118194:ENST00000236918:exon15:c.C847T:p.R283C,ENSG00000118194:ENST00000367317:exon15:c.C832T:p.R278C,ENSG00000118194:ENST00000367320:exon15:c.C733T:p.R245C,ENSG00000118194:ENST00000367322:exon15:c.C823T:p.R275C,ENSG00000118194:ENST00000421663:exon15:c.C841T:p.R281C,ENSG00000118194:ENST00000367318:exon16:c.C832T:p.R278C,ENSG00000118194:ENST00000458432:exon16:c.C859T:p.R287C,ENSG00000118194:ENST00000509001:exon16:c.C832T:p.R278C	rs121964857	D;P	0,00498
TNNT2	1_201328386_G_T	ENST00000367322:exon16:c.813-3C>A,ENST00000367318:exon17:c.822-3C>A,ENST00000360372:exon15:c.807-3C>A,ENST00000367315:exon15:c.813-3C>A,ENST00000367317:exon16:c.822-3C>A,ENST00000236918:exon16:c.837-3C>A,ENST00000421663:exon16:c.831-3C>A,ENST00000458432:exon17:c.849-3C>A,ENST00000367320:exon16:c.723-3C>A,ENST00000509001:exon17:c.822-3C>A,ENST00000438742:exon16:c.804-3C>A		L	0,00062
TNNT2	1_201328760_T_A	ENSG00000118194:ENST00000360372:exon13:c.A797T:p.N266I,ENSG00000118194:ENST00000367315:exon13:c.A803T:p.N268I,ENSG00000118194:ENST00000236918:exon14:c.A827T:p.N276I,ENSG00000118194:ENST00000367317:exon14:c.A812T:p.N271I,ENSG00000118194:ENST00000367320:exon14:c.A713T:p.N238I,ENSG00000118194:ENST00000367322:exon14:c.A803T:p.N268I,ENSG00000118194:ENST00000421663:exon14:c.A821T:p.N274I,ENSG00000118194:ENST00000438742:exon14:c.A794T:p.N265I,ENSG00000118194:ENST00000367318:exon15:c.A812T:p.N271I,ENSG00000118194:ENST00000458432:exon15:c.A839T:p.N280I,ENSG00000118194:ENST00000509001:exon15:c.A812T:p.N271I		P	0,00059
TNNT2	1_201328787_T_C	ENSG00000118194:ENST00000360372:exon13:c.A770G:p.N257S,ENSG00000118194:ENST00000367315:exon13:c.A776G:p.N259S,ENSG00000118194:ENST00000236918:exon14:c.A800G:p.N267S,ENSG00000118194:ENST00000367317:exon14:c.A785G:p.N262S,ENSG00000118194:ENST00000367320:exon14:c.A686G:p.N229S,ENSG00000118194:ENST00000367322:exon14:c.A776G:p.N259S,ENSG00000118194:ENST00000421663:exon14:c.A794G:p.N265S,ENSG00000118194:ENST00000438742:exon14:c.A767G:p.N256S,ENSG00000118194:ENST00000367318:exon15:c.A785G:p.N262S,ENSG00000118194:ENST00000458432:exon15:c.A812G:p.N271S,ENSG00000118194:ENST00000509001:exon15:c.A785G:p.N262S		P	0,00117
TNNT2	1_201331150_T_C	ENSG00000118194:ENST00000360372:exon11:c.A565G:p.T189A,ENSG00000118194:ENST00000367315:exon11:c.A571G:p.T191A,ENSG00000118194:ENST00000236918:exon12:c.A595G:p.T199A,ENSG00000118194:ENST00000367317:exon12:c.A580G:p.T194A,ENSG00000118194:ENST00000367320:exon12:c.A481G:p.T161A,ENSG00000118194:ENST00000367322:exon12:c.A571G:p.T191A,ENSG00000118194:ENST00000421663:exon12:c.A589G:p.T197A,ENSG00000118194:ENST00000438742:exon12:c.A562G:p.T188A,ENSG00000118194:ENST00000367318:exon13:c.A580G:p.T194A,ENSG00000118194:ENST00000458432:exon13:c.A607G:p.T203A,ENSG00000118194:ENST00000509001:exon13:c.A580G:p.T194A		N	0,00058

TNNT2	1_201332459_A_C	ENSG00000118194:ENST00000360372:exon9:c.T520G:p.S174A,ENSG00000118194:ENST00000236918:exon10:c.T550G:p.S184A,ENSG00000118194:ENST00000367315:exon10:c.T535G:p.S179A,ENSG00000118194:ENST00000367317:exon10:c.T535G:p.S179A,ENSG00000118194:ENST00000438742:exon10:c.T520G:p.S174A,ENSG00000118194:ENST00000367318:exon11:c.T535G:p.S179A,ENSG00000118194:ENST00000367320:exon11:c.T445G:p.S149A,ENSG00000118194:ENST00000367322:exon11:c.T535G:p.S179A,ENSG00000118194:ENST00000421663:exon11:c.T541G:p.S181A,ENSG00000118194:ENST00000509001:exon11:c.T535G:p.S179A,ENSG00000118194:ENST00000458432:exon12:c.T571G:p.S191A		N	0,00057
TNNT2	1_201332505_CTC_-	ENSG00000118194:ENST00000360372:exon9:c.472_474del:p.158_158del,ENSG00000118194:ENST00000236918:exon10:c.502_504del:p.168_168del,ENSG00000118194:ENST00000367315:exon10:c.487_489del:p.163_163del,ENSG00000118194:ENST00000367317:exon10:c.487_489del:p.163_163del,ENSG00000118194:ENST00000438742:exon10:c.472_474del:p.158_158del,ENSG00000118194:ENST00000367318:exon11:c.487_489del:p.163_163del,ENSG00000118194:ENST00000367320:exon11:c.397_399del:p.133_133del,ENSG00000118194:ENST00000367322:exon11:c.487_489del:p.163_163del,ENSG00000118194:ENST00000421663:exon11:c.493_495del:p.165_165del,ENSG00000118194:ENST00000509001:exon11:c.487_489del:p.163_163del,ENSG00000118194:ENST00000455702:exon12:c.517_519del:p.173_173del,ENSG00000118194:ENST00000458432:exon12:c.523_525del:p.175_175del		P	0,00172
TNNT2	1_201332514_CTC_-	ENSG00000118194:ENST00000360372:exon9:c.463_465del:p.155_155del,ENSG00000118194:ENST00000236918:exon10:c.493_495del:p.165_165del,ENSG00000118194:ENST00000367315:exon10:c.478_480del:p.160_160del,ENSG00000118194:ENST00000367317:exon10:c.478_480del:p.160_160del,ENSG00000118194:ENST00000438742:exon10:c.463_465del:p.155_155del,ENSG00000118194:ENST00000367318:exon11:c.478_480del:p.160_160del,ENSG00000118194:ENST00000367320:exon11:c.388_390del:p.130_130del,ENSG00000118194:ENST00000367322:exon11:c.478_480del:p.160_160del,ENSG00000118194:ENST00000421663:exon11:c.484_486del:p.162_162del,ENSG00000118194:ENST00000509001:exon11:c.478_480del:p.160_160del,ENSG00000118194:ENST00000455702:exon12:c.508_510del:p.170_170del,ENSG00000118194:ENST00000458432:exon12:c.514_516del:p.172_172del		P	0,00172
TNNT2	1_201334389_G_A	ENSG00000118194:ENST00000360372:exon7:c.C296T:p.A99V,ENSG00000118194:ENST00000236918:exon8:c.C326T:p.A109V,ENSG00000118194:ENST00000367315:exon8:c.C311T:p.A104V,ENSG00000118194:ENST00000367317:exon8:c.C311T:p.A104V,ENSG00000118194:ENST00000438742:exon8:c.C296T:p.A99V,ENSG00000118194:ENST00000367318:exon9:c.C311T:p.A104V,ENSG00000118194:ENST00000367322:exon9:c.C311T:p.A104V,ENSG00000118194:ENST00000421663:exon9:c.C317T:p.A106V,ENSG00000118194:ENST00000422165:exon9:c.C326T:p.A109V,ENSG00000118194:ENST00000509001:exon9:c.C311T:p.A104V,ENSG00000118194:ENST00000455702:exon10:c.C341T:p.A114V,ENSG00000118194:ENST00000458432:exon10:c.C347T:p.A116V		P	0,00174

TNNT2	1_201334419_C_T	ENSG00000118194:ENST00000360372:exon7:c.G266A:p.R89H,ENSG00000118194:ENST00000236918:exon8:c.G296A:p.R99H,ENSG00000118194:ENST00000367315:exon8:c.G281A:p.R94H,ENSG00000118194:ENST00000367317:exon8:c.G281A:p.R94H,ENSG00000118194:ENST00000438742:exon8:c.G266A:p.R89H,ENSG00000118194:ENST00000367318:exon9:c.G281A:p.R94H,ENSG00000118194:ENST00000367322:exon9:c.G281A:p.R94H,ENSG00000118194:ENST00000412633:exon9:c.G278A:p.R93H,ENSG00000118194:ENST00000421663:exon9:c.G287A:p.R96H,ENSG00000118194:ENST00000422165:exon9:c.G296A:p.R99H,ENSG00000118194:ENST00000509001:exon9:c.G281A:p.R94H,ENSG00000118194:ENST00000455702:exon10:c.G311A:p.R104H,ENSG00000118194:ENST00000458432:exon10:c.G317A:p.R106H		P	0,00059
TNNT2	1_201334425_C_T	ENSG00000118194:ENST00000360372:exon7:c.G260A:p.R87Q,ENSG00000118194:ENST00000236918:exon8:c.G290A:p.R97Q,ENSG00000118194:ENST00000367315:exon8:c.G275A:p.R92Q,ENSG00000118194:ENST00000367317:exon8:c.G275A:p.R92Q,ENSG00000118194:ENST00000438742:exon8:c.G260A:p.R87Q,ENSG00000118194:ENST00000367318:exon9:c.G275A:p.R92Q,ENSG00000118194:ENST00000367322:exon9:c.G275A:p.R92Q,ENSG00000118194:ENST00000412633:exon9:c.G272A:p.R91Q,ENSG00000118194:ENST00000421663:exon9:c.G281A:p.R94Q,ENSG00000118194:ENST00000422165:exon9:c.G290A:p.R97Q,ENSG00000118194:ENST00000509001:exon9:c.G275A:p.R92Q,ENSG00000118194:ENST00000455702:exon10:c.G305A:p.R102Q,ENSG00000118194:ENST00000458432:exon10:c.G311A:p.R104Q	rs121964856	D;P	0,00059
TNNT2	1_201334426_G_A	ENSG00000118194:ENST00000360372:exon7:c.C259T:p.R87W,ENSG00000118194:ENST00000236918:exon8:c.C289T:p.R97W,ENSG00000118194:ENST00000367315:exon8:c.C274T:p.R92W,ENSG00000118194:ENST00000367317:exon8:c.C274T:p.R92W,ENSG00000118194:ENST00000438742:exon8:c.C259T:p.R87W,ENSG00000118194:ENST00000367318:exon9:c.C274T:p.R92W,ENSG00000118194:ENST00000367322:exon9:c.C274T:p.R92W,ENSG00000118194:ENST00000412633:exon9:c.C271T:p.R91W,ENSG00000118194:ENST00000421663:exon9:c.C280T:p.R94W,ENSG00000118194:ENST00000422165:exon9:c.C289T:p.R97W,ENSG00000118194:ENST00000509001:exon9:c.C274T:p.R92W,ENSG00000118194:ENST00000455702:exon10:c.C304T:p.R102W,ENSG00000118194:ENST00000458432:exon10:c.C310T:p.R104W		P	0,00118
TNNT2	1_201334766_A_T	ENSG00000118194:ENST00000360372:exon6:c.T221A:p.I74N,ENSG00000118194:ENST00000236918:exon7:c.T251A:p.I84N,ENSG00000118194:ENST00000367315:exon7:c.T236A:p.I79N,ENSG00000118194:ENST00000367317:exon7:c.T236A:p.I79N,ENSG00000118194:ENST00000438742:exon7:c.T221A:p.I74N,ENSG00000118194:ENST00000367318:exon8:c.T236A:p.I79N,ENSG00000118194:ENST00000367322:exon8:c.T236A:p.I79N,ENSG00000118194:ENST00000412633:exon8:c.T233A:p.I78N,ENSG00000118194:ENST00000421663:exon8:c.T242A:p.I81N,ENSG00000118194:ENST00000422165:exon8:c.T251A:p.I84N,ENSG00000118194:ENST00000509001:exon8:c.T236A:p.I79N,ENSG00000118194:ENST00000367320:exon9:c.T263A:p.I88N,ENSG00000118194:ENST00000455702:exon9:c.T266A:p.I89N,ENSG00000118194:ENST00000458432:exon9:c.T272A:p.I91N	rs121964855	D;P	0,00058

TNNT2	1_201337340_G_A	ENSG00000118194:ENST00000360372:exon3:c.C68T:p.A23V,ENSG00000118194:ENST00000236918:exon4:c.C98T:p.A33V,ENSG00000118194:ENST00000367315:exon4:c.C83T:p.A28V,ENSG00000118194:ENST00000367317:exon4:c.C83T:p.A28V,ENSG00000118194:ENST00000438742:exon4:c.C68T:p.A23V,ENSG00000118194:ENST00000367318:exon5:c.C83T:p.A28V,ENSG00000118194:ENST00000367322:exon5:c.C83T:p.A28V,ENSG00000118194:ENST00000412633:exon5:c.C83T:p.A28V,ENSG00000118194:ENST00000421663:exon5:c.C89T:p.A30V,ENSG00000118194:ENST00000422165:exon5:c.C98T:p.A33V,ENSG00000118194:ENST00000509001:exon5:c.C83T:p.A28V,ENSG00000118194:ENST00000367320:exon6:c.C113T:p.A38V,ENSG00000118194:ENST00000455702:exon6:c.C113T:p.A38V,ENSG00000118194:ENST00000458432:exon6:c.C119T:p.A40V	rs200754249	D;P	0,00058
TNNT2	1_201337541_G_A	ENST00000360372:exon3:c.52+5C>T		L	0,00058
TNNT2	1_201338976_G_-	ENST00000236918:exon4:c.52-2C>-,ENST00000458432:exon6:c.74-3C>-,ENST00000367320:exon6:c.68-3C>-,ENST00000455702:exon6:c.68-3C>-,ENST00000422165:exon5:c.53-3C>-	rs200153031	D	0,00184
TNNT2	1_201338976_G_A	ENST00000236918:exon4:c.52-2C>T,ENST00000458432:exon6:c.74-3C>T,ENST00000367320:exon6:c.68-3C>T,ENST00000455702:exon6:c.68-3C>T,ENST00000422165:exon5:c.53-3C>T		N	0,00122
TNNT2	1_201338978_G_A	ENST00000236918:exon4:c.52-4C>T,ENST00000458432:exon6:c.74-5C>T,ENST00000367320:exon6:c.68-5C>T,ENST00000455702:exon6:c.68-5C>T,ENST00000422165:exon5:c.53-5C>T		L	0,00061
TNNT2	1_201338979_G_-	ENST00000236918:exon4:c.52-5C>-		L	0,00183
TPM1	15_63335110_G_A	ENSG00000140416:ENST00000267996:exon1:c.G82A:p.D28N,ENSG00000140416:ENST00000288398:exon1:c.G82A:p.D28N,ENSG00000140416:ENST00000357980:exon1:c.G82A:p.D28N,ENSG00000140416:ENST00000358278:exon1:c.G82A:p.D28N,ENSG00000140416:ENST00000403994:exon1:c.G82A:p.D28N,ENSG00000140416:ENST00000558347:exon1:c.G82A:p.D28N,ENSG00000140416:ENST00000559397:exon1:c.G82A:p.D28N,ENSG00000140416:ENST00000559556:exon1:c.G82A:p.D28N,ENSG00000140416:ENST00000560445:exon1:c.G82A:p.D28N,ENSG00000140416:ENST00000561425:exon1:c.G82A:p.D28N		N	0,00187
TPM1	15_63335138_A_C	ENSG00000140416:ENST00000267996:exon1:c.A110C:p.K37T,ENSG00000140416:ENST00000288398:exon1:c.A110C:p.K37T,ENSG00000140416:ENST00000357980:exon1:c.A110C:p.K37T,ENSG00000140416:ENST00000358278:exon1:c.A110C:p.K37T,ENSG00000140416:ENST00000403994:exon1:c.A110C:p.K37T,ENSG00000140416:ENST00000558347:exon1:c.A110C:p.K37T,ENSG00000140416:ENST00000559397:exon1:c.A110C:p.K37T,ENSG00000140416:ENST00000559556:exon1:c.A110C:p.K37T,ENSG00000140416:ENST00000560445:exon1:c.A110C:p.K37T,ENSG00000140416:ENST00000561425:exon1:c.A110C:p.K37T		N	0,00094

TPM1	15_63336299_C_T	ENSG00000140416:ENST00000288398:exon2:c.C188T:p.A63V,ENSG00000140416:ENST00000358278:exon2:c.C188T:p.A63V,ENSG00000140416:ENST00000403994:exon2:c.C188T:p.A63V,ENSG00000140416:ENST00000558347:exon2:c.C188T:p.A63V,ENSG00000140416:ENST00000559556:exon2:c.C188T:p.A63V,ENSG00000140416:ENST00000357980:exon3:c.C314T:p.A105V	rs199476306	D;P	0,00057
TPM1	15_63351757_T_C	ENST00000288398:exon4:c.375-5T>C,ENST00000358278:exon4:c.375-5T>C,ENST00000403994:exon4:c.375-5T>C,ENST00000357980:exon5:c.501-5T>C,ENST00000267996:exon4:c.375-5T>C,ENST00000559397:exon4:c.375-5T>C,ENST00000559556:exon4:c.375-5T>C,ENST00000560970:exon4:c.317-5T>C,ENST00000561266:exon3:c.191-5T>C,ENST00000559831:exon3:c.147-5T>C,ENST00000334895:exon3:c.267-5T>C,ENST00000404484:exon3:c.267-5T>C,ENST00000558544:exon2:c.133-5T>C,ENST00000560959:exon3:c.267-5T>C,ENST00000317516:exon3:c.267-5T>C,ENST00000559281:exon3:c.267-5T>C,ENST00000561395:exon2:c.114-5T>C		P	0,00057
TPM1	15_63353123_C_T	ENSG00000140416:ENST00000558264:exon3:c.C170T:p.A57V,ENSG00000140416:ENST00000317516:exon4:c.C440T:p.A147V,ENSG00000140416:ENST00000334895:exon4:c.C440T:p.A147V,ENSG00000140416:ENST00000404484:exon4:c.C440T:p.A147V,ENSG00000140416:ENST00000559281:exon4:c.C440T:p.A147V,ENSG00000140416:ENST00000559831:exon4:c.C320T:p.A107V,ENSG00000140416:ENST00000560615:exon4:c.C170T:p.A57V,ENSG00000140416:ENST00000560959:exon4:c.C440T:p.A147V,ENSG00000140416:ENST00000267996:exon5:c.C548T:p.A183V,ENSG00000140416:ENST00000288398:exon5:c.C548T:p.A183V,ENSG00000140416:ENST00000358278:exon5:c.C548T:p.A183V,ENSG00000140416:ENST00000403994:exon5:c.C548T:p.A183V,ENSG00000140416:ENST00000559397:exon5:c.C548T:p.A183V,ENSG00000140416:ENST00000559556:exon5:c.C548T:p.A183V,ENSG00000140416:ENST00000357980:exon6:c.C674T:p.A225V		S	0,00058
TPM1	15_63353922_G_A	ENSG00000140416:ENST00000561395:exon3:c.G200A:p.R67Q,ENSG00000140416:ENST00000334895:exon5:c.G466A:p.E156K,ENSG00000140416:ENST00000558264:exon5:c.G272A:p.R91Q,ENSG00000140416:ENST00000559281:exon5:c.G466A:p.E156K,ENSG00000140416:ENST00000267996:exon6:c.G574A:p.E192K,ENSG00000140416:ENST00000403994:exon6:c.G574A:p.E192K,ENSG00000140416:ENST00000559556:exon6:c.G574A:p.E192K,ENSG00000140416:ENST00000559831:exon6:c.G422A:p.R141Q,ENSG00000140416:ENST00000357980:exon7:c.G700A:p.E234K	rs199476315	D;P	0,00115
TPM1	15_63354818_T_G	ENSG00000140416:ENST00000317516:exon7:c.T638G:p.L213W,ENSG00000140416:ENST00000334895:exon7:c.T638G:p.L213W,ENSG00000140416:ENST00000404484:exon7:c.T638G:p.L213W,ENSG00000140416:ENST00000559281:exon7:c.T638G:p.L213W,ENSG00000140416:ENST00000560959:exon7:c.T638G:p.L213W,ENSG00000140416:ENST00000267996:exon8:c.T746G:p.L249W,ENSG00000140416:ENST00000288398:exon8:c.T746G:p.L249W,ENSG00000140416:ENST00000358278:exon8:c.T746G:p.L249W,ENSG00000140416:ENST00000403994:exon8:c.T746G:p.L249W,ENSG00000140416:ENST00000559397:exon8:c.T746G:p.L249W,ENSG00000140416:ENST00000559556:exon8:c.T746G:p.L249W,ENSG00000140416:ENST00000357980:exon9:c.T872G:p.L291W		S	0,00057

TPM1	15_63354833_A_G	ENSG00000140416:ENST00000317516:exon7:c.A653G:p.D218G,ENSG00000140416:ENST00000334895:exon7:c.A653G:p.D218G,ENSG00000140416:ENST00000404484:exon7:c.A653G:p.D218G,ENSG00000140416:ENST00000559281:exon7:c.A653G:p.D218G,ENSG00000140416:ENST00000560959:exon7:c.A653G:p.D218G,ENSG00000140416:ENST00000267996:exon8:c.A761G:p.D254G,ENSG00000140416:ENST00000288398:exon8:c.A761G:p.D254G,ENSG00000140416:ENST00000358278:exon8:c.A761G:p.D254G,ENSG00000140416:ENST00000403994:exon8:c.A761G:p.D254G,ENSG00000140416:ENST00000559397:exon8:c.A761G:p.D254G,ENSG00000140416:ENST00000559556:exon8:c.A761G:p.D254G,ENSG00000140416:ENST00000357980:exon9:c.A887G:p.D296G		S	0,00057
TPM1	15_63354907_A_G	ENSG00000140416:ENST00000560959:exon7:c.A727G:p.I243V		N	0,00057
TPM1	15_63354938_CGCT_-	ENSG00000140416:ENST00000560959:exon7:c.758_761del:p.253_254del		L	0,00114
TPM1	15_63354943_C_T	ENSG00000140416:ENST00000560959:exon7:c.C763T:p.L255F	rs56054026	D	0,00114
TPM1	15_63356281_A_G	ENSG00000140416:ENST00000559281:exon8:c.A683G:p.K228R,ENSG00000140416:ENST00000288398:exon9:c.A791G:p.K264R,ENSG00000140416:ENST00000403994:exon9:c.A791G:p.K264R		S	0,00057
TPM1	15_63358166_A_G	ENSG00000140416:ENST00000560445:exon3:c.A193G:p.I65V		N	0,00057
VCL	10_75832551_G_A	ENSG00000035403:ENST00000211998:exon5:c.G563A:p.R188Q,ENSG00000035403:ENST00000372755:exon5:c.G563A:p.R188Q		N	0,00057
VCL	10_75832578_C_T	ENSG00000035403:ENST00000211998:exon5:c.C590T:p.T197I,ENSG00000035403:ENST00000372755:exon5:c.C590T:p.T197I	rs189242810	D;S	0,00057
VCL	10_75834522_C_T	ENSG00000035403:ENST00000211998:exon6:c.C644T:p.T215I,ENSG00000035403:ENST00000372755:exon6:c.C644T:p.T215I		N	0,00057
VCL	10_75849088_A_G	ENSG00000035403:ENST00000436396:exon2:c.A173G:p.K58R,ENSG00000035403:ENST00000211998:exon9:c.A1157G:p.K386R,ENSG00000035403:ENST00000372755:exon9:c.A1157G:p.K386R	rs200342284	D	0,00115
VCL	10_75849097_A_G	ENSG00000035403:ENST00000436396:exon2:c.A182G:p.D61G,ENSG00000035403:ENST00000211998:exon9:c.A1166G:p.D389G,ENSG00000035403:ENST00000372755:exon9:c.A1166G:p.D389G		N	0,00115
VCL	10_75849776_T_C	ENST00000211998:exon10:c.1177-5T>C,ENST00000372755:exon10:c.1177-5T>C,ENST00000436396:exon3:c.193-5T>C		L	0,00057
VCL	10_75849841_G_A	ENSG00000035403:ENST00000436396:exon3:c.G253A:p.A85T,ENSG00000035403:ENST00000211998:exon10:c.G1237A:p.A413T,ENSG00000035403:ENST00000372755:exon10:c.G1237A:p.A413T	rs146278697	D	0,00057
VCL	10_75855425_A_C	ENSG00000035403:ENST00000436396:exon5:c.A571C:p.I191L,ENSG00000035403:ENST00000211998:exon12:c.A1555C:p.I519L,ENSG00000035403:ENST00000372755:exon12:c.A1555C:p.I519L	rs141033098	D;S	0,00173
VCL	10_75855491_C_G	ENSG00000035403:ENST00000436396:exon5:c.C637G:p.L213V,ENSG00000035403:ENST00000211998:exon12:c.C1621G:p.L541V,ENSG00000035403:ENST00000372755:exon12:c.C1621G:p.L541V		N	0,00058
VCL	10_75857050_T_C	ENSG00000035403:ENST00000436396:exon6:c.T848C:p.V283A,ENSG00000035403:ENST00000211998:exon13:c.T1832C:p.V611A,ENSG00000035403:ENST00000372755:exon13:c.T1832C:p.V611A		N	0,00057

VCL	10_75857059_C_T	ENSG00000035403:ENST00000436396:exon6:c.C857T:p.T286M,ENSG00000035403:ENST00000211998:exon13:c.C1841T:p.T614M,ENSG00000035403:ENST00000372755:exon13:c.C1841T:p.T614M		S	0,00057
VCL	10_75860740_A_G	ENSG00000035403:ENST00000436396:exon7:c.A923G:p.H308R,ENSG00000035403:ENST00000211998:exon14:c.A1907G:p.H636R,ENSG00000035403:ENST00000372755:exon14:c.A1907G:p.H636R	rs71579374	D	0,00057
VCL	10_75860750_G_T	ENSG00000035403:ENST00000436396:exon7:c.G933T:p.K311N,ENSG00000035403:ENST00000211998:exon14:c.G1917T:p.K639N,ENSG00000035403:ENST00000372755:exon14:c.G1917T:p.K639N		S	0,00057
VCL	10_75860773_C_T	ENSG00000035403:ENST00000436396:exon7:c.C956T:p.A319V,ENSG00000035403:ENST00000211998:exon14:c.C1940T:p.A647V,ENSG00000035403:ENST00000372755:exon14:c.C1940T:p.A647V		N	0,00057
VCL	10_75860806_T_C	ENSG00000035403:ENST00000436396:exon7:c.T989C:p.V330A,ENSG00000035403:ENST00000211998:exon14:c.T1973C:p.V658A,ENSG00000035403:ENST00000372755:exon14:c.T1973C:p.V658A		N	0,00172
VCL	10_75871695_TAGGTG_-	ENSG00000035403:ENST00000436396:exon12:c.1790_1795del:p.597_599del,ENSG00000035403:ENST00000211998:exon19:c.2774_2779del:p.925_927del		N	0,00057
VCL	10_75871722_C_T	ENSG00000035403:ENST00000436396:exon12:c.C1817T:p.A606V,ENSG00000035403:ENST00000211998:exon19:c.C2801T:p.A934V	rs16931179	D;P	0,00057
VCL	10_75871748_C_G	ENSG00000035403:ENST00000436396:exon12:c.C1843G:p.P615A,ENSG00000035403:ENST00000211998:exon19:c.C2827G:p.P943A	rs71579375	D;P	0,00057
VCL	10_75871782_TGT_-	ENSG00000035403:ENST00000436396:exon12:c.1877_1879del:p.626_627del,ENSG00000035403:ENST00000211998:exon19:c.2861_2863del:p.954_955del		P	0,00057
VCL	10_75873951_A_G	ENSG00000035403:ENST00000417648:exon5:c.A538G:p.I180V,ENSG00000035403:ENST00000436396:exon13:c.A1975G:p.I659V,ENSG00000035403:ENST00000372755:exon19:c.A2755G:p.I919V,ENSG00000035403:ENST00000211998:exon20:c.A2959G:p.I987V		N	0,00057

ACTC1: actin, alpha, cardiac muscle 1; *ANK2*: ankyrin 2, neuronal; *CASQ2*: calsequestrin 2 cardiac muscle; *CAV3*: caveolin 3; *CSRP3*: cysteine and glycine-rich protein 3 cardiac LIM protein; *DES*: desmin; *DSC2*: desmocollin 2; *DSG2*: desmoglein 2; *DSP*: desmoplakin; *JUP*: junction plakoglobin; *KCNE1*: potassium voltage-gated channel, Isk-related family, member 1; *KCNE2*: potassium voltage-gated channel, Isk-related family, member 2; *KCNH2*: potassium voltage-gated channel, subfamily H eag-related, member 2; *KCNJ2*: potassium inwardly-rectifying channel, subfamily J, member 2; *KCNQ1*: potassium voltage-gated channel, KQT-like subfamily, member 1; *LDB3*: LIM domain binding 3; *LMNA*: lamin A/C; *MYBPC3*: myosin binding protein C, cardiac; *MYH6*: myosin, heavy chain 6, cardiac muscle, alpha; *MYH7*: myosin, heavy chain 7, cardiac muscle, beta; *MYL2*: myosin, light chain 2, regulatory, cardiac, slow; *MYL3*: myosin, light chain 3, alkali; *PDLIM3*: PDZ and LIM domain 3; *PKP2*: plakophilin 2; *PLN*, phospholamban; *RBM20*: RNA binding motif protein 20; *RYR2*: ryanodine receptor 2 cardiac; *SCN5A*: sodium channel, voltage-gated, type V, alpha subunit; *TCAP*: titin-cap; *TGFbeta3*: transforming growth factor, beta 3; *TMEM43*: transmembrane protein 43; *TNNC1*: troponin C type 1 slow; *TNNI3*: troponin I type 3 cardiac; *TNNT2*: troponin T type 2 cardiac; *TPM1*: tropomyosin 1 alpha; VCL: vinculin.
