# Supplementary material for: Novel genotype–phenotype associations demonstrated by high-throughput sequencing in patients with hypertrophic cardiomyopathy
Source: Heart. 2014 Oct 28;101(4):294–301. doi: 10.1136/heartjnl-2014-306387 (PMC4345808; doi:10.1136/heartjnl-2014-306387)
Supplement: Web supplement [file heartjnl-2014-306387-s3.pdf]

Supplementary table 2. Genotype-phenotype associations for individual sarcomere protein and related genes, with P-values <0.05. P-values reflect the comparison for proportions or means, between patients with and without a rare variant in a given gene.

Phenotype	Gene	Frequency or mean±standard deviation - Rare variant present	Frequency or mean±standard deviation – Rare variant absent	P-value
**Age at initial evaluation (years)**	*MYBPC3*	45.5±14.4	51.0±15.5	$8.97x10^{-6}$
	MYH7	43.9±15.4	50.5±15.2	$1.91x10^{-5}$
	TNNT2	43.5±14.7	49.8±15.4	0.031
	CSRP3	56.7±8.1	49.5±15.5	0.006
	VCL	43.2±17.6	49.8±15.3	0.0361
**Family history of HCM**	*MYBPC3*	40.4% (86/213)	21.9% (140/640)	$2.7x10^{-7}$
	MYH7	47.4% (54/114)	23.4% (173/740)	$3.06x10^{-7}$
**LA diameter (mm)**	*RBM20*	40.9±6.8	44.2±7.5	0.00239
**MLVWT (mm)**	*MYBPC3*	19.4±4.7	18.2±4.2	0.0005
**LV end-diastolic diameter (mm)**	*MYBPC3*	44.8±5.5	46.3±6.0	0.00089
	RBM20	43.6±5.2	46.1±6.0	0.00431
**LV end-systolic diameter (mm)**	*MYBPC3*	27.4±6.0	28.8±5.4	0.00512
**Fractional shortening (%)**	*MYBPC3*	39.7±8.9	38.2±8.8	0.026
**LV systolic dysfunction**	*TNNI3*	15.8% (3/19)	3.2% (26/810)	0.020
**ASH pattern**	*MYBPC3*	88.0% (184/209)	71.6% (459/641)	$3.75x10^{-6}$
	MYH7	89.2% (99/111)	73.6% (544/739)	0.00104
	MYH6	81.2% (56/69)	75.2% (587/781)	0.025
**Moderate-severe mitral regurgitation**	*MYL2*	55.6% (5/9)	19.4% (158/814)	0.041
**Right ventricular hypertrophy**	*TNNI3*	50% (10/20)	21.6% (174/806)	0.004
**LVOTO (>30 mmHg)**	*TNNI3*	10% (2/20)	41.2% (326/792)	0.00465
**LVOT gradient (mmHg)**	*TNNI3*	13.1±26.6	34.8±41.3	0.002
**Family history of SCD**	*MYBPC3*	28.7% (62/216)	18.3% (120/656)	0.001
	MYH7	31.6% (37/117)	19.2% (145/775)	0.003
	TPM1	62.5% (5/8)	20.5% (177/864)	0.012
**BP response to exercise (mmHg)**	MYH7	36.6±19.9	50.2±24.4	5.49×10^{-6}
**BP response to exercise ≤ 20 mmHg**	*MYH7*	22.5% (20/89)	12.6% (72/573)	0.020
	TNNT2	40.9% (9/22)	13.0% (83/640)	0.002
**Syncope**	*MYH7*	22.8% (26/114)	15.4% (114/742)	0.045

**Implanted cardioverter-defibrillator**	*MYH7*	28.2% (33/117)	19.2% (145/756)	0.027
**Myectomy and/or alcohol septal ablation and/or pacemaker implantation for gradient reduction**	*MYBPC3* (splicing variants)	43.1% (22/51)	20.0% (160/799)	3.0×10^{-4}

HCM: hypertrophic cardiomyopathy; NYHA: New York Heart Association; LA: left atria; MLVWT: maximum left ventricular wall thickness; LV: left ventricular; ASH: asymmetric septal hypertrophy; LVOT: left ventricular outflow tract; LVOTO: left ventricular outflow tract obstruction; SCD: sudden cardiac death; BP: blood pressure;
