# Supplementary material for: Novel genotype–phenotype associations demonstrated by high-throughput sequencing in patients with hypertrophic cardiomyopathy
Source: Heart. 2014 Oct 28;101(4):294–301. doi: 10.1136/heartjnl-2014-306387 (PMC4345808; doi:10.1136/heartjnl-2014-306387)
Supplement: Web supplement [file heartjnl-2014-306387-s4.pdf]

Supplementary table 3. Genotype-phenotype associations for individual non-sarcomere protein genes, when only analysing the sub-cohort of sarcomere-positive individuals. P-values reflect the comparison for proportions or means, between the group of patients with versus the group of patients without a rare variant in a given non-sarcomere protein gene.

**Phenotype**	**Gene**	**Frequency or mean±standard deviation -<br><br>Variant present**	**Frequency or mean±standard deviation -<br><br>Variant absent**	**P-value**
**LA diameter at last follow-up (mm)**	*SCN5A*	47.4±4.8	44.1±7.8	0.109
**LVOTO<br><br>(>30 mmHg)**	*SCN5A*	50.0% (9/18)	36.0% (122/339)	0.315
	Ion-channel	43.6% (34/78)	34.8% (97/279)	0.184
**MLVWT (mm)**	*ANK2*	19.9±6.2	18.9±4.5	0.377
**MLVWT ≥30mm**	*ANK2*	16% (4/25)	2.8% (7/251)	0.007
**E/e' ratio**	*CASQ2*	17.5±7.7	10.6±5.2	0.004
**NSVT**	*PLN*	100% (1/1)	24.7% (61/247)	0.083

LA: left atria; LVOTO: left ventricular outflow tract obstruction; MLVWT: maximal left ventricular wall thickness; E/e´ratio: ratio between the maximal velocity of the E wave from the pulsed wave Doppler of the transmitral flow and the maximal velocity of the e' wave of tissue Doppler at the mitral annulus; NSVT: non-sustained ventricular tachycardia.
